# Multimorbidity Trajectories Across Three National Ageing Cohorts: Early Branching States and Persistent Cardiometabolic-Musculoskeletal Profiles

**DOI:** 10.3390/jcm15124542

**Published:** 2026-06-11

**Authors:** Long Chen, Senyang Xiao, Yiqi Su, Hui Li, Jianhao Lin, Dan Xing

**Affiliations:** 1 Arthritis Clinic & Research Center, Peking University People’s Hospital, Peking University, Beijing 100044, China; 1910305307@pku.edu.cn (L.C.); 2411210337@stu.pku.edu.cn (S.X.); 1910301214@pku.edu.cn (Y.S.); doc_lihui@pku.edu.cn (H.L.); linjianhao@pkuph.edu.cn (J.L.); 2 Arthritis Institute, Peking University, Beijing 100044, China; 3 Peking University Health Science Center, Beijing 100044, China

**Keywords:** multimorbidity, comorbidity, chronic disease, longitudinal studies, cohort studies, ageing, disease progression, cardiometabolic risk factors, arthritis

## Abstract

**Background/Objectives**: Disease count alone does not show which multimorbidity combinations diversify or persist. We examined longitudinal changes in clinically recognisable multimorbidity profiles across three national ageing cohorts. **Methods**: Harmonised data from the China Health and Retirement Longitudinal Study (CHARLS), the English Longitudinal Study of Ageing (ELSA), and the U.S. Health and Retirement Study (HRS) were analysed. Eight physician-diagnosed chronic conditions were encoded as binary states, and wave-to-wave transitions (four windows in CHARLS and ELSA; five in HRS) were assessed within each cohort. State-level measures characterised accumulation, branching, persistence, and stabilisation sensitivity, supplemented by sensitivity analyses, BranchScore decomposition, prevalence-adjusted enrichment, and a disease-count-preserving permutation null model. **Results**: The analysis included 17,142 CHARLS, 10,272 ELSA, and 22,034 HRS participants, with baseline multimorbidity of 23.7%, 41.0%, and 58.5%, respectively. Transitions are concentrated around common profiles. One- and two-condition states (hypertension, diabetes, heart disease, chronic lung disease, psychiatric or emotional disorders) showed faster accumulation and greater branching; later persistent states were dominated by cardiometabolic-musculoskeletal combinations, particularly hypertension-heart disease-arthritis and hypertension-diabetes-arthritis. Targeted stabilisation produced modest perturbations exceeding random benchmarks. Count-preserving null models showed that early branching was largely structural, whereas selected lock-in states exceeded null expectations. **Conclusions**: Across three ageing cohorts, multimorbidity trajectories reflected disease composition as well as count. Because branching was strongly influenced by disease-count geometry, early branching states should be interpreted as structural cohort-level features of the cumulative framework rather than inherently predictive clinical entities. Selected cardiometabolic-musculoskeletal profiles were more persistent and may help frame integrated long-term management; patient-level prediction requires outcome-based studies.

## 1. Introduction

Multimorbidity, usually defined as the coexistence of two or more chronic conditions, is now routine in the care of older adults and is closely linked to functional decline, poorer quality of life, greater health-care use, and higher mortality [1,2,3,4,5]. The composition of multimorbidity also shapes downstream clinical decisions, including perioperative and procedural risk assessment in older patients with combined chronic disease and frailty [4,6]. Yet clinical practice and much of the evidence base remain organised around single diseases. General internists, therefore, need to manage hypertension, diabetes, heart disease, chronic lung disease, arthritis, cancer, stroke, and psychiatric or emotional disorders not only as separate diagnoses, but as combinations that change over time [7].

Disease count is useful because it offers a simple measure of burden and allows comparison across settings [5,8]. Its limitation is that two patients with the same number of diagnoses may have very different clinical profiles, treatment priorities, and future risks [5,9]. Hypertension with diabetes is not clinically equivalent to chronic lung disease with arthritis, even though both represent two-condition multimorbidity. Measures that retain disease composition may therefore add clinically relevant information beyond the number of conditions alone.

This distinction matters in internal medicine, where management is rarely determined by count alone [10]. Treatment burden, surveillance needs, drug interactions, functional limitation, and patient priorities depend on which conditions cluster together [10,11]. A count of two or three conditions can describe overall load, but it does not show whether a patient is entering a cardiometabolic pathway, developing a cardio-respiratory-musculoskeletal profile, or remaining in a relatively stable combination that requires coordinated long-term follow-up [12,13,14].

A longitudinal view is needed because multimorbidity develops over time [13,14]. Some early combinations may be branching points from which later profiles diverge, whereas other combinations may become persistent after cardiometabolic and musculoskeletal burden has accumulated. Identifying these profiles could help clinicians recognise earlier stages of complexity, anticipate follow-up needs, and plan integrated chronic disease management before multimorbidity becomes highly entrenched [13].

In this study, lock-in denotes low downstream diversification and relative persistence of a cumulative disease profile, whereas stabilisation sensitivity describes how much the transition structure changes in a simulation that increases the self-transition tendency of selected states.

We analysed multimorbidity as transitions between disease combinations using harmonised longitudinal data from national ageing cohorts in China, England, and the United States [15,16]. The objective was to identify clinically recognisable states associated with rapid accumulation, downstream branching, or later persistence, and to assess whether these roles recurred across populations with different baseline disease burdens.

## 2. Materials and Methods

### 2.1. Study Design and Data Sources

**Figure 1:**
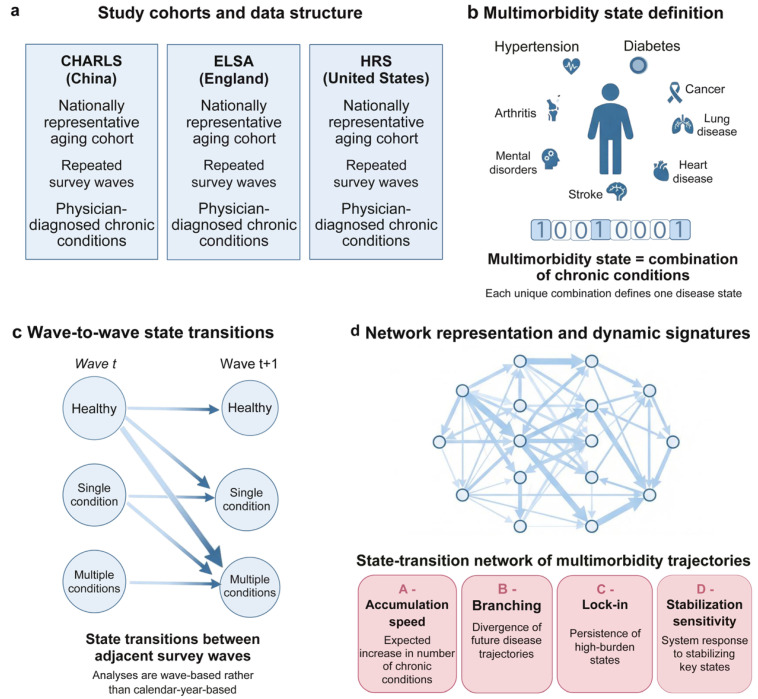
Conceptual framework for modelling multimorbidity transitions across national ageing cohorts. The figure summarises the three longitudinal ageing cohorts, the derivation of binary multimorbidity states from eight physician-diagnosed chronic conditions, wave-to-wave transitions between adjacent surveys, and the four trajectory measures used to describe accumulation, branching, persistence, and stabilisation sensitivity. Panel (**a**) shows the study cohorts and data structure; panel (**b**) shows the multimorbidity state definition; panel (**c**) shows wave-to-wave state transitions; and panel (**d**) shows the network representation and dynamic signatures. CHARLS, China Health and Retirement Longitudinal Study; ELSA, English Longitudinal Study of Ageing; HRS, Health and Retirement Study. Lock-in denotes relative persistence with limited downstream diversification; stabilisation sensitivity denotes the simulated change in transition structure after increasing self-transition probability for selected states.

We analysed three nationally representative longitudinal ageing cohorts: the China Health and Retirement Longitudinal Study (CHARLS), the English Longitudinal Study of Ageing (ELSA), and the U.S. Health and Retirement Study (HRS). Each cohort collects repeated information on physician-diagnosed chronic conditions (Figure 1). The first eligible wave around 2010–2011 was defined as the analytic baseline: raw wave 1 in CHARLS, raw wave 5 in ELSA, and raw wave 10 in HRS. Subsequent waves were recoded relative to this analytic baseline. Transitions were constructed only between adjacent analytic survey waves among participants observed with complete disease data at both waves; transitions spanning a missing intermediate wave were not imputed.

The three cohorts were used to examine whether broad transition roles were visible across populations with different baseline disease burdens and health-care contexts. Cohorts were not pooled because the aim was not to estimate a single average transition probability across countries. Each cohort was analysed separately and then compared descriptively to identify recurring state roles relevant to clinical medicine rather than to one national cohort alone.

### 2.2. Chronic Conditions and Multimorbidity States

Eight physician-diagnosed chronic conditions were included because they were available with comparable definitions across all three cohorts: hypertension, diabetes, cancer, chronic lung disease, heart disease, stroke, psychiatric or emotional disorders, and arthritis or rheumatism. This harmonised set captured common cardiometabolic, respiratory, mental health, malignant, cerebrovascular, and musculoskeletal conditions relevant to older adults, but it did not include other clinically important ageing-related conditions such as chronic kidney disease, osteoporosis, or cognitive impairment. At each wave, disease status was represented by a binary vector. Unique combinations of the eight conditions were defined as multimorbidity states; the state 00,000,000 represented no recorded condition. Disease count was the sum of conditions in the vector.

The eight-condition framework yields a possible state space of 256 profiles. In practice, only a subset of these profiles was observed in each cohort and window. This sparsity is clinically informative because it indicates that progression was concentrated around recurring combinations rather than distributed evenly across all theoretically possible profiles.

### 2.3. State-Transition Analysis

For each cohort and transition interval, movement from state i at wave t to state j at wave t + 1 was tabulated. Nodes represented observed multimorbidity states, and directed edges represented observed transitions. Edge weights were the number of participants moving between states. Because the included diagnoses were treated as chronic conditions and apparent disappearance may reflect reporting variation, a disease-level irreversibility rule was applied: once a condition had been recorded, it was carried forward in later waves. The resulting states should therefore be interpreted as cumulative diagnostic-history profiles rather than purely contemporaneous clinical status. This assumption is central to the transition framework, and its influence was evaluated in sensitivity analyses without the carry-forward rule (Figure S4; Table S6).

This approach treats each observed disease combination as a clinically recognisable profile at a given time and each transition as a change in that profile over follow-up. Network terminology is used as a compact way to describe movement between profiles.

Transition probabilities were estimated empirically as follows:

$$P\left(j|i\right)=\frac{N_{ij}}{\sum_{k}N_{ik}},$$

where N_ij is the number of individuals moving from state i to state j, and the denominator is the total number of individuals in state i at the start of the interval.

### 2.4. Trajectory Measures

Each state’s outgoing transition distribution was summarised using four measures.

Accumulation speed was defined as the expected one-interval increase in disease count:

$$\mathrm{AccumSpeed}\left(i\right)=\sum_{j}P\left(j|i\right)\cdot \left({\mathrm{Count}}_{j}-{\mathrm{Count}}_{i}\right).$$

Branching was used as a descriptive measure of downstream diversification. It combined outgoing transition entropy with the variance in downstream disease counts:

$$\mathrm{BranchScore}\left(i\right)=H\left(i\right)\sqrt{\mathrm{Var}\left(Count_{j}\right)}.$$

Persistence was assessed using a lock-in score:

$$\mathrm{LockInScore}\left(i\right)=\left(1-\mathrm{EntropyNorm}\left(i\right)\right)\left(Count_{i}+1\right).$$

Normalised entropy was defined as follows:

$$\mathrm{EntropyNorm}\left(i\right)=\frac{H\left(i\right)}{\log\left(out\_degree_{i}\right)}$$

$$H\left(i\right)=-\sum_{j}P\left(j|i\right)\;\log P\left(j|i\right)$$

when out_degree_i > 1 and as 0 otherwise.

These measures were selected because they correspond to distinct clinical questions. Accumulation speed asks whether participants in a profile tend to acquire additional diagnoses over the next interval. Branching asks whether a profile is followed by one dominant pathway or several downstream profiles with differing disease burden. Lock-in asks whether a profile tends to persist once reached. Stabilisation sensitivity asks whether changing the self-transition tendency of selected states would alter the transition structure. These measures describe cohort-level transition architecture and are not intended for individual risk prediction.

### 2.5. Stabilisation Sensitivity

To assess whether particular states had disproportionate influence on long-run trajectory structure, two targeted stabilisation scenarios were compared within each cohort window: increasing self-transition probability by a fixed increment (alpha = 0.30) for the five highest-branching states, or for the five states with the highest accumulation speed. Remaining outgoing probabilities were reduced proportionally so that each transition row summed to 1. The resulting distribution was compared with the baseline distribution using L1 distance. Further sensitivity analyses compared alternative alpha values with a random eligible-state benchmark using a support-weighted transition-matrix L1 distance. Specifically, the row-wise L1 distance between perturbed and baseline transition matrices was averaged with weights proportional to from-state support, so that frequently visited states contributed more to the overall distance. For each source state, the row-wise L1 distance was calculated as:

$$L_{1}=\sum_{s}\left|p_{\mathrm{stabilized}}\left(s\right)-p_{\mathrm{baseline}}\left(s\right)\right|.$$

### 2.6. Cross-Cohort Reproducibility

For cross-cohort comparisons, analyses were restricted to states with at least 50 from-state observations within a cohort window. States were ranked by each dynamic measure and expressed as within-window percentile ranks. Rankings were summarised across windows within each cohort using from-state support as weights. States recurring in at least two cohorts were treated as reproducible key states. Sensitivity analyses examined thresholds of 30, 50, and 100 from-state observations (Table S7). To separate dynamic behaviour from state-space geometry, accumulation, branching, and lock-in scores were regressed on disease count and log-transformed from-state support within each cohort window, using from-state support as weights; residual scores were used to identify states whose dynamic rank exceeded what disease count and support alone would predict. Additional checks decomposed BranchScore into entropy-only and downstream disease-count-variance ranks (Table S11). We also compared observed profile counts with independent-prevalence expectations (Table S12) and implemented a disease-count-preserving permutation null model that preserved each transition’s from-state and target disease count while randomising target disease-combination identity among compatible irreversible states (Table S13).

### 2.7. Statistical Analysis

All analyses were conducted separately within each cohort using R version 4.3.3 (R Foundation for Statistical Computing, Vienna, Austria) in RStudio version 2024.12.1+563 (Posit Software, PBC, Boston, MA, USA). The study was descriptive and was designed to characterise transition patterns rather than estimate causal effects between diseases.

## 3. Results

### 3.1. Cohort Characteristics and Baseline Multimorbidity

The analysis included 17,142 participants in CHARLS, 10,272 in ELSA, and 22,034 in HRS (Table 1). Participants in CHARLS were younger on average (58.9 years) than those in ELSA (66.7 years) and HRS (65.7 years). Women accounted for 52.0% of participants in CHARLS, 55.5% in ELSA, and 58.2% in HRS.

Baseline multimorbidity burden differed across cohorts. The mean number of chronic conditions increased from 0.91 in CHARLS to 1.39 in ELSA and 2.01 in HRS. The proportions with multimorbidity were 23.7%, 41.0%, and 58.5%, respectively, while the proportion with no recorded chronic condition was 42.2%, 27.2%, and 17.3%. Hypertension and arthritis were the most prevalent conditions in all cohorts. Hypertension affected 25.9% of participants in CHARLS, 42.7% in ELSA, and 56.5% in HRS; arthritis ranged from 32.4% to 53.1%. Diabetes and heart disease were more common in HRS, while psychiatric or emotional disorders were uncommon in CHARLS but reported more often in ELSA and HRS.

### 3.2. Overall Transition Structure

**Figure 2:**
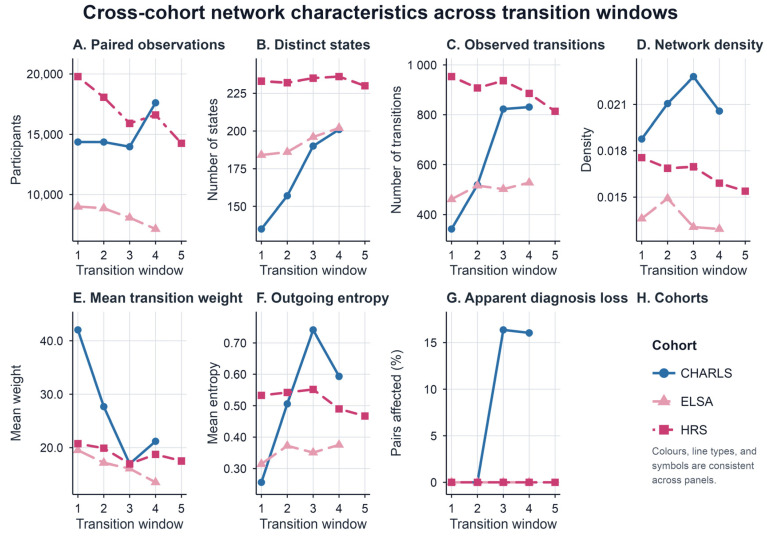
Evolution of multimorbidity state-transition networks across survey windows. Network characteristics of multimorbidity transitions across consecutive survey waves in CHARLS, ELSA, and HRS are shown. Panels summarise the number of participants, distinct disease profiles, observed transitions, network density, average transition weight, dispersion of next-state destinations, and apparent loss of previously recorded diagnoses. Cohort colours, line types, and symbols are defined in the legend panel. Across cohorts, transition networks remained sparse and concentrated around common states. Panels show (**A**) paired observations, (**B**) distinct states, (**C**) observed transitions, (**D**) network density, (**E**) mean transition weight, (**F**) outgoing entropy, (**G**) apparent diagnosis loss, and (**H**) cohort legend.

Multimorbidity transitions were sparse but heterogeneous (Figure 2). CHARLS showed an increase in observed states from approximately 130 in the earliest window to nearly 200 in later windows. ELSA had relatively stable state counts, and HRS maintained the largest state space throughout follow-up. The number of observed transitions increased in CHARLS, remained broadly stable in ELSA, and declined modestly in HRS.

These patterns were consistent with different stages of cohort-level multimorbidity burden. CHARLS began with a lower mean disease count and showed expansion of observed profiles over follow-up, suggesting a period in which new combinations were still emerging. HRS began with a higher mean burden and maintained a broader state space from the start, suggesting a more established distribution of complex profiles. ELSA occupied an intermediate position.

Despite these differences, network density remained low in all cohorts and windows, indicating that only a small fraction of possible transitions occurred in practice. Network visualisations showed a dense central cluster of common states surrounded by less frequent peripheral profiles (Figure S1). Degree and weighted-degree distributions were strongly right-skewed (Figure S2), meaning that most states had few connections while a small number accounted for much of the observed transition activity.

Mean outgoing transition entropy increased during mid-follow-up and then declined in CHARLS, suggesting that trajectories first became more dispersed and then narrowed later. Entropy levels in ELSA and HRS were lower and more stable. Mean edge weight decreased over time in CHARLS, indicating that transitions were distributed across a larger number of pathways. Sensitivity analyses showed that newly reported conditions were observed in 10.4–35.6% of paired transitions across windows. Apparent losses of previously recorded diagnoses were not observed after harmonised binary coding in ELSA and HRS, but were present in later CHARLS windows (16.4% in wave 3 to wave 4 and 16.0% in wave 4 to wave 5), supporting a conservative interpretation of the carry-forward rule (Table S5).

### 3.3. Early Divergence from Low-Burden States

**Figure 3:**
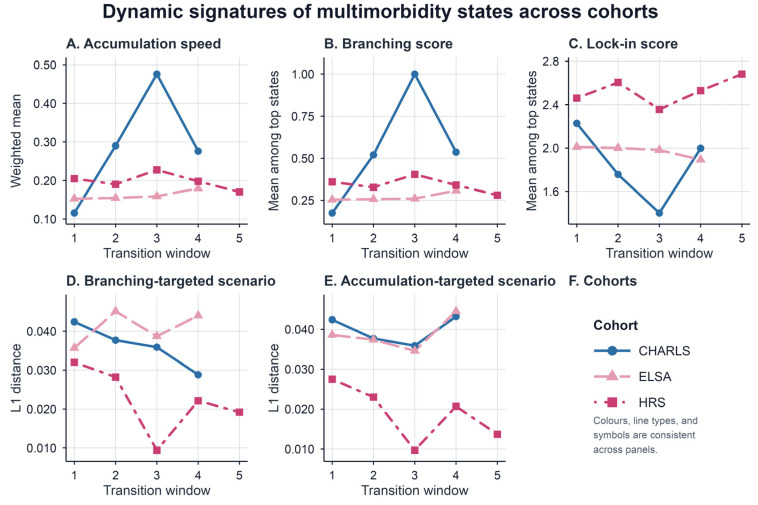
Cross-cohort comparison of accumulation, branching, persistence, and stabilisation sensitivity. Panels show weighted mean accumulation speed, mean branching score among highest-branching states, mean lock-in score among highest lock-in states, and L1 distance under branching-targeted and accumulation-targeted stabilisation scenarios. Panels show (**A**) weighted mean accumulation speed, (**B**) mean branching score among the highest-branching states, (**C**) mean lock-in score among the highest lock-in states, (**D**) L1 distance under the branching-targeted stabilisation scenario, (**E**) L1 distance under the accumulation-targeted stabilisation scenario, and (**F**) cohort legend. Cohort colours, line types, and symbols are defined in the legend panel. Results are shown by cohort and harmonised transition window. Per-cohort state-level detail is provided in Figure S3A–C.

Across cohorts, the most dynamic states were usually early and clinically familiar profiles rather than highly complex combinations. Accumulation speed was highest in CHARLS and peaked during mid-follow-up (Figure 3). In ELSA, it was comparatively stable, while HRS showed moderate variation. States with the highest accumulation speeds generally contained one or two conditions (Table S1A). In CHARLS, these included heart disease, diabetes, chronic lung disease, hypertension, hypertension-heart disease, and diabetes-arthritis. The disease-free state also frequently appeared among the faster-accumulating states.

The relation between disease count and accumulation speed was non-linear (Table S1B). In CHARLS wave 3 to wave 4, mean accumulation speed increased from 0.436 in the disease-free state to 0.482 among one-condition states and peaked at 0.512 among two-condition states before declining among states with three or more conditions. Similar patterns were observed in ELSA and HRS, where one- or two-condition states generally accumulated additional disease more quickly than more complex states.

Branching followed a similar early-stage pattern. The highest-branching states were usually common and low-burden profiles, including the disease-free state, hypertension, diabetes, heart disease, chronic lung disease, psychiatric or emotional disorders, and simple dyads such as hypertension-heart disease and hypertension-diabetes (Table S2A). These findings suggest that later heterogeneity in multimorbidity often begins from common clinical starting points, rather than only after complex multimorbidity is already present.

The overlap between fast-accumulating and high-branching states was substantial but incomplete. Across the cohort window, the top-10 Jaccard overlap between accumulation speed and branching ranged from 0.54 to 0.82, corresponding to seven to nine shared states in most windows (Table S9). Some states accumulated additional diagnoses quickly but moved towards a relatively limited set of downstream profiles, whereas others branched into more heterogeneous pathways. This distinction is relevant clinically because a profile that appears modest by disease count may still represent a cohort-level point at which future observed profiles become more heterogeneous.

### 3.4. Later Persistence of Cardiometabolic-Musculoskeletal Profiles

Persistence showed a contrasting pattern. Mean lock-in among the highest-ranking states was greatest in HRS, intermediate in ELSA, and lower in CHARLS (Figure 3). High lock-in states were dominated by hypertension-centred combinations, often accompanied by arthritis and cardiometabolic disease (Table S2B). In ELSA, the most consistent high lock-in state was hypertension-heart disease-arthritis. Other recurrent states included hypertension-diabetes-arthritis and hypertension-arthritis.

In CHARLS, high lock-in states shifted towards more complex cardiometabolic profiles in later windows; by wave 4 to wave 5, the highest lock-in state was hypertension-diabetes-heart disease-arthritis. In HRS, high lock-in states generally had higher disease burden and often included four or five conditions involving hypertension, diabetes, heart disease, stroke, cancer, and arthritis. These findings indicate a shift from early pathway diversification to later persistence within cardiometabolic-musculoskeletal profiles.

Mean branching and lock-in scores among top states also illustrated this shift. CHARLS showed a mid-follow-up peak in branching followed by stronger lock-in later. ELSA remained comparatively stable, while HRS showed consistently high lock-in together with moderate branching. The recurrent involvement of arthritis in high lock-in profiles suggests that musculoskeletal disease may be an important component of persistent multimorbidity, not merely a background condition common in older adults.

### 3.5. State-Level Roles and Reproducible Anchors

**Figure 4:**
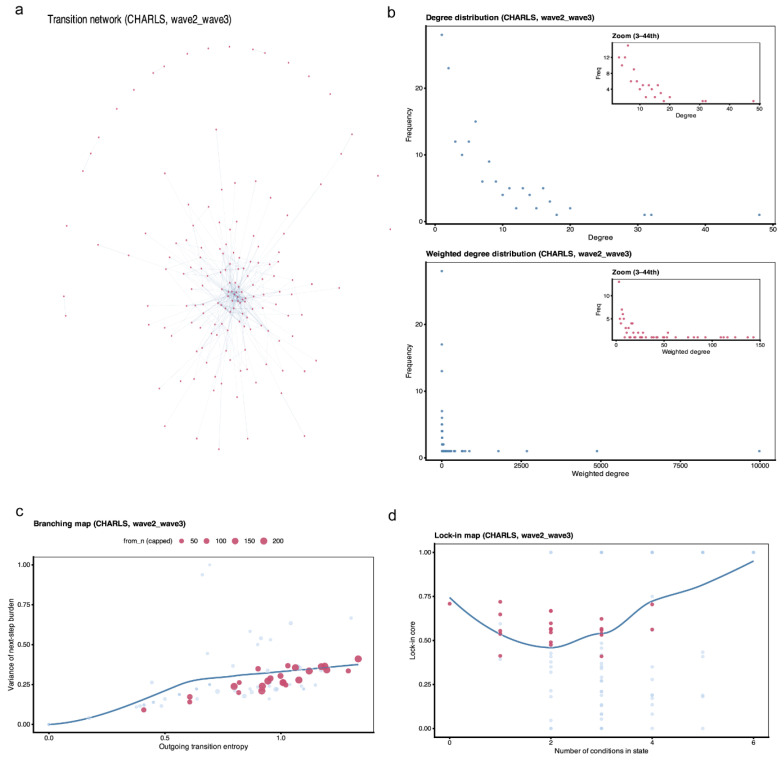
Representative state-transition network and state-level roles in CHARLS wave 2 to wave 3. The network illustrates a representative active phase of multimorbidity progression. Panels show the overall state-transition network, degree distribution, high-branching states with diverse outgoing transitions, and high lock-in states representing more persistent disease combinations. Panel (**a**) shows the representative transition network; panel (**b**) shows the degree and weighted-degree distributions, including zoomed views; panel (**c**) shows the branching map based on outgoing transition entropy and downstream burden variance; and panel (**d**) shows the lock-in map by disease count.

The CHARLS wave 2 to wave 3 network illustrates the state-level structure of progression (Figure 4). A small number of states acted as hubs within a dense central cluster. Branching was closely related to outgoing transition entropy, but it also reflected differences in downstream disease burden. Lock-in was not determined by disease count alone; both lower- and higher-burden states could persist, whereas some intermediate states continued to transition. Disease-count stratified and support-adjusted analyses indicated that count influenced the dynamic measures but did not fully explain the ranking of individual states, although residual state-space effects may remain (Figure S5; Table S8A,B).

Additional component and null-model analyses qualified this interpretation. Across 13 cohort windows, the top-10 BranchScore states overlapped strongly with entropy-only rankings (median overlap, 9 states; range, 8–10) and downstream-burden-variance rankings (median overlap, 9 states; range, 7–10), indicating that the composite score was closely related to both components but not identical to either alone (Table S11). In prevalence-adjusted enrichment analyses, 155 of 413 eligible state-window rows were enriched at FDR < 0.05; the median observed-to-expected ratio was 1.01 (IQR, 0.74–1.51), with the strongest enrichments in high-burden HRS profiles including hypertension, diabetes, chronic lung disease, heart disease, psychiatric or emotional disorders, and arthritis (Table S12).

In the disease-count-preserving permutation null model, no observed top-10 BranchScore states exceeded the null 95th percentile (median, 0 per cohort window; range, 0–0), suggesting that early branching was strongly shaped by disease-count geometry within the irreversible cumulative framework. By contrast, some high-ranking LockInScore states exceeded null expectations (median, 2 per cohort window; range, 0–4), supporting a cautious interpretation that selected persistent profiles may contain disease-combination information beyond disease count alone (Table S13).

**Figure 5:**
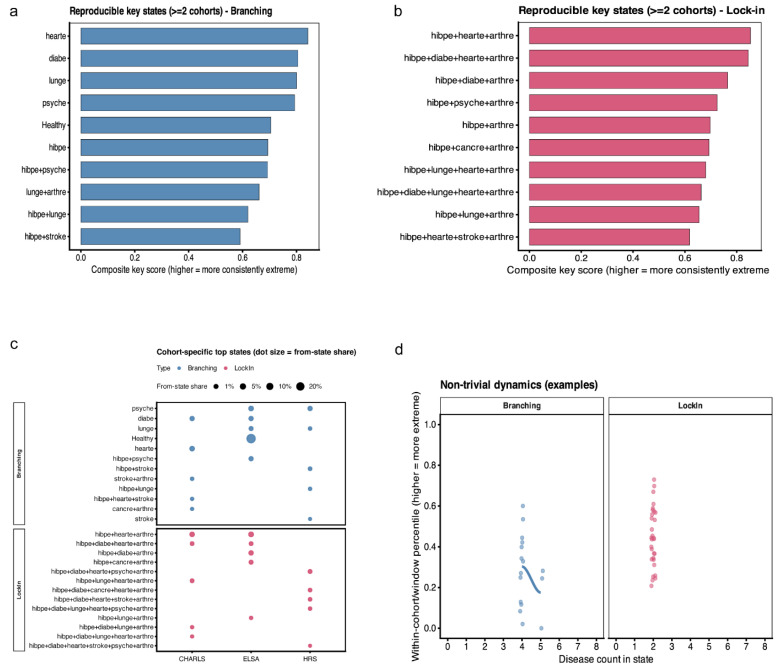
Reproducible multimorbidity states and trajectory roles across cohorts. Panels show (**a**) reproducible branching states, (**b**) reproducible lock-in states, (**c**) cohort-specific top-ranked branching and lock-in states, and (**d**) examples of non-trivial dynamic extremeness by disease count. Dot size is proportional to the from-state share. The role of a state depended on disease composition as well as disease burden.

Several trajectory roles recurred across cohorts (Figure 5; Table S3). Reproducible branching states were mainly low-burden profiles, including heart disease, diabetes, chronic lung disease, hypertension, psychiatric or emotional disorders, and the disease-free state. Reproducible high lock-in states were concentrated in cardiometabolic-musculoskeletal combinations, particularly hypertension-heart disease-arthritis and hypertension-diabetes-arthritis. The four-condition combination of hypertension, diabetes, heart disease, and arthritis reached the highest lock-in score observed in the analysis.

Many recurrent key states also had substantial from-state support, indicating that they were not rare analytical outliers. These recurrent profiles correspond to familiar constellations of chronic disease in older adults that already shape medication review, cardiovascular risk management, pain and mobility assessment, and follow-up intensity.

### 3.6. Structural Robustness

Targeted stabilisation simulations produced modest perturbations in long-run state distributions (Table S4). Across cohorts and windows, L1 distances ranged from 0.0094 to 0.0451. Stabilising branching states produced L1 distances of 0.0288 to 0.0424 in CHARLS, 0.0346 to 0.0451 in ELSA, and 0.0094 to 0.0320 in HRS. Corresponding changes when targeting the fastest-accumulating states were similar in magnitude. Further alpha-sensitivity analyses showed that support-weighted transition-matrix L1 distances increased with alpha and generally exceeded the random eligible-state benchmark for targeted states (Figure S6; Table S10).

The small magnitude of the long-run perturbations should temper interpretation. These analyses do not imply that a single clinical intervention at one state would transform population-level multimorbidity. They are better read as structural diagnostics indicating that some common branching states have somewhat larger influence on the observed transition architecture than states selected only because their disease count rises quickly over one interval.

## 4. Discussion

In this longitudinal analysis of three national ageing cohorts, multimorbidity evolved through clinically recognisable disease profiles rather than through disease count alone. Early low-burden states, including hypertension, diabetes, heart disease, chronic lung disease, psychiatric or emotional disorders, and simple dyads, were more likely to accumulate further disease and branch into different downstream profiles. Later persistent states were dominated by cardiometabolic-musculoskeletal combinations, especially profiles involving hypertension, diabetes, heart disease, and arthritis.

These findings support the view that the clinical meaning of multimorbidity depends on composition as well as burden [12,17,18]. Patients with the same number of diagnoses may differ in prognosis, treatment complexity, monitoring needs, and vulnerability to functional decline [17,19]. The present analysis extends this idea longitudinally: states with similar counts did not occupy the same role in the transition structure. Some low-burden profiles opened into multiple possible pathways, whereas some higher-burden profiles were more stable and persistent.

The early branching pattern is clinically important because general internists often encounter patients with one or two common chronic conditions long before severe multimorbidity is established. In this study, hypertension and diabetes were frequent branching states, consistent with their central role in cardiometabolic multimorbidity. Rather than being only markers of existing burden, such conditions may mark cohort-level profiles in which observed future disease combinations are more heterogeneous.

The additional count-preserving null analysis required a more conservative interpretation of the early branching result. Low-burden states had more possible downstream combinations in the finite irreversible state space, and the observed BranchScore rankings did not exceed the disease-count-preserving null distribution. Thus, early branching should be read primarily as a structural feature of the cumulative transition framework, not as evidence that those profiles independently generate clinically open futures.

These population-level findings should not be read as deterministic sequences or validated risk predictions for individual patients. Their value is in drawing attention to common profiles in which routine follow-up may need to include broader assessment: emerging cardiometabolic disease, respiratory symptoms, musculoskeletal pain and function, and mental health, rather than isolated disease-specific monitoring alone.

The later persistence of cardiometabolic-musculoskeletal combinations has a different clinical implication. Profiles involving hypertension, diabetes, heart disease, and arthritis combine vascular risk, symptom burden, pain, mobility limitation, medication complexity, and competing treatment priorities [11,12,13,18]. The repeated presence of arthritis in persistent states is notable. In older adults, arthritis may complicate physical activity, self-management, and functional reserve, and may therefore help sustain long-term complexity once cardiometabolic disease has accumulated [20,21].

For clinical management, these persistent profiles are less about early prevention of any single condition and more about long-term integration of care. They may require medication reconciliation, renal and cardiovascular monitoring, attention to analgesic safety, support for physical activity within functional limits, and coordination across disease-specific recommendations [22]. The present study cannot test whether such care models improve outcomes, but it helps identify the kinds of profiles for which single-disease pathways are likely to be insufficient.

The cross-cohort pattern was consistent despite differences in baseline disease burden. CHARLS appeared to capture a more active phase of early accumulation and branching, HRS showed stronger persistence of higher-burden profiles, and ELSA was generally intermediate. Similar trajectory roles may therefore emerge at different stages of population-level multimorbidity development. For clinicians, the practical message is not that a single sequence applies to all patients, but that common early combinations and later cardiometabolic-musculoskeletal profiles deserve attention across health-care settings.

Cross-cohort reproducibility also strengthens the clinical interpretation. The cohorts differ in population composition, disease prevalence, and health-care context, yet similar roles recurred. At the same time, differences between cohorts may reflect age structure, baseline disease burden, diagnostic access, reporting behaviour, and health-system context. This balance between reproducibility and local variation is consistent with the way clinicians encounter multimorbidity: recognisable patterns recur, but their timing and burden differ across patients and settings.

The stabilisation analyses should be interpreted descriptively. They do not show that intervening on a state would prevent later disease. However, they indicate that selected high-branching states can produce larger structural perturbations than randomly selected eligible states in the transition matrix. This supports a cautious clinical focus on early profiles that lead to multiple downstream pathways, while recognising that intervention effectiveness must be tested in outcome-oriented studies.

The state-transition framework was useful because it separated three roles that disease count tends to merge: fast accumulation, branching into heterogeneous futures, and persistence once complex cardiometabolic-musculoskeletal disease is established. Together, these distinctions translate more directly into clinical follow-up priorities than disease count alone.

This study has several strengths. It used harmonised longitudinal data from three large national cohorts and examined specific disease combinations rather than relying only on counts. The state-transition framework distinguished accumulation, branching, persistence, and structural influence, allowing clinically interpretable roles to be assigned to common multimorbidity profiles.

Several limitations should be considered. Chronic conditions were based on self-reported physician diagnoses and may be affected by diagnostic access, recall, and reporting differences across countries [23]. The analysis was restricted to eight harmonised conditions and therefore cannot capture the full clinical complexity of older adults, including chronic kidney disease, osteoporosis, cognitive impairment, frailty, and other conditions that may alter multimorbidity pathways. Follow-up intervals and measurement procedures differed across cohorts, which may affect direct comparability. Disease prevalence and from-state support also shaped the transition graph; high-prevalence conditions naturally generated more observed dyads and triads. We therefore used support thresholds and count/support-adjusted checks, but residual prevalence effects may remain.

Additional limitations relate to the state definition and interpretation. The irreversibility assumption is clinically plausible for many included diagnoses and reduces bias from inconsistent reporting, but it also means that the analysis represents cumulative diagnostic history rather than contemporaneous disease status. This choice can increase apparent persistence and may particularly affect conditions with fluctuating symptoms or reporting, such as psychiatric or emotional disorders, chronic lung disease, and arthritis. Sensitivity analyses without the carry-forward rule changed the top-ranked accumulation and branching states more than the lock-in states (Figure S4; Table S6), reinforcing the need to interpret early branching as a property of the chosen cumulative-history framework. Because the analysis was confined to eight conditions, profiles with fewer diagnoses have more possible downstream combinations than profiles with more diagnoses, which can contribute to higher apparent branching for low-burden states. Count-stratified and support-adjusted analyses reduced but did not fully remove this effect. Finally, disease severity, medication use, laboratory values, frailty, functional status, and health-care utilisation were not incorporated into the state definitions. The profiles should therefore be understood as broad clinical combinations rather than detailed phenotypes suitable for individual prognostication. Accordingly, lock-in may partly reflect administrative or record-based accumulation rather than stable biological persistence of every condition at every wave. The disease-count-preserving permutation analysis further indicated that the branching pattern was largely compatible with the geometry of irreversible disease-count accumulation, whereas selected persistent profiles showed stronger evidence of disease-combination identity beyond disease count alone.

## 5. Conclusions

Multimorbidity trajectories across three national ageing cohorts were shaped by the composition of chronic disease profiles. Common early low-burden states were associated with faster accumulation and greater branching within the cumulative transition framework, although the branching pattern was strongly shaped by disease-count geometry. Selected later cardiometabolic-musculoskeletal combinations were more persistent, and some exceeded disease-count-preserving null expectations. For clinical medicine, these findings show why disease count alone is insufficient and suggest that recognisable profiles may help frame earlier complexity and later long-term management needs, while further outcome-based studies are needed before patient-level risk stratification or intervention decisions can be derived from this framework.

## Figures and Tables

**Table 1:** Baseline characteristics of participants in CHARLS, ELSA, and HRS.

Cohort	CHARLS	ELSA	HRS
N	17142	10272	22034
Age	58.9 (10.2)	66.7 (9.9)	65.7 (12.0)
Female	0.52	0.555	0.582
Mean number of conditions	0.91 (1.00)	1.39 (1.22)	2.01 (1.51)
No chronic conditions	0.422	0.272	0.173
Multimorbidity (count ≥ 2)	0.237	0.41	0.585
Hypertension	0.259	0.427	0.565
Diabetes	0.061	0.107	0.215
Cancer	0.009	0.097	0.134
Chronic lung disease	0.097	0.061	0.09
Heart disease	0.121	0.187	0.219
Stroke	0.028	0.046	0.084
Psychiatric or emotional disorders	0.014	0.097	0.171
Arthritis	0.324	0.369	0.531

Values are mean (SD) or proportions unless otherwise indicated. CHARLS, China Health and Retirement Longitudinal Study; ELSA, English Longitudinal Study of Ageing; HRS, Health and Retirement Study.

## Data Availability

The data analysed in this study are available from the original cohort providers, subject to their data access policies. CHARLS data are available through the CHARLS official data platform, ELSA data through the UK Data Service, and HRS data through the University of Michigan Health and Retirement Study data portal for registered users.

## Supplementary Materials

The following supporting information can be downloaded at: https://www.mdpi.com/article/10.3390/jcm15124542/s1, Figure S1: State-transition network visualisations across cohorts and survey windows; Figure S2: Degree and weighted-degree distributions; Figure S3A: State-level dynamic signatures in CHARLS; Figure S3B: State-level dynamic signatures in ELSA; Figure S3C: State-level dynamic signatures in HRS; Figure S4: Sensitivity of top-ranked states without the irreversibility rule; Figure S5: Disease-count stratified dynamic measures; Figure S6: Stabilisation alpha sensitivity and random eligible-state benchmark; Table S1A: States with the highest accumulation speed across cohorts and survey windows; Table S1B: Accumulation speed summarised by baseline disease count across cohorts and survey windows; Table S2A: Top branching states; Table S2B: Top lock-in states; Table S2C: Cohort-window summary of dynamic measures; Table S3: Recurrent top states across cohorts; Table S4: Stabilisation-sensitivity L1 distances; Table S5: Carry-forward and newly reported condition counts; Table S6: Sensitivity analysis without the irreversibility rule; Table S7: From-state support threshold sensitivity; Table S8A: Disease-count-stratified dynamic measures in the primary irreversible analysis; Table S8B: Count/support-adjusted top states and residual dynamic rankings; Table S9: Overlap between fast-accumulating and high-branching states; Table S10: Stabilisation alpha and random eligible-state benchmark.; Table S11: BranchScore component rankings among top-ranked branching states; Table S12: Prevalence-adjusted enrichment of multimorbidity profiles; Table S13: Disease-count-preserving permutation null model for top-ranked BranchScore and LockInScore states.
