# Supplementary material for: Multimorbidity Trajectories Across Three National Ageing Cohorts: Early Branching States and Persistent Cardiometabolic-Musculoskeletal Profiles
Source: J Clin Med. 2026 Jun 11;15(12):4542. doi: 10.3390/jcm15124542 (PMC13302570; doi:10.3390/jcm15124542)
Supplement: Supplementary file 1 [file jcm-15-04542-s001.zip › jcm-4329345-supplementary.pdf]

Supplementary Materials

Multimorbidity Trajectories Across Three National Ageing Cohorts: Early Branching States and Persistent Cardiometabolic-Musculoskeletal Profiles

Supplementary Figures

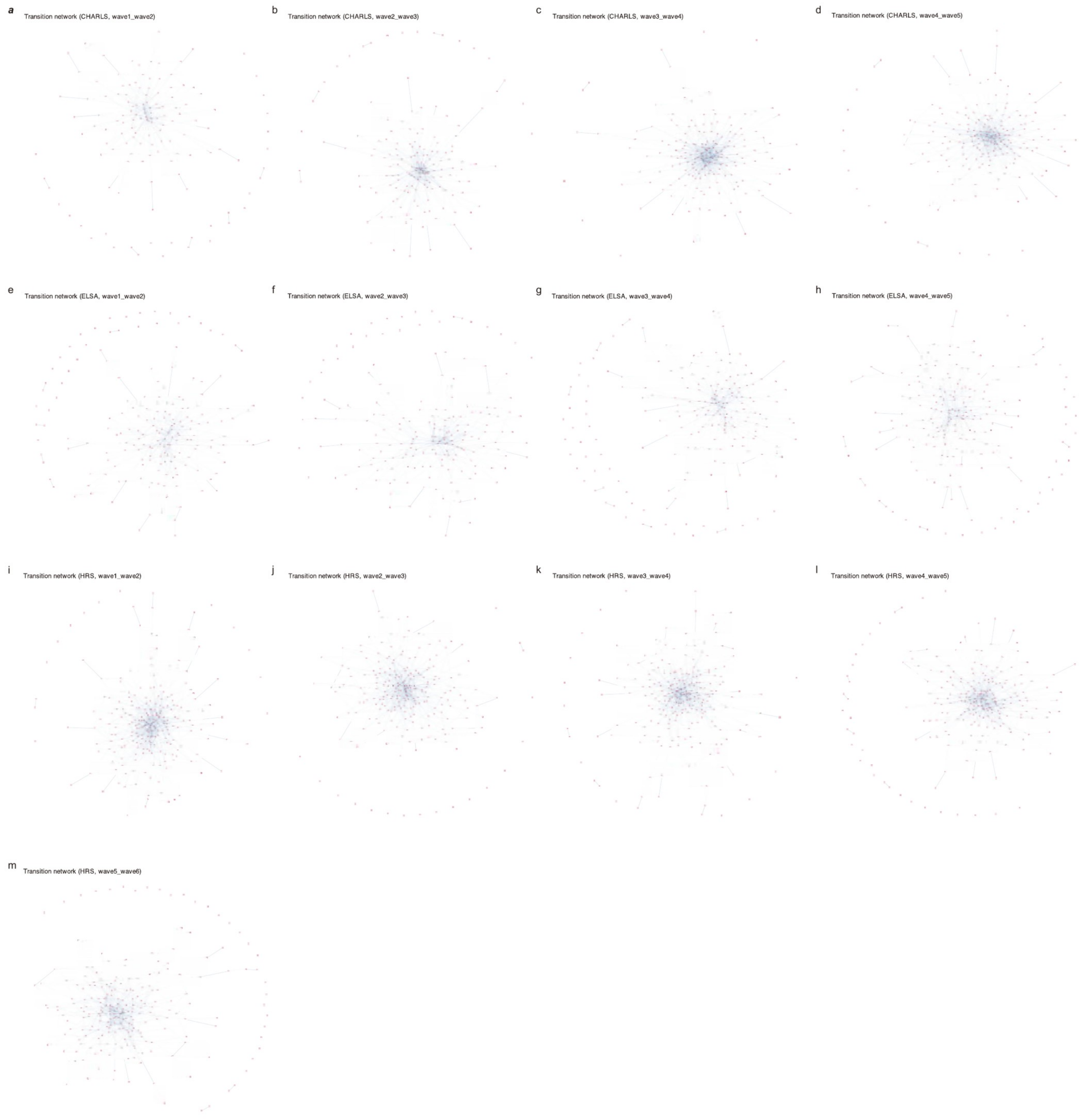

Figure S1. State-transition network visualizations across cohorts and survey windows. Directed weighted networks of multimorbidity state transitions are shown for each cohort and wave interval. Nodes represent observed disease combinations; edges represent transitions between adjacent survey waves; edge thickness reflects the number of individuals undergoing that transition. Across cohorts and windows, networks were sparse, with central clusters of frequently visited states and peripheral states with limited connectivity.

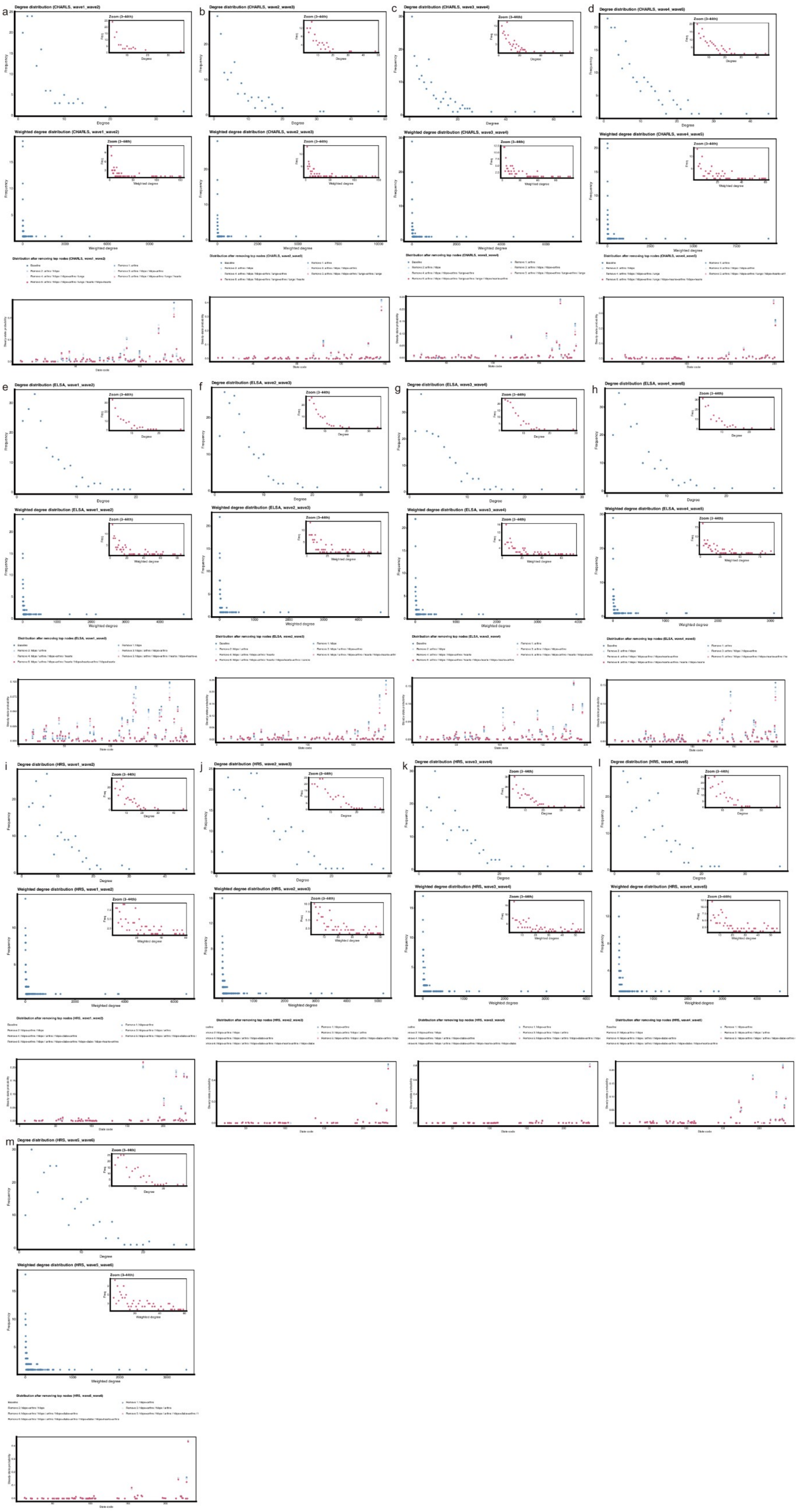

Figure S2. Degree and weighted-degree distributions. Distributions of state connectivity and transition volume are shown for each cohort and wave interval. Insets display log-scaled views. Both degree and weighted-degree distributions were strongly right-skewed, indicating that most states had few outgoing connections and low transition volume, whereas a small number of highly connected states dominated overall transition flow.

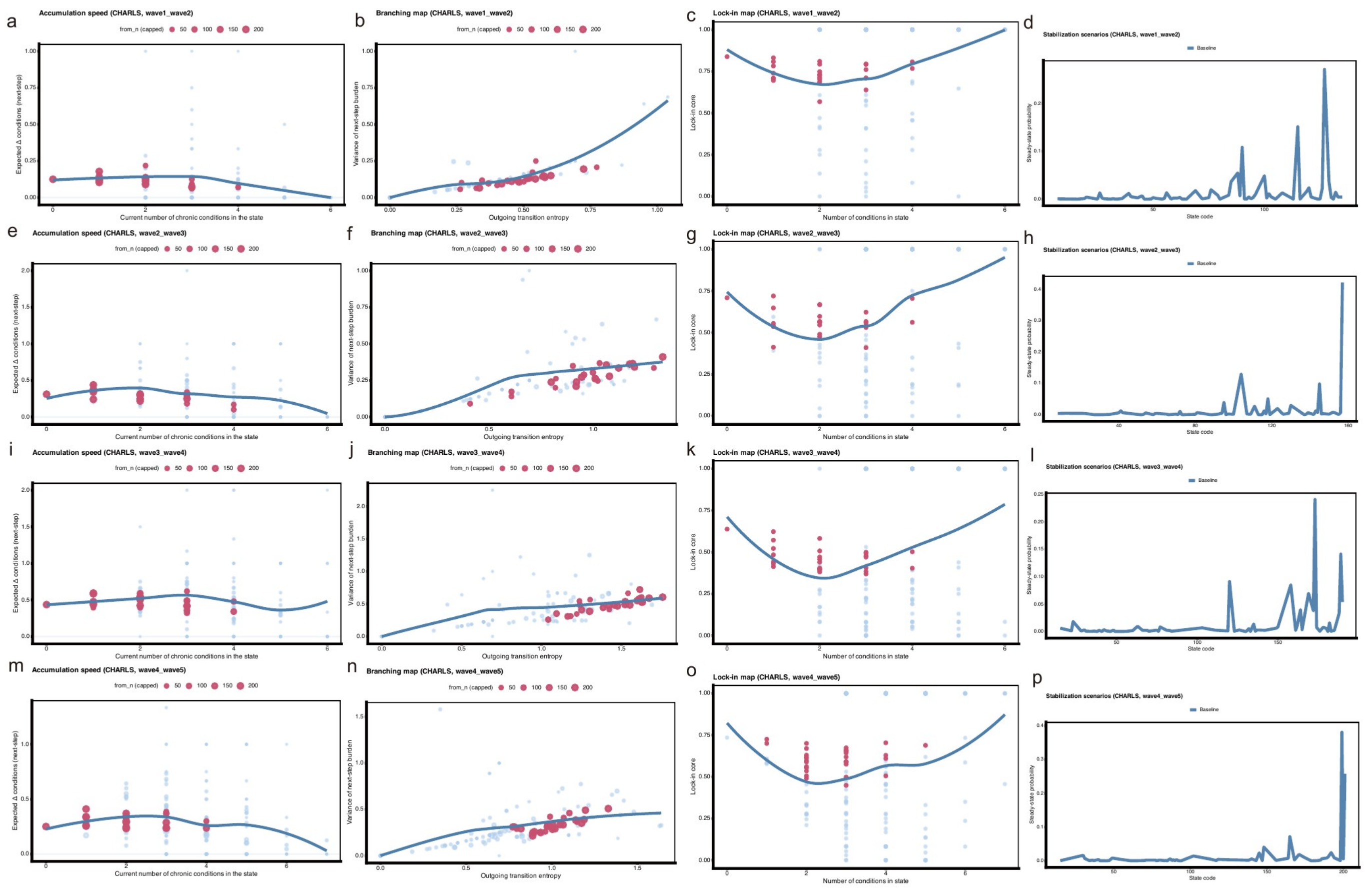

Figure S3A. State-level dynamic signatures in CHARLS. State-level dynamic measures are shown across transition windows in CHARLS, including accumulation speed by disease count, branching maps, lock-in scores, and stabilisation simulations.

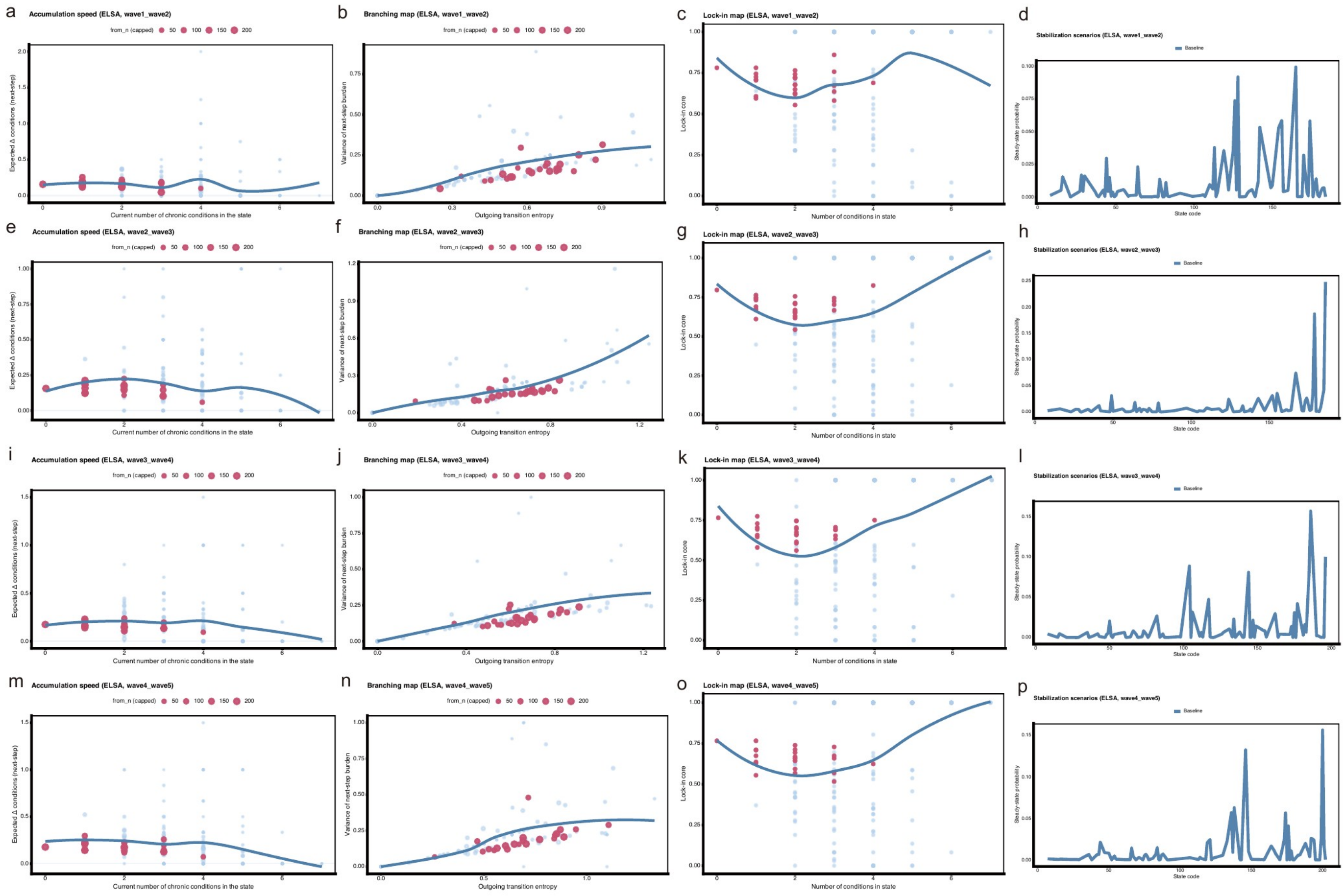

Figure S3B. State-level dynamic signatures in ELSA. State-level dynamic measures are shown across transition windows in ELSA, including accumulation speed, branching patterns, lock-in structure, and stabilisation simulations.

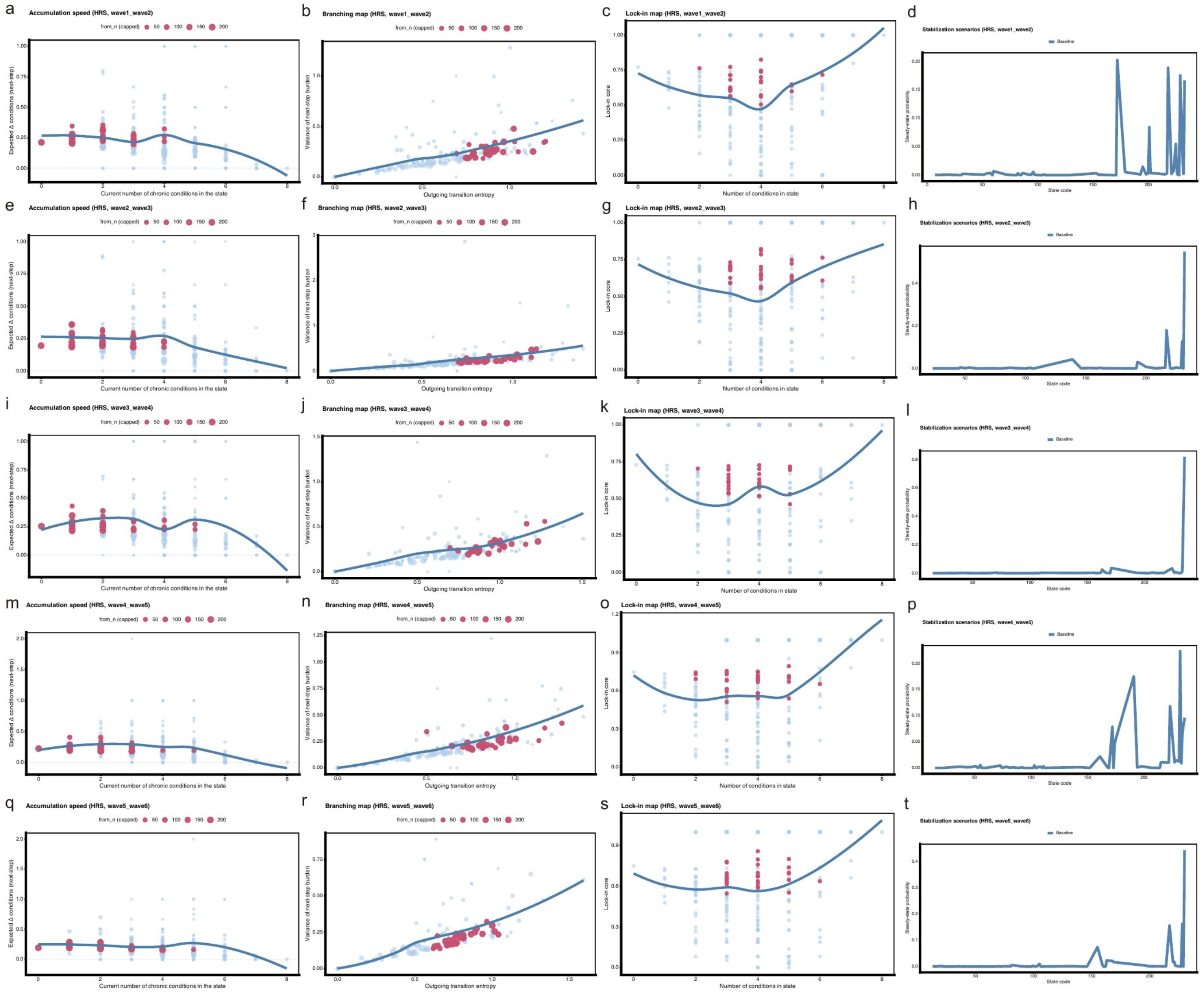

Figure S3C. State-level dynamic signatures in HRS. State-level dynamic measures are shown across transition windows in HRS, including accumulation speed, branching patterns, lock-in structure, and stabilisation simulations.

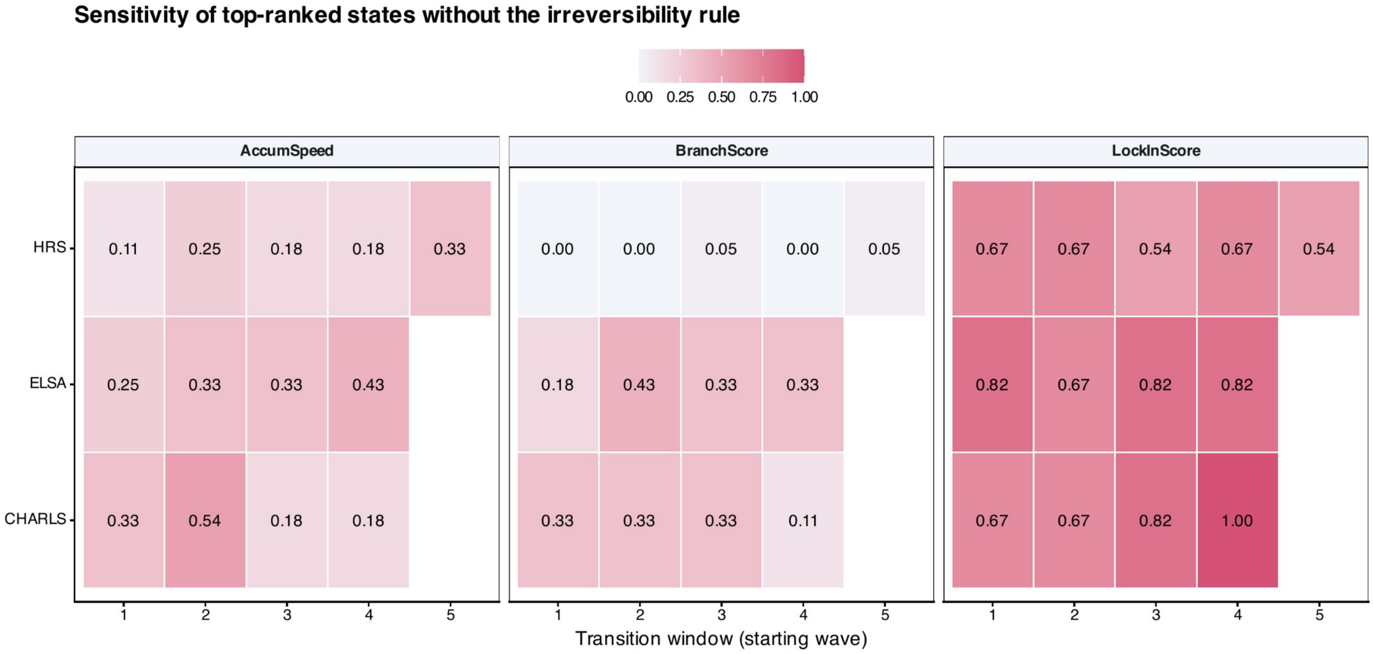

Figure S4. Sensitivity of top-ranked states without the irreversibility rule. Heatmap cells show the top-10 Jaccard overlap between the primary irreversible analysis and the sensitivity analysis without carrying diagnoses forward, stratified by cohort, transition window, and trajectory metric. Darker shading indicates greater overlap; blank cells indicate unavailable transition windows for that cohort.

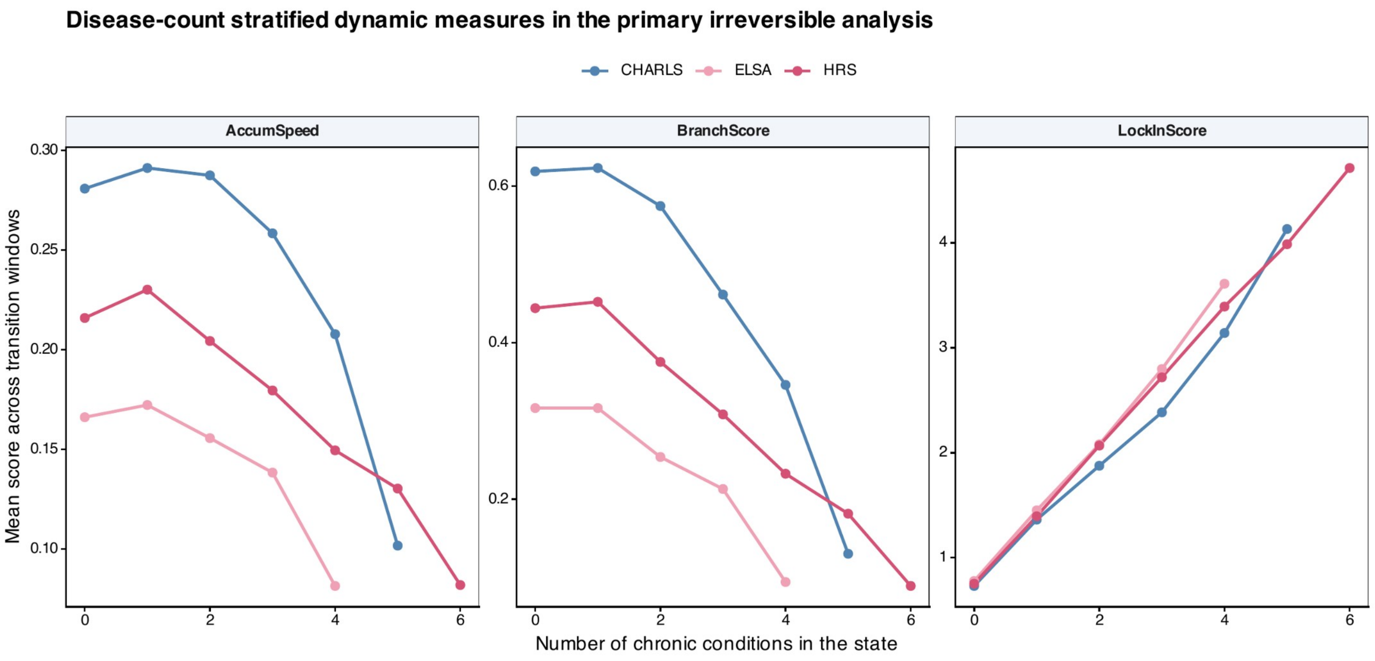

Figure S5. Disease-count stratified dynamic measures in the primary irreversible analysis. Lines show mean state-level scores across transition windows by the number of chronic conditions in each state, separately for accumulation speed, BranchScore, and LockInScore. The figure illustrates how dynamic measures vary with disease burden within each cohort.

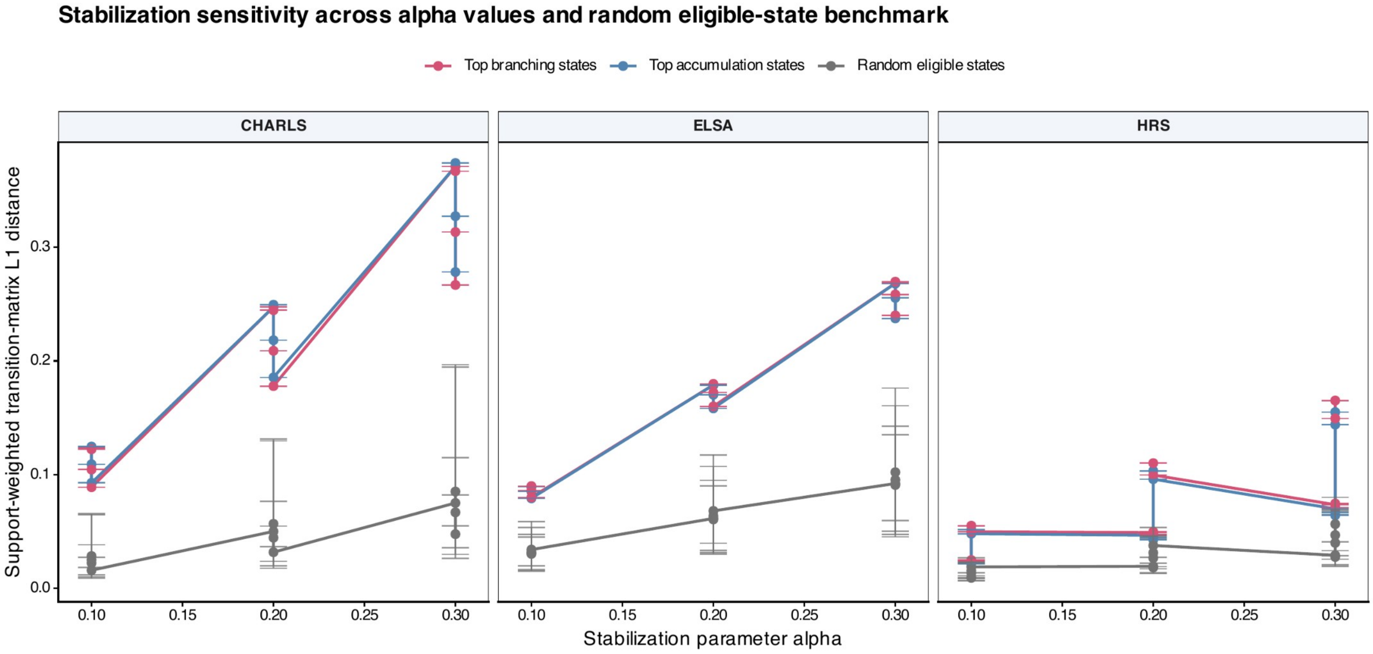

Figure S6. Stabilization alpha sensitivity and random eligible-state benchmark. Support-weighted transition-matrix L1 distances are shown across stabilization parameter alpha values for top branching states, top accumulation states, and randomly selected eligible states, stratified by cohort. Larger values indicate greater perturbation of the transition matrix under the stabilization scenario.

Supplementary Tables

Supplementary Note. Raw state labels are retained from the source tables to avoid changing analytical output. Label components include hibpe (hypertension), diabe (diabetes), cancre (cancer), lunge (chronic lung disease), hearte (heart disease), stroke (stroke), psyche (psychiatric or emotional disorders), and arthre (arthritis or rheumatism). CHARLS, China Health and Retirement Longitudinal Study; ELSA, English Longitudinal Study of Ageing; HRS, Health and Retirement Study.

All supplementary tables are grouped here after the supplementary figures. Tables are formatted as editable Word tables.

Table S1A. States with the highest accumulation speed across cohorts and survey windows.

cohort	window	rank	state label	disease count	AccumSpeed	from n	OutEntropy	out degree	state id
CHARLS	wave1_wave2	1	hearte	1	0.178	331	0.725	12	00001000
CHARLS	wave1_wave2	2	diabe	1	0.141	206	0.602	10	01000000
CHARLS	wave1_wave2	3	hearte+arthre	2	0.133	263	0.58	10	00001001
CHARLS	wave1_wave2	4	lunge+arthre	2	0.127	300	0.514	6	00010001
CHARLS	wave1_wave2	5	Healthy	0	0.125	6,037	0.575	35	00000000
CHARLS	wave1_wave2	6	lunge	1	0.119	481	0.522	11	00010000
CHARLS	wave1_wave2	7	hibpe	1	0.115	1,351	0.544	17	10000000
CHARLS	wave1_wave2	8	hibpe+hearte	2	0.112	303	0.483	6	10001000
CHARLS	wave1_wave2	9	hibpe+lunge	2	0.109	119	0.44	5	10010000
CHARLS	wave1_wave2	10	arthre	1	0.103	2,389	0.478	17	00000001
CHARLS	wave2_wave3	1	lunge	1	0.439	462	1.334	18	00010000
CHARLS	wave2_wave3	2	diabe	1	0.374	214	1.19	13	01000000
CHARLS	wave2_wave3	3	hearte	1	0.367	324	1.175	14	00001000
CHARLS	wave2_wave3	4	hibpe	1	0.34	1,399	1.198	30	10000000
CHARLS	wave2_wave3	5	diabe+arthre	2	0.312	112	0.999	9	01000001
CHARLS	wave2_wave3	6	hibpe+hearte	2	0.312	314	1.078	12	10001000
CHARLS	wave2_wave3	7	Healthy	0	0.311	5,739	1.122	47	00000000
CHARLS	wave2_wave3	8	hibpe+diabe	2	0.308	182	1.063	14	11000000
CHARLS	wave2_wave3	9	hibpe+lunge	2	0.305	131	0.955	9	10010000
CHARLS	wave2_wave3	10	hearte+arthre	2	0.293	290	1.009	10	00001001
CHARLS	wave3_wave4	1	diabe	1	0.594	207	1.673	20	01000000
CHARLS	wave3_wave4	2	diabe+arthre	2	0.589	146	1.615	15	01000001
CHARLS	wave3_wave4	3	hearte	1	0.584	310	1.758	22	00001000
CHARLS	wave3_wave4	4	hibpe+lunge	2	0.547	137	1.633	15	10010000
CHARLS	wave3_wave4	5	hearte+arthre	2	0.545	323	1.611	21	00001001
CHARLS	wave3_wave4	6	hibpe+hearte	2	0.537	322	1.627	20	10001000
CHARLS	wave3_wave4	7	hibpe+diabe	2	0.513	189	1.553	16	11000000
CHARLS	wave3_wave4	8	lunge+arthre	2	0.502	414	1.524	22	00010001
CHARLS	wave3_wave4	9	hibpe+diabe+arthre	3	0.49	145	1.385	9	11000001
CHARLS	wave3_wave4	10	hibpe	1	0.477	1,261	1.599	42	10000000
CHARLS	wave4_wave5	1	hearte	1	0.41	390	1.33	24	00001000
CHARLS	wave4_wave5	2	hibpe+stroke	2	0.371	186	1.197	15	10000100
CHARLS	wave4_wave5	3	hibpe+lunge+hearte	3	0.368	114	1.103	9	10011000
CHARLS	wave4_wave5	4	hibpe+lunge	2	0.343	213	1.14	16	10010000
CHARLS	wave4_wave5	5	lunge	1	0.341	484	1.189	21	00010000
CHARLS	wave4_wave5	6	diabe	1	0.335	328	1.174	17	01000000
CHARLS	wave4_wave5	7	hibpe+diabe	2	0.3	340	1.067	14	11000000
CHARLS	wave4_wave5	8	hearte+arthre	2	0.293	324	1.049	15	00001001
CHARLS	wave4_wave5	9	lunge+arthre	2	0.29	397	1.006	14	00010001
CHARLS	wave4_wave5	10	hibpe+lunge+arthre	3	0.287	258	0.977	13	10010001
ELSA	wave1_wave2	1	diabe	1	0.256	129	0.904	10	01000000
ELSA	wave1_wave2	2	hibpe+psyche	2	0.218	119	0.876	10	10000010
ELSA	wave1_wave2	3	hearte	1	0.201	264	0.808	16	00001000
ELSA	wave1_wave2	4	hibpe+diabe	2	0.183	175	0.727	7	11000000
ELSA	wave1_wave2	5	hibpe+diabe+arthre	3	0.176	148	0.682	8	11000001
ELSA	wave1_wave2	6	psyche	1	0.175	206	0.733	12	00000010
ELSA	wave1_wave2	7	Healthy	0	0.157	2,525	0.72	27	00000000
ELSA	wave1_wave2	8	hibpe	1	0.154	1,108	0.691	15	10000000
ELSA	wave1_wave2	9	hearte+arthre	2	0.137	182	0.609	9	00001001
ELSA	wave1_wave2	10	arthre	1	0.134	935	0.619	17	00000001
ELSA	wave2_wave3	1	psyche	1	0.213	207	0.84	15	00000010
ELSA	wave2_wave3	2	arthre	1	0.184	910	0.79	19	00000001
ELSA	wave2_wave3	3	diabe	1	0.179	117	0.718	9	01000000
ELSA	wave2_wave3	4	hibpe+hearte	2	0.179	229	0.764	10	10001000
ELSA	wave2_wave3	5	hibpe+diabe	2	0.175	166	0.687	6	11000000
ELSA	wave2_wave3	6	hearte+arthre	2	0.173	179	0.729	8	00001001
ELSA	wave2_wave3	7	psyche+arthre	2	0.159	107	0.666	7	00000011
ELSA	wave2_wave3	8	hibpe	1	0.158	1,017	0.697	16	10000000
ELSA	wave2_wave3	9	Healthy	0	0.156	2,489	0.713	33	00000000
ELSA	wave2_wave3	10	hibpe+psyche	2	0.154	104	0.595	5	10000010
ELSA	wave3_wave4	1	hibpe+psyche	2	0.238	101	0.855	7	10000010
ELSA	wave3_wave4	2	cancre	1	0.23	196	0.91	13	00100000
ELSA	wave3_wave4	3	hibpe	1	0.202	878	0.825	21	10000000
ELSA	wave3_wave4	4	hibpe+diabe+arthre	3	0.194	124	0.599	7	11000001
ELSA	wave3_wave4	5	psyche+arthre	2	0.179	112	0.69	6	00000011
ELSA	wave3_wave4	6	Healthy	0	0.175	2,142	0.782	28	00000000
ELSA	wave3_wave4	7	hearte	1	0.166	253	0.708	10	00001000
ELSA	wave3_wave4	8	psyche	1	0.16	194	0.681	10	00000010
ELSA	wave3_wave4	9	diabe	1	0.153	111	0.667	7	01000000
ELSA	wave3_wave4	10	hibpe+arthre	2	0.15	567	0.63	12	10000001

cohort	window	rank	state label	disease count	AccumSpeed	from n	OutEntropy	out degree	state id
ELSA	wave4_wave5	1	psyche	1	0.239	159	0.872	11	00000010
ELSA	wave4_wave5	2	hearte	1	0.221	231	0.889	11	00001000
ELSA	wave4_wave5	3	hibpe	1	0.208	725	0.854	19	10000000
ELSA	wave4_wave5	4	Healthy	0	0.177	1,664	0.771	27	00000000
ELSA	wave4_wave5	5	hibpe+hearte	2	0.175	211	0.69	11	10001000
ELSA	wave4_wave5	6	hearte+arthre	2	0.174	161	0.69	8	00001001
ELSA	wave4_wave5	7	hibpe+arthre	2	0.172	517	0.705	10	10000001
ELSA	wave4_wave5	8	arthre	1	0.143	706	0.648	16	00000001
ELSA	wave4_wave5	9	cancre	1	0.143	175	0.605	8	00100000
ELSA	wave4_wave5	10	hibpe+diabe+arthre	3	0.142	113	0.524	5	11000001
HRS	wave1_wave2	1	lunge+arthre	2	0.324	139	1.023	12	00010001
HRS	wave1_wave2	2	hibpe+psyche	2	0.31	213	1.133	11	10000010
HRS	wave1_wave2	3	psyche	1	0.281	317	0.966	15	00000010
HRS	wave1_wave2	4	hibpe+lunge+arthre	3	0.266	158	0.963	9	10010001
HRS	wave1_wave2	5	diabe	1	0.258	322	0.864	13	01000000
HRS	wave1_wave2	6	diabe+arthre	2	0.246	183	0.91	11	01000001
HRS	wave1_wave2	7	hibpe+hearte	2	0.242	318	0.925	17	10001000
HRS	wave1_wave2	8	hearte	1	0.241	249	0.9	10	00001000
HRS	wave1_wave2	9	hibpe+diabe+hearte	3	0.237	160	0.871	9	11001000
HRS	wave1_wave2	10	hibpe+cancre	2	0.224	174	0.864	11	10100000
HRS	wave2_wave3	1	diabe	1	0.359	273	1.103	15	01000000
HRS	wave2_wave3	2	hearte	1	0.292	209	1.024	16	00001000
HRS	wave2_wave3	3	hibpe+lunge+arthre	3	0.266	158	0.937	7	10010001
HRS	wave2_wave3	4	hibpe+hearte	2	0.265	268	1.009	12	10001000
HRS	wave2_wave3	5	psyche	1	0.263	266	0.955	13	00000010
HRS	wave2_wave3	6	lunge+arthre	2	0.257	113	0.87	9	00010001
HRS	wave2_wave3	7	hibpe+hearte+psyche+arthre	4	0.227	163	0.845	7	10001011
HRS	wave2_wave3	8	hibpe+diabe	2	0.225	582	0.857	15	11000000
HRS	wave2_wave3	9	hibpe	1	0.222	1,691	0.91	25	10000000
HRS	wave2_wave3	10	hibpe+diabe+hearte	3	0.211	142	0.746	10	11001000
HRS	wave3_wave4	1	psyche	1	0.348	221	1.232	17	00000010
HRS	wave3_wave4	2	hibpe+stroke+arthre	3	0.304	125	1.072	10	10000101
HRS	wave3_wave4	3	diabe	1	0.298	215	0.969	10	01000000
HRS	wave3_wave4	4	hibpe+psyche	2	0.284	176	0.947	11	10000010
HRS	wave3_wave4	5	hearte	1	0.272	169	1.016	14	00001000
HRS	wave3_wave4	6	hibpe+hearte	2	0.27	241	0.973	16	10001000
HRS	wave3_wave4	7	hibpe	1	0.261	1,381	1.026	30	10000000
HRS	wave3_wave4	8	Healthy	0	0.255	2,195	1	40	00000000
HRS	wave3_wave4	9	hibpe+cancre	2	0.252	163	0.932	7	10100000
HRS	wave3_wave4	10	diabe+arthre	2	0.246	175	0.857	9	01000001
HRS	wave4_wave5	1	psyche	1	0.286	280	0.949	13	00000010
HRS	wave4_wave5	2	hibpe+hearte	2	0.273	238	1.004	14	10001000
HRS	wave4_wave5	3	hibpe+lunge+arthre	3	0.265	136	0.972	9	10010001
HRS	wave4_wave5	4	hibpe+psyche	2	0.263	198	0.926	12	10000010
HRS	wave4_wave5	5	cancre	1	0.262	206	0.926	9	00100000
HRS	wave4_wave5	6	hibpe+diabe+hearte	3	0.25	140	0.921	11	11001000
HRS	wave4_wave5	7	diabe+arthre	2	0.247	190	0.908	12	01000001
HRS	wave4_wave5	8	hibpe	1	0.235	1,459	0.923	22	10000000
HRS	wave4_wave5	9	hibpe+cancre	2	0.231	156	0.843	8	10100000
HRS	wave4_wave5	10	Healthy	0	0.223	2,569	0.909	37	00000000
HRS	wave5_wave6	1	diabe	1	0.286	241	1.001	12	01000000
HRS	wave5_wave6	2	hibpe+hearte	2	0.284	194	1.016	9	10001000
HRS	wave5_wave6	3	hearte	1	0.282	131	1.041	9	00001000
HRS	wave5_wave6	4	cancre	1	0.257	171	0.938	11	00100000
HRS	wave5_wave6	5	hibpe+psyche	2	0.248	161	0.901	12	10000010
HRS	wave5_wave6	6	diabe+arthre	2	0.241	174	0.814	9	01000001
HRS	wave5_wave6	7	hibpe	1	0.223	1,168	0.889	23	10000000
HRS	wave5_wave6	8	psyche	1	0.222	221	0.863	13	00000010
HRS	wave5_wave6	9	hibpe+lunge+arthre	3	0.21	124	0.763	9	10010001
HRS	wave5_wave6	10	cancre+arthre	2	0.206	165	0.793	11	00100001

Table S1B. Accumulation speed summarized by baseline disease count across cohorts and survey windows.

cohort	window	disease count	n states	mean accum	median accum	p25 accum	p75 accum
CHARLS	wave1_wave2	0	1	0.125	0.125	0.125	0.125
CHARLS	wave1_wave2	1	7	0.135	0.133	0.117	0.149
CHARLS	wave1_wave2	2	9	0.126	0.112	0.105	0.133
CHARLS	wave1_wave2	3	5	0.086	0.083	0.071	0.092
CHARLS	wave1_wave2	4	2	0.07	0.07	0.07	0.071
CHARLS	wave2_wave3	0	1	0.311	0.311	0.311	0.311
CHARLS	wave2_wave3	1	6	0.363	0.371	0.347	0.405
CHARLS	wave2_wave3	2	9	0.286	0.305	0.255	0.312
CHARLS	wave2_wave3	3	6	0.272	0.283	0.256	0.293
CHARLS	wave2_wave3	4	2	0.135	0.135	0.118	0.152
CHARLS	wave3_wave4	0	1	0.436	0.436	0.436	0.436
CHARLS	wave3_wave4	1	7	0.482	0.458	0.434	0.53
CHARLS	wave3_wave4	2	10	0.512	0.525	0.494	0.547

cohort	window	disease count	n states	mean accum	median accum	p25 accum	p75 accum
CHARLS	wave3_wave4	3	7	0.442	0.417	0.387	0.482
CHARLS	wave3_wave4	4	2	0.41	0.41	0.376	0.444
CHARLS	wave4_wave5	0	1	0.251	0.251	0.251	0.251
CHARLS	wave4_wave5	1	8	0.289	0.272	0.255	0.337
CHARLS	wave4_wave5	2	13	0.304	0.3	0.286	0.343
CHARLS	wave4_wave5	3	10	0.267	0.239	0.231	0.278
CHARLS	wave4_wave5	4	4	0.235	0.233	0.215	0.253
CHARLS	wave4_wave5	5	1	0.102	0.102	0.102	0.102
ELSA	wave1_wave2	0	1	0.157	0.157	0.157	0.157
ELSA	wave1_wave2	1	7	0.175	0.175	0.144	0.193
ELSA	wave1_wave2	2	10	0.152	0.149	0.119	0.178
ELSA	wave1_wave2	3	5	0.134	0.169	0.091	0.176
ELSA	wave1_wave2	4	1	0.1	0.1	0.1	0.1
ELSA	wave2_wave3	0	1	0.156	0.156	0.156	0.156
ELSA	wave2_wave3	1	7	0.168	0.179	0.142	0.189
ELSA	wave2_wave3	2	9	0.163	0.159	0.151	0.175
ELSA	wave2_wave3	3	4	0.135	0.128	0.108	0.155
ELSA	wave2_wave3	4	1	0.059	0.059	0.059	0.059
ELSA	wave3_wave4	0	1	0.175	0.175	0.175	0.175
ELSA	wave3_wave4	1	7	0.18	0.166	0.156	0.205
ELSA	wave3_wave4	2	10	0.155	0.148	0.134	0.175
ELSA	wave3_wave4	3	5	0.147	0.135	0.131	0.145
ELSA	wave3_wave4	4	1	0.094	0.094	0.094	0.094
ELSA	wave4_wave5	0	1	0.177	0.177	0.177	0.177
ELSA	wave4_wave5	1	7	0.213	0.221	0.176	0.239
ELSA	wave4_wave5	2	8	0.17	0.173	0.139	0.186
ELSA	wave4_wave5	3	5	0.17	0.142	0.132	0.192
ELSA	wave4_wave5	4	1	0.073	0.073	0.073	0.073
HRS	wave1_wave2	0	1	0.212	0.212	0.212	0.212
HRS	wave1_wave2	1	8	0.25	0.238	0.217	0.264
HRS	wave1_wave2	2	13	0.244	0.242	0.177	0.31
HRS	wave1_wave2	3	16	0.204	0.197	0.185	0.226
HRS	wave1_wave2	4	11	0.159	0.137	0.117	0.188
HRS	wave1_wave2	5	4	0.15	0.146	0.137	0.16
HRS	wave1_wave2	6	1	0.075	0.075	0.075	0.075
HRS	wave2_wave3	0	1	0.195	0.195	0.195	0.195
HRS	wave2_wave3	1	7	0.268	0.263	0.21	0.325
HRS	wave2_wave3	2	13	0.217	0.197	0.181	0.257
HRS	wave2_wave3	3	15	0.197	0.186	0.158	0.23
HRS	wave2_wave3	4	12	0.157	0.166	0.129	0.192
HRS	wave2_wave3	5	6	0.125	0.12	0.111	0.126
HRS	wave2_wave3	6	2	0.086	0.086	0.072	0.1
HRS	wave3_wave4	0	1	0.255	0.255	0.255	0.255
HRS	wave3_wave4	1	7	0.294	0.272	0.247	0.323
HRS	wave3_wave4	2	11	0.256	0.246	0.214	0.277
HRS	wave3_wave4	3	15	0.214	0.199	0.174	0.233
HRS	wave3_wave4	4	11	0.2	0.188	0.172	0.233
HRS	wave3_wave4	5	5	0.169	0.14	0.119	0.226
HRS	wave4_wave5	0	1	0.223	0.223	0.223	0.223
HRS	wave4_wave5	1	7	0.259	0.235	0.217	0.274
HRS	wave4_wave5	2	12	0.239	0.219	0.185	0.265
HRS	wave4_wave5	3	12	0.216	0.225	0.175	0.259
HRS	wave4_wave5	4	11	0.139	0.139	0.121	0.159
HRS	wave4_wave5	5	5	0.114	0.113	0.087	0.123
HRS	wave4_wave5	6	1	0.097	0.097	0.097	0.097
HRS	wave5_wave6	0	1	0.194	0.194	0.194	0.194
HRS	wave5_wave6	1	6	0.243	0.24	0.222	0.276
HRS	wave5_wave6	2	11	0.218	0.206	0.184	0.245
HRS	wave5_wave6	3	12	0.164	0.173	0.146	0.183
HRS	wave5_wave6	4	11	0.13	0.14	0.099	0.162
HRS	wave5_wave6	5	6	0.109	0.117	0.081	0.121
HRS	wave5_wave6	6	1	0.069	0.069	0.069	0.069

Table S2A. Top branching multimorbidity states by cohort and survey window.

cohort	window	rank	state label	disease count	BranchScore	OutEntropy	Var dc to	from n	out degree	state id
CHARLS	wave1_wave2	1	hearte	1	0.32	0.725	0.195	331	12	00001000
CHARLS	wave1_wave2	2	diabe	1	0.233	0.602	0.15	206	10	01000000
CHARLS	wave1_wave2	3	Healthy	0	0.221	0.575	0.148	6,037	35	00000000
CHARLS	wave1_wave2	4	hearte+arthre	2	0.216	0.58	0.138	263	10	00001001
CHARLS	wave1_wave2	5	hibpe	1	0.194	0.544	0.127	1,351	17	10000000
CHARLS	wave1_wave2	6	lunge	1	0.191	0.522	0.134	481	11	00010000
CHARLS	wave1_wave2	7	lunge+arthre	2	0.186	0.514	0.131	300	6	00010001
CHARLS	wave1_wave2	8	arthre	1	0.162	0.478	0.115	2,389	17	00000001
CHARLS	wave1_wave2	9	hibpe+hearte	2	0.157	0.483	0.106	303	6	10001000
CHARLS	wave1_wave2	10	hibpe+diabe	2	0.151	0.455	0.111	175	9	11000000

cohort	window	rank	state label	disease count	BranchScore	OutEntropy	Var dc to	from n	out degree	state id
CHARLS	wave2_wave3	1	lunge	1	0.855	1.334	0.411	462	18	00010000
CHARLS	wave2_wave3	2	diabe	1	0.719	1.19	0.365	214	13	01000000
CHARLS	wave2_wave3	3	hearte	1	0.707	1.175	0.362	324	14	00001000
CHARLS	wave2_wave3	4	hibpe	1	0.7	1.198	0.342	1,399	30	10000000
CHARLS	wave2_wave3	5	Healthy	0	0.649	1.122	0.335	5,739	47	00000000
CHARLS	wave2_wave3	6	hibpe+diabe	2	0.634	1.063	0.356	182	14	11000000
CHARLS	wave2_wave3	7	hibpe+hearte	2	0.569	1.078	0.278	314	12	10001000
CHARLS	wave2_wave3	8	diabe+arthre	2	0.551	0.999	0.304	112	9	01000001
CHARLS	wave2_wave3	9	hibpe+lunge+arthre	3	0.518	1.012	0.262	107	9	10010001
CHARLS	wave2_wave3	10	hearte+arthre	2	0.517	1.009	0.262	290	10	00001001
CHARLS	wave3_wave4	1	diabe+arthre	2	1.372	1.615	0.722	146	15	01000001
CHARLS	wave3_wave4	2	hearte	1	1.366	1.758	0.604	310	22	00001000
CHARLS	wave3_wave4	3	diabe	1	1.284	1.673	0.589	207	20	01000000
CHARLS	wave3_wave4	4	hearte+arthre	2	1.223	1.611	0.576	323	21	00001001
CHARLS	wave3_wave4	5	hibpe	1	1.187	1.599	0.551	1,261	42	10000000
CHARLS	wave3_wave4	6	hibpe+lunge	2	1.183	1.633	0.525	137	15	10010000
CHARLS	wave3_wave4	7	hibpe+hearte	2	1.182	1.627	0.528	322	20	10001000
CHARLS	wave3_wave4	8	lunge+arthre	2	1.114	1.524	0.535	414	22	00010001
CHARLS	wave3_wave4	9	hibpe+diabe	2	1.079	1.553	0.483	189	16	11000000
CHARLS	wave3_wave4	10	Healthy	0	1.067	1.523	0.491	4,337	67	00000000
CHARLS	wave4_wave5	1	hearte	1	0.949	1.33	0.509	390	24	00001000
CHARLS	wave4_wave5	2	hibpe+stroke	2	0.839	1.197	0.491	186	15	10000100
CHARLS	wave4_wave5	3	hibpe+lunge+hearte	3	0.749	1.103	0.461	114	9	10011000
CHARLS	wave4_wave5	4	lunge	1	0.742	1.189	0.39	484	21	00010000
CHARLS	wave4_wave5	5	hibpe+lunge	2	0.707	1.14	0.385	213	16	10010000
CHARLS	wave4_wave5	6	diabe	1	0.696	1.174	0.351	328	17	01000000
CHARLS	wave4_wave5	7	hibpe+diabe	2	0.616	1.067	0.334	340	14	11000000
CHARLS	wave4_wave5	8	hearte+arthre	2	0.609	1.049	0.337	324	15	00001001
CHARLS	wave4_wave5	9	lunge+arthre	2	0.579	1.006	0.332	397	14	00010001
CHARLS	wave4_wave5	10	hibpe+lunge+arthre	3	0.56	0.977	0.329	258	13	10010001
ELSA	wave1_wave2	1	diabe	1	0.507	0.904	0.314	129	10	01000000
ELSA	wave1_wave2	2	hibpe+psyche	2	0.412	0.876	0.221	119	10	10000010
ELSA	wave1_wave2	3	hearte	1	0.405	0.808	0.251	264	16	00001000
ELSA	wave1_wave2	4	psyche	1	0.322	0.733	0.193	206	12	00000010
ELSA	wave1_wave2	5	hibpe+diabe+arthre	3	0.304	0.682	0.199	148	8	11000001
ELSA	wave1_wave2	6	Healthy	0	0.292	0.72	0.165	2,525	27	00000000
ELSA	wave1_wave2	7	hibpe+diabe	2	0.292	0.727	0.161	175	7	11000000
ELSA	wave1_wave2	8	hibpe	1	0.268	0.691	0.15	1,108	15	10000000
ELSA	wave1_wave2	9	hearte+arthre	2	0.237	0.609	0.151	182	9	00001001
ELSA	wave1_wave2	10	arthre	1	0.233	0.619	0.141	935	17	00000001
ELSA	wave2_wave3	1	psyche	1	0.432	0.84	0.264	207	15	00000010
ELSA	wave2_wave3	2	arthre	1	0.354	0.79	0.2	910	19	00000001
ELSA	wave2_wave3	3	diabe	1	0.32	0.718	0.199	117	9	01000000
ELSA	wave2_wave3	4	hibpe+hearte	2	0.318	0.764	0.173	229	10	10001000
ELSA	wave2_wave3	5	Healthy	0	0.299	0.713	0.176	2,489	33	00000000
ELSA	wave2_wave3	6	hearte+arthre	2	0.297	0.729	0.166	179	8	00001001
ELSA	wave2_wave3	7	hibpe	1	0.288	0.697	0.171	1,017	16	10000000
ELSA	wave2_wave3	8	hibpe+diabe	2	0.271	0.687	0.156	166	6	11000000
ELSA	wave2_wave3	9	psyche+arthre	2	0.26	0.666	0.152	107	7	00000011
ELSA	wave2_wave3	10	hibpe+arthre	2	0.245	0.627	0.153	641	13	10000001
ELSA	wave3_wave4	1	cancre	1	0.444	0.91	0.238	196	13	00100000
ELSA	wave3_wave4	2	hibpe	1	0.385	0.825	0.218	878	21	10000000
ELSA	wave3_wave4	3	hibpe+psyche	2	0.383	0.855	0.201	101	7	10000010
ELSA	wave3_wave4	4	Healthy	0	0.339	0.782	0.188	2,142	28	00000000
ELSA	wave3_wave4	5	hibpe+diabe+arthre	3	0.301	0.599	0.253	124	7	11000001
ELSA	wave3_wave4	6	psyche+arthre	2	0.295	0.69	0.182	112	6	00000011
ELSA	wave3_wave4	7	hearte	1	0.278	0.708	0.154	253	10	00001000
ELSA	wave3_wave4	8	psyche	1	0.277	0.681	0.165	194	10	00000010
ELSA	wave3_wave4	9	hibpe+arthre	2	0.254	0.63	0.163	567	12	10000001
ELSA	wave3_wave4	10	arthre	1	0.254	0.626	0.164	782	16	00000001
ELSA	wave4_wave5	1	psyche	1	0.442	0.872	0.257	159	11	00000010
ELSA	wave4_wave5	2	hibpe	1	0.408	0.854	0.228	725	19	10000000
ELSA	wave4_wave5	3	hearte	1	0.404	0.889	0.207	231	11	00001000
ELSA	wave4_wave5	4	Healthy	0	0.335	0.771	0.189	1,664	27	00000000
ELSA	wave4_wave5	5	hibpe+hearte	2	0.31	0.69	0.201	211	11	10001000
ELSA	wave4_wave5	6	hearte+arthre	2	0.294	0.69	0.181	161	8	00001001
ELSA	wave4_wave5	7	hibpe+arthre	2	0.28	0.705	0.158	517	10	10000001
ELSA	wave4_wave5	8	arthre	1	0.256	0.648	0.157	706	16	00000001
ELSA	wave4_wave5	9	cancre	1	0.231	0.605	0.145	175	8	00100000
ELSA	wave4_wave5	10	hibpe+diabe	2	0.214	0.614	0.121	142	6	11000000
HRS	wave1_wave2	1	lunge+arthre	2	0.707	1.023	0.478	139	12	00010001
HRS	wave1_wave2	2	psyche	1	0.569	0.966	0.347	317	15	00000010
HRS	wave1_wave2	3	hibpe+psyche	2	0.568	1.133	0.251	213	11	10000010
HRS	wave1_wave2	4	hibpe+hearte	2	0.482	0.925	0.272	318	17	10001000
HRS	wave1_wave2	5	hibpe+lunge+arthre	3	0.477	0.963	0.246	158	9	10010001
HRS	wave1_wave2	6	diabe	1	0.471	0.864	0.297	322	13	01000000
HRS	wave1_wave2	7	hearte	1	0.469	0.9	0.271	249	10	00001000
HRS	wave1_wave2	8	diabe+arthre	2	0.456	0.91	0.251	183	11	01000001
HRS	wave1_wave2	9	Healthy	0	0.45	0.866	0.27	3,544	45	00000000
HRS	wave1_wave2	10	arthre	1	0.43	0.873	0.243	1,687	28	00000001
HRS	wave2_wave3	1	diabe	1	0.752	1.103	0.465	273	15	01000000
HRS	wave2_wave3	2	hearte	1	0.614	1.024	0.36	209	16	00001000

cohort	window	rank	state label	disease count	BranchScore	OutEntropy	Var dc to	from n	out degree	state id
HRS	wave2_wave3	3	hibpe+hearte	2	0.516	1.009	0.262	268	12	10001000
HRS	wave2_wave3	4	lunge+arthre	2	0.502	0.87	0.332	113	9	00010001
HRS	wave2_wave3	5	psyche	1	0.495	0.955	0.269	266	13	00000010
HRS	wave2_wave3	6	hibpe+lunge+arthre	3	0.44	0.937	0.22	158	7	10010001
HRS	wave2_wave3	7	hibpe	1	0.429	0.91	0.222	1,691	25	10000000
HRS	wave2_wave3	8	hibpe+diabe	2	0.41	0.857	0.229	582	15	11000000
HRS	wave2_wave3	9	hibpe+diabe+hearte	3	0.394	0.746	0.279	142	10	11001000
HRS	wave2_wave3	10	psyche+arthre	2	0.381	0.796	0.229	361	11	00000011
HRS	wave3_wave4	1	psyche	1	0.714	1.232	0.336	221	17	00000010
HRS	wave3_wave4	2	hibpe+stroke+arthre	3	0.595	1.072	0.308	125	10	10000101
HRS	wave3_wave4	3	diabe	1	0.572	0.969	0.349	215	10	01000000
HRS	wave3_wave4	4	hibpe+hearte	2	0.559	0.973	0.33	241	16	10001000
HRS	wave3_wave4	5	Healthy	0	0.558	1	0.311	2,195	40	00000000
HRS	wave3_wave4	6	hearte	1	0.549	1.016	0.293	169	14	00001000
HRS	wave3_wave4	7	hibpe	1	0.541	1.026	0.278	1,381	30	10000000
HRS	wave3_wave4	8	hibpe+psyche	2	0.533	0.947	0.317	176	11	10000010
HRS	wave3_wave4	9	cancre	1	0.444	0.856	0.269	198	12	00100000
HRS	wave3_wave4	10	diabe+arthre	2	0.441	0.857	0.265	175	9	01000001
HRS	wave4_wave5	1	psyche	1	0.587	0.949	0.383	280	13	00000010
HRS	wave4_wave5	2	hibpe+hearte	2	0.526	1.004	0.274	238	14	10001000
HRS	wave4_wave5	3	hibpe+psyche	2	0.494	0.926	0.285	198	12	10000010
HRS	wave4_wave5	4	cancre	1	0.49	0.926	0.281	206	9	00100000
HRS	wave4_wave5	5	diabe+arthre	2	0.49	0.908	0.291	190	12	01000001
HRS	wave4_wave5	6	hibpe+lunge+arthre	3	0.489	0.972	0.253	136	9	10010001
HRS	wave4_wave5	7	hibpe+diabe+hearte	3	0.469	0.921	0.259	140	11	11001000
HRS	wave4_wave5	8	Healthy	0	0.463	0.909	0.26	2,569	37	00000000
HRS	wave4_wave5	9	hibpe	1	0.451	0.923	0.239	1,459	22	10000000
HRS	wave4_wave5	10	hearte	1	0.406	0.818	0.246	157	12	00001000
HRS	wave5_wave6	1	diabe	1	0.544	1.001	0.296	241	12	01000000
HRS	wave5_wave6	2	hibpe+hearte	2	0.513	1.016	0.255	194	9	10001000
HRS	wave5_wave6	3	hearte	1	0.503	1.041	0.233	131	9	00001000
HRS	wave5_wave6	4	hibpe+psyche	2	0.471	0.901	0.274	161	12	10000010
HRS	wave5_wave6	5	cancre	1	0.457	0.938	0.238	171	11	00100000
HRS	wave5_wave6	6	hibpe	1	0.445	0.889	0.25	1,168	23	10000000
HRS	wave5_wave6	7	psyche	1	0.419	0.863	0.236	221	13	00000010
HRS	wave5_wave6	8	diabe+arthre	2	0.39	0.814	0.229	174	9	01000001
HRS	wave5_wave6	9	cancre+arthre	2	0.386	0.793	0.236	165	11	00100001
HRS	wave5_wave6	10	Healthy	0	0.372	0.814	0.209	1,863	26	00000000

Table S2B. Top lock-in multimorbidity states by cohort and survey window.

cohort	window	rank	state label	disease count	LockInScore	LockInCore	EntropyNorm	from n	out degree	state id
CHARLS	wave1_wave2	1	hibpe+hearte+arthre	3	3.168	0.792	0.208	240	5	10001001
CHARLS	wave1_wave2	2	hibpe+arthre	2	2.43	0.81	0.19	755	9	10000001
CHARLS	wave1_wave2	3	hibpe+diabe	2	2.379	0.793	0.207	175	9	11000000
CHARLS	wave1_wave2	4	hearte+arthre	2	2.245	0.748	0.252	263	10	00001001
CHARLS	wave1_wave2	5	hibpe+hearte	2	2.191	0.73	0.27	303	6	10001000
CHARLS	wave1_wave2	6	hibpe+lunge	2	2.18	0.727	0.273	119	5	10010000
CHARLS	wave1_wave2	7	lunge+arthre	2	2.14	0.713	0.287	300	6	00010001
CHARLS	wave1_wave2	8	arthre	1	1.662	0.831	0.169	2,389	17	00000001
CHARLS	wave1_wave2	9	hibpe	1	1.616	0.808	0.192	1,351	17	10000000
CHARLS	wave1_wave2	10	lunge	1	1.565	0.782	0.218	481	11	00010000
CHARLS	wave2_wave3	1	hibpe+hearte+arthre	3	2.234	0.559	0.441	261	8	10001001
CHARLS	wave2_wave3	2	hibpe+lunge+arthre	3	2.158	0.54	0.46	107	9	10010001
CHARLS	wave2_wave3	3	hibpe+arthre	2	2.004	0.668	0.332	789	16	10000001
CHARLS	wave2_wave3	4	lunge+arthre	2	2.001	0.667	0.333	294	11	00010001
CHARLS	wave2_wave3	5	hibpe+diabe	2	1.792	0.597	0.403	182	14	11000000
CHARLS	wave2_wave3	6	hibpe+hearte	2	1.699	0.566	0.434	314	12	10001000
CHARLS	wave2_wave3	7	hibpe+lunge	2	1.696	0.565	0.435	131	9	10010000
CHARLS	wave2_wave3	8	hearte+arthre	2	1.685	0.562	0.438	290	10	00001001
CHARLS	wave2_wave3	9	diabe+arthre	2	1.636	0.545	0.455	112	9	01000001
CHARLS	wave2_wave3	10	arthre	1	1.439	0.72	0.28	2,362	29	00000001
CHARLS	wave3_wave4	1	hibpe+lunge+hearte+arthre	4	2.511	0.502	0.498	126	9	10011001
CHARLS	wave3_wave4	2	hibpe+hearte+arthre	3	1.972	0.493	0.507	362	13	10001001
CHARLS	wave3_wave4	3	hibpe+lunge+arthre	3	1.914	0.478	0.522	168	11	10010001
CHARLS	wave3_wave4	4	lunge+hearte+arthre	3	1.882	0.471	0.529	126	9	00011001
CHARLS	wave3_wave4	5	hibpe+arthre	2	1.747	0.582	0.418	947	30	10000001
CHARLS	wave3_wave4	6	lunge+arthre	2	1.521	0.507	0.493	414	22	00010001
CHARLS	wave3_wave4	7	hibpe+diabe+arthre	3	1.478	0.37	0.63	145	9	11000001
CHARLS	wave3_wave4	8	hearte+arthre	2	1.412	0.471	0.529	323	21	00001001
CHARLS	wave3_wave4	9	hibpe+hearte	2	1.371	0.457	0.543	322	20	10001000
CHARLS	wave3_wave4	10	hibpe+diabe	2	1.32	0.44	0.56	189	16	11000000
CHARLS	wave4_wave5	1	hibpe+diabe+hearte+arthre	4	3.519	0.704	0.296	137	9	11001001
CHARLS	wave4_wave5	2	hibpe+lunge+hearte+arthre	4	3.143	0.629	0.371	169	8	10011001
CHARLS	wave4_wave5	3	hibpe+diabe+hearte	3	2.694	0.674	0.326	168	10	11001000
CHARLS	wave4_wave5	4	lunge+hearte+arthre	3	2.643	0.661	0.339	162	10	00011001
CHARLS	wave4_wave5	5	hibpe+hearte+arthre	3	2.614	0.653	0.347	418	13	10001001
CHARLS	wave4_wave5	6	hibpe+diabe+arthre	3	2.575	0.644	0.356	219	10	11000001
CHARLS	wave4_wave5	7	hibpe+lunge+arthre	3	2.476	0.619	0.381	258	13	10010001
CHARLS	wave4_wave5	8	hibpe+stroke+arthre	3	2.302	0.575	0.425	114	7	10000101
CHARLS	wave4_wave5	9	hibpe+arthre	2	2.1	0.7	0.3	1,074	20	10000001

cohort	window	rank	state label	disease count	LockInScore	LockInCore	EntropyNorm	from n	out degree	state id
CHARLS	wave4_wave5	10	hibpe+hearte	2	2.009	0.67	0.33	416	17	10001000
ELSA	wave1_wave2	1	hibpe+hearte+arthre	3	3.44	0.86	0.14	221	6	10001001
ELSA	wave1_wave2	2	hibpe+diabe+arthre	3	2.689	0.672	0.328	148	8	11000001
ELSA	wave1_wave2	3	hibpe+arthre	2	2.295	0.765	0.235	672	10	10000001
ELSA	wave1_wave2	4	hibpe+hearte	2	2.225	0.742	0.258	224	7	10001000
ELSA	wave1_wave2	5	hearte+arthre	2	2.169	0.723	0.277	182	9	00001001
ELSA	wave1_wave2	6	hibpe+cancre	2	2.031	0.677	0.323	101	5	10100000
ELSA	wave1_wave2	7	hibpe+diabe	2	1.879	0.626	0.374	175	7	11000000
ELSA	wave1_wave2	8	hibpe+psyche	2	1.859	0.62	0.38	119	10	10000010
ELSA	wave1_wave2	9	arthre	1	1.563	0.782	0.218	935	17	00000001
ELSA	wave1_wave2	10	hibpe	1	1.489	0.745	0.255	1,108	15	10000000
ELSA	wave2_wave3	1	hibpe+hearte+arthre	3	2.976	0.744	0.256	215	6	10001001
ELSA	wave2_wave3	2	hibpe+diabe+arthre	3	2.901	0.725	0.275	137	7	11000001
ELSA	wave2_wave3	3	hibpe+arthre	2	2.267	0.756	0.244	641	13	10000001
ELSA	wave2_wave3	4	hibpe+hearte	2	2.005	0.668	0.332	229	10	10001000
ELSA	wave2_wave3	5	psyche+arthre	2	1.974	0.658	0.342	107	7	00000011
ELSA	wave2_wave3	6	hearte+arthre	2	1.948	0.649	0.351	179	8	00001001
ELSA	wave2_wave3	7	hibpe+psyche	2	1.891	0.63	0.37	104	5	10000010
ELSA	wave2_wave3	8	hibpe+diabe	2	1.851	0.617	0.383	166	6	11000000
ELSA	wave2_wave3	9	hearte	1	1.527	0.763	0.237	271	11	00001000
ELSA	wave2_wave3	10	hibpe	1	1.497	0.748	0.252	1,017	16	10000000
ELSA	wave3_wave4	1	hibpe+diabe+arthre	3	2.769	0.692	0.308	124	7	11000001
ELSA	wave3_wave4	2	hibpe+hearte+arthre	3	2.757	0.689	0.311	223	7	10001001
ELSA	wave3_wave4	3	hibpe+arthre	2	2.239	0.746	0.254	567	12	10000001
ELSA	wave3_wave4	4	hearte+arthre	2	2.234	0.745	0.255	158	7	00001001
ELSA	wave3_wave4	5	hibpe+hearte	2	2.112	0.704	0.296	218	9	10001000
ELSA	wave3_wave4	6	hibpe+diabe	2	2.027	0.676	0.324	157	7	11000000
ELSA	wave3_wave4	7	psyche+arthre	2	1.844	0.615	0.385	112	6	00000011
ELSA	wave3_wave4	8	hibpe+psyche	2	1.682	0.561	0.439	101	7	10000010
ELSA	wave3_wave4	9	arthre	1	1.549	0.774	0.226	782	16	00000001
ELSA	wave3_wave4	10	hibpe	1	1.458	0.729	0.271	878	21	10000000
ELSA	wave4_wave5	1	hibpe+hearte+arthre	3	2.915	0.729	0.271	215	8	10001001
ELSA	wave4_wave5	2	hibpe+diabe+arthre	3	2.697	0.674	0.326	113	5	11000001
ELSA	wave4_wave5	3	hibpe+hearte	2	2.136	0.712	0.288	211	11	10001000
ELSA	wave4_wave5	4	hibpe+arthre	2	2.081	0.694	0.306	517	10	10000001
ELSA	wave4_wave5	5	hearte+arthre	2	2.004	0.668	0.332	161	8	00001001
ELSA	wave4_wave5	6	hibpe+diabe	2	1.972	0.657	0.343	142	6	11000000
ELSA	wave4_wave5	7	arthre	1	1.532	0.766	0.234	706	16	00000001
ELSA	wave4_wave5	8	hibpe	1	1.42	0.71	0.29	725	19	10000000
ELSA	wave4_wave5	9	cancre	1	1.418	0.709	0.291	175	8	00100000
ELSA	wave4_wave5	10	psyche	1	1.273	0.636	0.364	159	11	00000010
HRS	wave1_wave2	1	hibpe+hearte+stroke+arthre	4	4.126	0.825	0.175	102	4	10001101
HRS	wave1_wave2	2	hibpe+diabe+hearte+psyche+arthre	5	3.883	0.647	0.353	112	5	11001011
HRS	wave1_wave2	3	hibpe+diabe+cancre+arthre	4	3.714	0.743	0.257	142	6	11100001
HRS	wave1_wave2	4	hibpe+cancre+hearte+arthre	4	3.695	0.739	0.261	120	6	10101001
HRS	wave1_wave2	5	hibpe+diabe+psyche+arthre	4	3.503	0.701	0.299	182	6	11000011
HRS	wave1_wave2	6	hibpe+diabe+hearte+arthre	4	3.365	0.673	0.327	321	7	11001001
HRS	wave1_wave2	7	hibpe+hearte+psyche+arthre	4	3.318	0.664	0.336	152	5	10001011
HRS	wave1_wave2	8	hibpe+cancre+arthre	3	3.094	0.773	0.227	333	11	10100001
HRS	wave1_wave2	9	hibpe+hearte+arthre	3	2.861	0.715	0.285	589	13	10001001
HRS	wave1_wave2	10	hibpe+diabe+arthre	3	2.859	0.715	0.285	680	14	11000001
HRS	wave2_wave3	1	hibpe+diabe+hearte+arthre	4	4.052	0.81	0.19	322	8	11001001
HRS	wave2_wave3	2	hibpe+diabe+cancre+arthre	4	3.923	0.785	0.215	134	5	11100001
HRS	wave2_wave3	3	hibpe+diabe+hearte+psyche+arthre	5	3.771	0.629	0.371	118	4	11001011
HRS	wave2_wave3	4	hibpe+cancre+hearte+arthre	4	3.4	0.68	0.32	126	6	10101001
HRS	wave2_wave3	5	hibpe+hearte+stroke+arthre	4	3.274	0.655	0.345	110	7	10001101
HRS	wave2_wave3	6	hibpe+diabe+psyche+arthre	4	3.22	0.644	0.356	205	6	11000011
HRS	wave2_wave3	7	hibpe+hearte+arthre	3	2.923	0.731	0.269	578	10	10001001
HRS	wave2_wave3	8	hibpe+diabe+arthre	3	2.835	0.709	0.291	684	13	11000001
HRS	wave2_wave3	9	hibpe+hearte+psyche+arthre	4	2.829	0.566	0.434	163	7	10001011
HRS	wave2_wave3	10	hibpe+psyche+arthre	3	2.822	0.705	0.295	392	11	10000011
HRS	wave3_wave4	1	hibpe+diabe+hearte+psyche+arthre	5	4.167	0.694	0.306	129	6	11001011
HRS	wave3_wave4	2	hibpe+diabe+psyche+arthre	4	3.631	0.726	0.274	201	7	11000011
HRS	wave3_wave4	3	hibpe+cancre+hearte+arthre	4	3.507	0.701	0.299	116	5	10101001
HRS	wave3_wave4	4	hibpe+diabe+hearte+arthre	4	3.33	0.666	0.334	300	8	11001001
HRS	wave3_wave4	5	hibpe+hearte+psyche+arthre	4	2.949	0.59	0.41	149	6	10001011
HRS	wave3_wave4	6	hibpe+diabe+arthre	3	2.88	0.72	0.28	651	12	11000001
HRS	wave3_wave4	7	hibpe+diabe+cancre+arthre	4	2.878	0.576	0.424	132	7	11100001
HRS	wave3_wave4	8	hibpe+psyche+arthre	3	2.795	0.699	0.301	372	11	10000011
HRS	wave3_wave4	9	hibpe+hearte+arthre	3	2.784	0.696	0.304	552	10	10001001
HRS	wave3_wave4	10	hibpe+cancre+arthre	3	2.649	0.662	0.338	306	11	10100001
HRS	wave4_wave5	1	hibpe+diabe+hearte+psyche+arthre	5	4.77	0.795	0.205	135	3	11001011
HRS	wave4_wave5	2	hibpe+cancre+hearte+arthre	4	3.761	0.752	0.248	107	5	10101001
HRS	wave4_wave5	3	hibpe+diabe+hearte+arthre	4	3.726	0.745	0.255	311	8	11001001
HRS	wave4_wave5	4	hibpe+hearte+psyche+arthre	4	3.531	0.706	0.294	131	6	10001011
HRS	wave4_wave5	5	hibpe+diabe+cancre+arthre	4	3.451	0.69	0.31	115	6	11100001
HRS	wave4_wave5	6	hibpe+diabe+psyche+arthre	4	3.45	0.69	0.31	254	7	11000011
HRS	wave4_wave5	7	hibpe+diabe+arthre	3	3.033	0.758	0.242	702	12	11000001
HRS	wave4_wave5	8	hibpe+hearte+arthre	3	2.95	0.738	0.262	509	10	10001001
HRS	wave4_wave5	9	hibpe+psyche+arthre	3	2.924	0.731	0.269	387	11	10000011

cohort	window	rank	state label	disease count	LockInScore	LockInCore	EntropyNorm	from n	out degree	state id
HRS	wave4_wave5	10	hibpe+cancre+arthre	3	2.725	0.681	0.319	270	10	10100001
HRS	wave5_wave6	1	hibpe+cancre+hearte+arthre	4	3.991	0.798	0.202	113	6	10101001
HRS	wave5_wave6	2	hibpe+diabe+hearte+psyche+arthre	5	3.885	0.647	0.353	124	4	11001011
HRS	wave5_wave6	3	hibpe+diabe+hearte+arthre	4	3.802	0.76	0.24	278	7	11001001
HRS	wave5_wave6	4	hibpe+diabe+psyche+arthre	4	3.385	0.677	0.323	227	7	11000011
HRS	wave5_wave6	5	hibpe+hearte+psyche+arthre	4	3.369	0.674	0.326	129	6	10001011
HRS	wave5_wave6	6	hibpe+diabe+cancre+arthre	4	3.19	0.638	0.362	119	6	11100001
HRS	wave5_wave6	7	hibpe+diabe+arthre	3	3.104	0.776	0.224	674	12	11000001
HRS	wave5_wave6	8	hibpe+psyche+arthre	3	2.788	0.697	0.303	359	10	10000011
HRS	wave5_wave6	9	hibpe+hearte+arthre	3	2.664	0.666	0.334	460	10	10001001
HRS	wave5_wave6	10	hibpe+cancre+arthre	3	2.636	0.659	0.341	237	8	10100001

Table S2C. Cohort- and window-level summaries of branching and lock-in intensity among top-ranked states.

cohort	window	mean branch top	n branch top	mean lockin top	n lockin top
CHARLS	wave1_wave2	0.203	10	2.158	10
CHARLS	wave2_wave3	0.642	10	1.834	10
CHARLS	wave3_wave4	1.206	10	1.713	10
CHARLS	wave4_wave5	0.705	10	2.608	10
ELSA	wave1_wave2	0.327	10	2.164	10
ELSA	wave2_wave3	0.308	10	2.084	10
ELSA	wave3_wave4	0.321	10	2.067	10
ELSA	wave4_wave5	0.317	10	1.945	10
HRS	wave1_wave2	0.508	10	3.442	10
HRS	wave2_wave3	0.493	10	3.305	10
HRS	wave3_wave4	0.551	10	3.157	10
HRS	wave4_wave5	0.487	10	3.432	10
HRS	wave5_wave6	0.45	10	3.281	10

Table S3. Recurrent high-impact multimorbidity states across cohorts and dynamic signatures.

state id	state label	disease count	signature	cohorts present	windows present	cohorts list	max score	median score
10001001	hibpe+hearte+arthre	3	LockIn	3	13	CHARLS, ELSA, HRS	3.44	2.861
00001000	hearte	1	Branching	3	12	CHARLS, ELSA, HRS	1.366	0.486
11000001	hibpe+diabe+arthre	3	LockIn	3	11	CHARLS, ELSA, HRS	3.104	2.835
10000000	hibpe	1	Branching	3	11	CHARLS, ELSA, HRS	1.187	0.429
00000000	Healthy	0	Branching	3	11	CHARLS, ELSA, HRS	1.067	0.372
01000000	diabe	1	Branching	3	10	CHARLS, ELSA, HRS	1.284	0.558
10001000	hibpe+hearte	2	Branching	3	10	CHARLS, ELSA, HRS	1.182	0.514
11000000	hibpe+diabe	2	Branching	3	8	CHARLS, ELSA, HRS	1.079	0.351
00000001	arthre	1	Branching	3	6	CHARLS, ELSA, HRS	0.43	0.255
00000010	psyche	1	Branching	2	9	ELSA, HRS	0.714	0.442
10000001	hibpe+arthre	2	LockIn	2	8	CHARLS, ELSA	2.43	2.17
10001000	hibpe+hearte	2	LockIn	2	8	CHARLS, ELSA	2.225	2.061
11000000	hibpe+diabe	2	LockIn	2	7	CHARLS, ELSA	2.379	1.879
00001001	hearte+arthre	2	LockIn	2	7	CHARLS, ELSA	2.245	2.004
00001001	hearte+arthre	2	Branching	2	7	CHARLS, ELSA	1.223	0.297
11001001	hibpe+diabe+hearte+arthre	4	LockIn	2	6	CHARLS, HRS	4.052	3.622
01000001	diabe+arthre	2	Branching	2	6	CHARLS, HRS	1.372	0.473
10000010	hibpe+psyche	2	Branching	2	6	ELSA, HRS	0.568	0.482
00000001	arthre	1	LockIn	2	5	CHARLS, ELSA	1.662	1.549
10000000	hibpe	1	LockIn	2	5	CHARLS, ELSA	1.616	1.489
00010001	lunge+arthre	2	Branching	2	5	CHARLS, HRS	1.114	0.579
10010001	hibpe+lunge+arthre	3	Branching	2	5	CHARLS, HRS	0.56	0.489
00100000	cancre	1	Branching	2	5	ELSA, HRS	0.49	0.444
00000011	psyche+arthre	2	Branching	2	3	ELSA, HRS	0.381	0.295

Table S4. Sensitivity analysis of transition-network perturbations under stabilization of key dynamic states across cohorts and survey windows.

cohort	window	scenario	alpha	L1 distance
CHARLS	wave1_wave2	Stabilize_branching_top5	0.3	0.042
CHARLS	wave1_wave2	Stabilize_acceleration_top5	0.3	0.042
CHARLS	wave2_wave3	Stabilize_branching_top5	0.3	0.038
CHARLS	wave2_wave3	Stabilize_acceleration_top5	0.3	0.038
CHARLS	wave3_wave4	Stabilize_branching_top5	0.3	0.036
CHARLS	wave3_wave4	Stabilize_acceleration_top5	0.3	0.036
CHARLS	wave4_wave5	Stabilize_branching_top5	0.3	0.029
CHARLS	wave4_wave5	Stabilize_acceleration_top5	0.3	0.043
ELSA	wave1_wave2	Stabilize_branching_top5	0.3	0.036
ELSA	wave1_wave2	Stabilize_acceleration_top5	0.3	0.039
ELSA	wave2_wave3	Stabilize_branching_top5	0.3	0.045

cohort	window	scenario	alpha	L1 distance
ELSA	wave2_wave3	Stabilize_acceleration_top5	0.3	0.037
ELSA	wave3_wave4	Stabilize_branching_top5	0.3	0.039
ELSA	wave3_wave4	Stabilize_acceleration_top5	0.3	0.035
ELSA	wave4_wave5	Stabilize_branching_top5	0.3	0.044
ELSA	wave4_wave5	Stabilize_acceleration_top5	0.3	0.044
HRS	wave1_wave2	Stabilize_branching_top5	0.3	0.032
HRS	wave1_wave2	Stabilize_acceleration_top5	0.3	0.028
HRS	wave2_wave3	Stabilize_branching_top5	0.3	0.028
HRS	wave2_wave3	Stabilize_acceleration_top5	0.3	0.023
HRS	wave3_wave4	Stabilize_branching_top5	0.3	0.009
HRS	wave3_wave4	Stabilize_acceleration_top5	0.3	0.01
HRS	wave4_wave5	Stabilize_branching_top5	0.3	0.022
HRS	wave4_wave5	Stabilize_acceleration_top5	0.3	0.021
HRS	wave5_wave6	Stabilize_branching_top5	0.3	0.019
HRS	wave5_wave6	Stabilize_acceleration_top5	0.3	0.014

Table S5. Newly reported conditions and apparent losses repaired by the carry-forward rule by cohort and transition window.

cohort	window	N pair	participants with any newly reported condition	participants with any apparent loss carried forward	proportion with any newly reported condition	proportion with any apparent loss carried forward	total new condition reports	total apparent loss reports repaired
CHARLS	wave1_wave2	14364	1491	0	0.1038	0	1680	0
CHARLS	wave2_wave3	14361	3650	0	0.2542	0	4297	0
CHARLS	wave3_wave4	13969	4967	2284	0.3556	0.1635	6395	2586
CHARLS	wave4_wave5	17616	3896	2824	0.2212	0.1603	4719	3290
ELSA	wave1_wave2	8999	1216	0	0.1351	0	1346	0
ELSA	wave2_wave3	8865	1278	0	0.1442	0	1432	0
ELSA	wave3_wave4	8088	1229	0	0.152	0	1360	0
ELSA	wave4_wave5	7146	1152	0	0.1612	0	1290	0
HRS	wave1_wave2	19783	3469	0	0.1754	0	4032	0
HRS	wave2_wave3	18075	3095	0	0.1712	0	3530	0
HRS	wave3_wave4	15908	3120	0	0.1961	0	3619	0
HRS	wave4_wave5	16604	2895	0	0.1744	0	3310	0
HRS	wave5_wave6	14257	2402	0	0.1685	0	2691	0

Table S6. Sensitivity analysis without the irreversibility rule.

cohort	window	metric	primary top10	sensitivity top10	top10 jaccard
CHARLS	wave1_wave2	AccumSpeed	00011000; 00001000; 00000100; 10000100; 01000000; 00000010; 00001001; 00010001; 00011001; 00000000	00000000; 00000001; 10000000; 00010000; 00001000; 01000000; 00000010; 00000100; 10000001; 10001000	0.3333
CHARLS	wave1_wave2	BranchScore	00011000; 00001000; 00000100; 01000000; 10000100; 00000000; 00001001; 00000010; 10000000; 00010000	00000000; 00000001; 10000000; 10000001; 00010000; 00001000; 10001000; 00010001; 00001001; 10001001	0.3333
CHARLS	wave1_wave2	LockInScore	11001001; 10011001; 10010001; 10001001; 11000001; 11001000; 00011001; 10000001; 11000000; 00001001	10011001; 11001001; 10001001; 10010001; 00011001; 11000001; 11001000; 10000001; 10001000; 00010001	0.6667
CHARLS	wave2_wave3	AccumSpeed	00010000; 00000010; 01000000; 00001000; 10000000; 11001000; 10000100; 01000001; 10001000; 00000000	00000000; 00000001; 10000000; 00010000; 00001000; 01000000; 00000010; 10000001; 10001000; 00010001	0.5385
CHARLS	wave2_wave3	BranchScore	00010000; 00000010; 01000000; 00001000; 10000000; 00000000; 11000000; 11001000; 10001000; 01000001	00000000; 00000001; 10000000; 10000001; 00010000; 00001000; 10001000; 00010001; 00001001; 10001001	0.3333
CHARLS	wave2_wave3	LockInScore	10011001; 11001001; 00011001; 11000001; 10001001; 10010001; 11001000; 10000001; 00010001; 11000000	10011001; 11001001; 10001001; 10010001; 00011001; 11000001; 11001000; 10011000; 10000001; 10001000	0.6667
CHARLS	wave3_wave4	AccumSpeed	10011000; 01000000; 01000001; 00001000; 00000101; 10010000; 00001001; 10001000; 11000000; 00010001	00000000; 00000001; 10000000; 00010000; 00001000; 01000000; 00000010; 00100000; 10000001; 00010001	0.1765
CHARLS	wave3_wave4	BranchScore	01000001; 00001000; 01000000; 10011000; 00000101; 00001001; 10000000; 10010000; 10001000; 00010001	00000000; 00000001; 10000000; 10000001; 00010001; 00010000; 10001001; 00001001; 10001000; 00001000	0.3333
CHARLS	wave3_wave4	LockInScore	10011001; 11001001; 11001000; 10001001; 10010001; 00011001; 10000001; 10000101; 10011000; 00010001	10011001; 11001001; 10001001; 10010001; 11000001; 00011001; 11001000; 10011000; 10000101; 10000001	0.8182
CHARLS	wave4_wave5	AccumSpeed	00001000; 10001100; 10000100; 10011000; 00100001; 01001000; 10010000; 00010000; 00000101; 01000000	00000000; 00000001; 10000000; 00010000; 00001000; 01000000; 00000100; 00100000; 00000010; 10000001	0.1765
CHARLS	wave4_wave5	BranchScore	00001000; 10000100; 10011000; 00010000; 10010000; 00100001; 01000000; 10001100; 00000101; 00000010	00000000; 00000001; 10000000; 10000001; 00010000; 10001001; 10001000; 00010001; 00001000; 11000000	0.1111
CHARLS	wave4_wave5	LockInScore	11011001; 11001001; 10011001; 11010001; 11001000; 00011001; 10001001; 11000001; 10001101; 10010001	11011001; 10011001; 11001001; 10001101; 11010001; 10001001; 10010001; 11000001; 11001000; 00011001	1
ELSA	wave1_wave2	AccumSpeed	01000000; 10000010; 00001000; 10100001; 00010000; 11000000; 01000001; 11000001; 00000010; 00100001	00000000; 10000000; 00000001; 00001000; 00000010; 00100000; 01000000; 00010000; 10000001; 10001000	0.25
ELSA	wave1_wave2	BranchScore	01000000; 10000010; 00001000; 00000010; 00100001; 00010000; 11000001; 00000000; 11000000; 10100001	00000000; 10000000; 00000001; 10000001; 00001000; 10001000; 10001001; 00000010; 00100000; 00001001	0.1765
ELSA	wave1_wave2	LockInScore	11001001; 10001001; 10010001; 11000001; 10000011; 10100001; 10000001; 10001000; 00001001; 00000011	11001001; 10001001; 11000001; 10000011; 10100001; 10010001; 10000001; 10001000; 00001001; 11000000	0.8182
ELSA	wave2_wave3	AccumSpeed	10100000; 00000010; 00010000; 00000001; 10000011; 01000000; 10001000; 11000000; 00001001; 00000011	00000000; 10000000; 00000001; 00001000; 00100000; 00000010; 01000000; 00010000; 10000001; 10001000	0.3333
ELSA	wave2_wave3	BranchScore	00000010; 00000001; 10100000; 00010000; 01000000; 10001000; 10000011; 00000000; 00001001; 10000000	00000000; 10000000; 00000001; 10000001; 00001000; 10001000; 10001001; 00100000; 00000010; 00001001	0.4286
ELSA	wave2_wave3	LockInScore	11001001; 10001001; 11000001; 10100001; 10000011; 10000001; 10000100; 00100001; 10001000; 00000011	11001001; 10001001; 11000001; 10000011; 10100001; 10000001; 10001000; 00001001; 11000000; 00000011	0.6667

cohort	window	metric	primary top10	sensitivity top10	top10 jaccard
ELSA	wave3_wave4	AccumSpeed	10000010; 00100000; 00010000; 10000000; 11000001; 00000011; 10100000; 00000000; 00001000; 00100001	00000000; 10000000; 00000001; 00001000; 00100000; 00000010; 01000000; 00010000; 10000001; 10001000	0.3333
ELSA	wave3_wave4	BranchScore	00100000; 10000000; 10000010; 00010000; 00000000; 11000001; 00000011; 00100001; 00001000; 00000010	00000000; 10000000; 00000001; 10000001; 00001000; 10001001; 10001000; 00100000; 00000010; 00001001	0.3333
ELSA	wave3_wave4	LockInScore	11001001; 10100001; 11000001; 10001001; 10000011; 11001000; 10000001; 00001001; 10001000; 00100001	11001001; 10001001; 11000001; 10000011; 10100001; 11001000; 10000001; 10001000; 00001001; 11000000	0.8182
ELSA	wave4_wave5	AccumSpeed	01000000; 10000011; 00000010; 00010000; 00100001; 00001000; 10000010; 10000000; 11001000; 00000000	00000000; 10000000; 00000001; 00001000; 00100000; 00000010; 01000000; 00010000; 10000001; 10001000	0.4286
ELSA	wave4_wave5	BranchScore	01000000; 00010000; 10000011; 00000010; 10000000; 00001000; 00100001; 10000010; 00000000; 10001000	00000000; 10000000; 00000001; 10000001; 00001000; 10001001; 10001000; 00100000; 00001001; 00000010	0.3333
ELSA	wave4_wave5	LockInScore	11001001; 10001001; 11000001; 10100001; 10000011; 00000011; 10001000; 10000001; 11001000; 00001001	11001001; 10001001; 11000001; 10000011; 10100001; 11001000; 10000001; 10001000; 00001001; 11000000	0.8182
HRS	wave1_wave2	AccumSpeed	00000101; 00000100; 00010001; 10010000; 11010001; 10000010; 11000010; 00000010; 10000100; 10010001	00000000; 10000000; 00000001; 01000000; 00000010; 00001000; 00100000; 00010000; 00000100; 10000001	0.1111
HRS	wave1_wave2	BranchScore	00000101; 00010001; 00000100; 11010001; 00000010; 10000010; 10000100; 10010000; 10001000; 10010001	00000000; 10000000; 10000001; 00000001; 11000001; 11000000; 10001001; 10000011; 00000011; 10100001	0
HRS	wave1_wave2	LockInScore	11011011; 10001101; 11101001; 11001011; 10011011; 10011001; 11100001; 10101001; 11001101; 11000011	11011011; 11001011; 11001101; 11101001; 10011011; 11001001; 11000011; 10001011; 11100001; 10101001	0.6667
HRS	wave2_wave3	AccumSpeed	01000000; 00010000; 10010000; 10000100; 00010011; 00001000; 00001011; 10010001; 10001000; 00000010	00000000; 10000000; 00000001; 01000000; 00000010; 00100000; 00001000; 00010000; 10000001; 11000000	0.25
HRS	wave2_wave3	BranchScore	00010000; 01000000; 00001000; 10000100; 10010000; 00010011; 00001011; 10001000; 00010001; 00000010	00000000; 10000001; 10000000; 00000001; 11000001; 11000000; 10001001; 10000011; 00000011; 10100001	0
HRS	wave2_wave3	LockInScore	11001111; 11001101; 11011001; 11011011; 10100011; 11001001; 11100001; 11101001; 11001011; 10011011	11011011; 11001111; 11001011; 11101001; 10011011; 11001101; 11011001; 11010011; 11001001; 11000011	0.6667
HRS	wave3_wave4	AccumSpeed	00010000; 00010001; 00000010; 10000100; 10011001; 10000101; 01001001; 01000000; 11100000; 10000010	00000000; 10000000; 00000001; 00000010; 01000000; 00100000; 00001000; 00010000; 10000001; 11000000	0.1765
HRS	wave3_wave4	BranchScore	00010000; 00010001; 00000010; 10000100; 10000101; 10011001; 01000000; 10001000; 00000000; 00001000	00000000; 10000001; 10000000; 00000001; 11000001; 10001001; 11000000; 10000011; 00000011; 10100001	0.0526
HRS	wave3_wave4	LockInScore	11001101; 11101001; 11001011; 11000011; 10101001; 11001001; 11010011; 10001101; 10010011; 11010001	11001011; 10011011; 11001101; 11101001; 11010011; 11001001; 11000011; 10001011; 11100001; 10101001	0.5385
HRS	wave4_wave5	AccumSpeed	00010000; 10000100; 10010000; 11100000; 00000010; 10001000; 10010001; 11000010; 10000010; 00100000	00000000; 10000000; 00000001; 00000010; 01000000; 00100000; 00001000; 00010000; 10000001; 11000000	0.1765
HRS	wave4_wave5	BranchScore	10000100; 00010000; 00000010; 10010000; 10001000; 11100000; 10000010; 00100000; 01000001; 10010001	00000000; 10000000; 10000001; 00000001; 11000001; 11000000; 10001001; 10000011; 00000011; 11001001	0
HRS	wave4_wave5	LockInScore	11001011; 11011011; 11101001; 11001101; 10011011; 10101001; 11001001; 11000101; 10001011; 11100001	11011011; 11001011; 11101001; 11001101; 10011011; 11010011; 11001001; 11000011; 10001011; 11100001	0.6667
HRS	wave5_wave6	AccumSpeed	00010001; 01000000; 10001000; 00001000; 00100000; 10000010; 01000001; 00010011; 10000000; 00000010	00000000; 10000000; 00000001; 01000000; 00000010; 00100000; 00001000; 10000001; 11000000; 00000011	0.3333
HRS	wave5_wave6	BranchScore	00010001; 01000000; 10001000; 00001000; 10000010; 00100000; 10000000; 00000010; 01000001; 00100001	00000000; 10000001; 10000000; 00000001; 11000001; 11000000; 10001001; 10000011; 00000011; 11001001	0.0526
HRS	wave5_wave6	LockInScore	11101001; 11011011; 11010011; 10001101; 10011011; 11001101; 10101001; 11001011; 11001001; 10100011	11011011; 11001011; 11101001; 10011011; 11010011; 11001101; 11011001; 11001001; 11000011; 10001011	0.5385

Table S7. Threshold sensitivity analysis for reproducible states using alternative support thresholds.

cohort	window	threshold from n	metric	eligible states	top-10 Jaccard vs >=50
CHARLS	wave1_wave2	30	AccumSpeed	29	0.8182
CHARLS	wave1_wave2	30	BranchScore	29	0.8182
CHARLS	wave1_wave2	30	LockInScore	29	0.8182
CHARLS	wave1_wave2	50	AccumSpeed	24	1
CHARLS	wave1_wave2	50	BranchScore	24	1
CHARLS	wave1_wave2	50	LockInScore	24	1
CHARLS	wave1_wave2	100	AccumSpeed	13	0.3333
CHARLS	wave1_wave2	100	BranchScore	13	0.4286
CHARLS	wave1_wave2	100	LockInScore	13	0.25
CHARLS	wave2_wave3	30	AccumSpeed	30	0.5385
CHARLS	wave2_wave3	30	BranchScore	30	0.6667
CHARLS	wave2_wave3	30	LockInScore	30	0.8182
CHARLS	wave2_wave3	50	AccumSpeed	24	1
CHARLS	wave2_wave3	50	BranchScore	24	1
CHARLS	wave2_wave3	50	LockInScore	24	1
CHARLS	wave2_wave3	100	AccumSpeed	15	0.5385
CHARLS	wave2_wave3	100	BranchScore	15	0.6667
CHARLS	wave2_wave3	100	LockInScore	15	0.3333
CHARLS	wave3_wave4	30	AccumSpeed	34	0.8182
CHARLS	wave3_wave4	30	BranchScore	34	0.8182
CHARLS	wave3_wave4	30	LockInScore	34	0.5385
CHARLS	wave3_wave4	50	AccumSpeed	27	1
CHARLS	wave3_wave4	50	BranchScore	27	1
CHARLS	wave3_wave4	50	LockInScore	27	1
CHARLS	wave3_wave4	100	AccumSpeed	18	0.6667
CHARLS	wave3_wave4	100	BranchScore	18	0.6667
CHARLS	wave3_wave4	100	LockInScore	18	0.4286
CHARLS	wave4_wave5	30	AccumSpeed	47	0.6667
CHARLS	wave4_wave5	30	BranchScore	47	0.8182

cohort	window	threshold from n	metric	eligible states	top-10 Jaccard vs >=50
CHARLS	wave4_wave5	30	LockInScore	47	0.4286
CHARLS	wave4_wave5	50	AccumSpeed	37	1
CHARLS	wave4_wave5	50	BranchScore	37	1
CHARLS	wave4_wave5	50	LockInScore	37	1
CHARLS	wave4_wave5	100	AccumSpeed	23	0.4286
CHARLS	wave4_wave5	100	BranchScore	23	0.4286
CHARLS	wave4_wave5	100	LockInScore	23	0.5385
ELSA	wave1_wave2	30	AccumSpeed	30	0.5385
ELSA	wave1_wave2	30	BranchScore	30	0.6667
ELSA	wave1_wave2	30	LockInScore	30	0.5385
ELSA	wave1_wave2	50	AccumSpeed	24	1
ELSA	wave1_wave2	50	BranchScore	24	1
ELSA	wave1_wave2	50	LockInScore	24	1
ELSA	wave1_wave2	100	AccumSpeed	15	0.4286
ELSA	wave1_wave2	100	BranchScore	15	0.5385
ELSA	wave1_wave2	100	LockInScore	15	0.3333
ELSA	wave2_wave3	30	AccumSpeed	33	0.3333
ELSA	wave2_wave3	30	BranchScore	33	0.8182
ELSA	wave2_wave3	30	LockInScore	33	0.3333
ELSA	wave2_wave3	50	AccumSpeed	22	1
ELSA	wave2_wave3	50	BranchScore	22	1
ELSA	wave2_wave3	50	LockInScore	22	1
ELSA	wave2_wave3	100	AccumSpeed	15	0.5385
ELSA	wave2_wave3	100	BranchScore	15	0.5385
ELSA	wave2_wave3	100	LockInScore	15	0.3333
ELSA	wave3_wave4	30	AccumSpeed	33	0.4286
ELSA	wave3_wave4	30	BranchScore	33	0.4286
ELSA	wave3_wave4	30	LockInScore	33	0.4286
ELSA	wave3_wave4	50	AccumSpeed	24	1
ELSA	wave3_wave4	50	BranchScore	24	1
ELSA	wave3_wave4	50	LockInScore	24	1
ELSA	wave3_wave4	100	AccumSpeed	15	0.5385
ELSA	wave3_wave4	100	BranchScore	15	0.6667
ELSA	wave3_wave4	100	LockInScore	15	0.3333
ELSA	wave4_wave5	30	AccumSpeed	33	0.5385
ELSA	wave4_wave5	30	BranchScore	33	0.6667
ELSA	wave4_wave5	30	LockInScore	33	0.3333
ELSA	wave4_wave5	50	AccumSpeed	22	1
ELSA	wave4_wave5	50	BranchScore	22	1
ELSA	wave4_wave5	50	LockInScore	22	1
ELSA	wave4_wave5	100	AccumSpeed	12	0.25
ELSA	wave4_wave5	100	BranchScore	12	0.3333
ELSA	wave4_wave5	100	LockInScore	12	0.3333
HRS	wave1_wave2	30	AccumSpeed	71	0.4286
HRS	wave1_wave2	30	BranchScore	71	0.6667
HRS	wave1_wave2	30	LockInScore	71	0.25
HRS	wave1_wave2	50	AccumSpeed	54	1
HRS	wave1_wave2	50	BranchScore	54	1
HRS	wave1_wave2	50	LockInScore	54	1
HRS	wave1_wave2	100	AccumSpeed	31	0.25
HRS	wave1_wave2	100	BranchScore	31	0.3333
HRS	wave1_wave2	100	LockInScore	31	0.3333
HRS	wave2_wave3	30	AccumSpeed	71	0.3333
HRS	wave2_wave3	30	BranchScore	71	0.5385
HRS	wave2_wave3	30	LockInScore	71	0.8182
HRS	wave2_wave3	50	AccumSpeed	56	1
HRS	wave2_wave3	50	BranchScore	56	1
HRS	wave2_wave3	50	LockInScore	56	1
HRS	wave2_wave3	100	AccumSpeed	31	0.3333
HRS	wave2_wave3	100	BranchScore	31	0.3333
HRS	wave2_wave3	100	LockInScore	31	0.1765
HRS	wave3_wave4	30	AccumSpeed	67	0.3333
HRS	wave3_wave4	30	BranchScore	67	0.4286
HRS	wave3_wave4	30	LockInScore	67	0.3333
HRS	wave3_wave4	50	AccumSpeed	50	1
HRS	wave3_wave4	50	BranchScore	50	1
HRS	wave3_wave4	50	LockInScore	50	1
HRS	wave3_wave4	100	AccumSpeed	29	0.25
HRS	wave3_wave4	100	BranchScore	29	0.4286

cohort	window	threshold from n	metric	eligible states	top-10 Jaccard vs >=50
HRS	wave3_wave4	100	LockInScore	29	0.25
HRS	wave4_wave5	30	AccumSpeed	68	0.4286
HRS	wave4_wave5	30	BranchScore	68	0.5385
HRS	wave4_wave5	30	LockInScore	68	0.5385
HRS	wave4_wave5	50	AccumSpeed	49	1
HRS	wave4_wave5	50	BranchScore	49	1
HRS	wave4_wave5	50	LockInScore	49	1
HRS	wave4_wave5	100	AccumSpeed	29	0.3333
HRS	wave4_wave5	100	BranchScore	29	0.4286
HRS	wave4_wave5	100	LockInScore	29	0.3333
HRS	wave5_wave6	30	AccumSpeed	67	0.3333
HRS	wave5_wave6	30	BranchScore	67	0.3333
HRS	wave5_wave6	30	LockInScore	67	0.5385
HRS	wave5_wave6	50	AccumSpeed	48	1
HRS	wave5_wave6	50	BranchScore	48	1
HRS	wave5_wave6	50	LockInScore	48	1
HRS	wave5_wave6	100	AccumSpeed	29	0.6667
HRS	wave5_wave6	100	BranchScore	29	0.8182
HRS	wave5_wave6	100	LockInScore	29	0.1765

Table S8A. Disease-count-stratified dynamic measures in the primary irreversible analysis.

cohort	window	metric	disease count	n states	from n sum	score mean	score weighted mean	score median
CHARLS	wave1_wave2	AccumSpeed	0	1	6037	0.1249	0.1249	0.1249
CHARLS	wave1_wave2	AccumSpeed	1	7	4884	0.1351	0.1155	0.1333
CHARLS	wave1_wave2	AccumSpeed	2	9	2134	0.1257	0.1106	0.1122
CHARLS	wave1_wave2	AccumSpeed	3	5	539	0.0864	0.0816	0.0833
CHARLS	wave1_wave2	AccumSpeed	4	2	114	0.0702	0.0702	0.0702
CHARLS	wave1_wave2	BranchScore	0	1	6037	0.2214	0.2214	0.2214
CHARLS	wave1_wave2	BranchScore	1	7	4884	0.2268	0.1896	0.2143
CHARLS	wave1_wave2	BranchScore	2	9	2134	0.1864	0.163	0.1575
CHARLS	wave1_wave2	BranchScore	3	5	539	0.1091	0.1025	0.115
CHARLS	wave1_wave2	BranchScore	4	2	114	0.0836	0.0836	0.0836
CHARLS	wave1_wave2	LockInScore	0	1	6037	0.8382	0.8382	0.8382
CHARLS	wave1_wave2	LockInScore	1	7	4884	1.5063	1.6089	1.4772
CHARLS	wave1_wave2	LockInScore	2	9	2134	2.1594	2.2687	2.1803
CHARLS	wave1_wave2	LockInScore	3	5	539	2.957	3.033	3.0435
CHARLS	wave1_wave2	LockInScore	4	2	114	3.9353	3.9335	3.9353
CHARLS	wave2_wave3	AccumSpeed	0	1	5739	0.3112	0.3112	0.3112
CHARLS	wave2_wave3	AccumSpeed	1	6	4826	0.363	0.3058	0.3706
CHARLS	wave2_wave3	AccumSpeed	2	9	2233	0.2855	0.2727	0.3053
CHARLS	wave2_wave3	AccumSpeed	3	6	681	0.2717	0.2658	0.2828
CHARLS	wave2_wave3	AccumSpeed	4	2	128	0.1355	0.1328	0.1355
CHARLS	wave2_wave3	BranchScore	0	1	5739	0.6494	0.6494	0.6494
CHARLS	wave2_wave3	BranchScore	1	6	4826	0.7035	0.6154	0.7128
CHARLS	wave2_wave3	BranchScore	2	9	2233	0.5059	0.4925	0.513
CHARLS	wave2_wave3	BranchScore	3	6	681	0.4524	0.4487	0.4692
CHARLS	wave2_wave3	BranchScore	4	2	128	0.1757	0.1716	0.1757
CHARLS	wave2_wave3	LockInScore	0	1	5739	0.7085	0.7085	0.7085
CHARLS	wave2_wave3	LockInScore	1	6	4826	1.1365	1.3162	1.0934
CHARLS	wave2_wave3	LockInScore	2	9	2233	1.7119	1.8352	1.6959
CHARLS	wave2_wave3	LockInScore	3	6	681	2.1518	2.1947	2.1962
CHARLS	wave2_wave3	LockInScore	4	2	128	3.1672	3.1951	3.1672
CHARLS	wave3_wave4	AccumSpeed	0	1	4337	0.436	0.436	0.436
CHARLS	wave3_wave4	AccumSpeed	1	7	4806	0.482	0.4638	0.4581
CHARLS	wave3_wave4	AccumSpeed	2	10	2677	0.5124	0.4897	0.5252
CHARLS	wave3_wave4	AccumSpeed	3	7	1033	0.4423	0.4288	0.4171
CHARLS	wave3_wave4	AccumSpeed	4	2	220	0.41	0.4	0.41
CHARLS	wave3_wave4	BranchScore	0	1	4337	1.0668	1.0668	1.0668
CHARLS	wave3_wave4	BranchScore	1	7	4806	1.0702	1.0872	1.0125
CHARLS	wave3_wave4	BranchScore	2	10	2677	1.0968	1.0883	1.1484
CHARLS	wave3_wave4	BranchScore	3	7	1033	0.8503	0.8198	0.8088
CHARLS	wave3_wave4	BranchScore	4	2	220	0.7556	0.7401	0.7556
CHARLS	wave3_wave4	LockInScore	0	1	4337	0.6378	0.6378	0.6378
CHARLS	wave3_wave4	LockInScore	1	7	4806	0.9964	1.1544	0.968
CHARLS	wave3_wave4	LockInScore	2	10	2677	1.3294	1.4959	1.2653
CHARLS	wave3_wave4	LockInScore	3	7	1033	1.7722	1.8334	1.8822
CHARLS	wave3_wave4	LockInScore	4	2	220	2.2643	2.3002	2.2643
CHARLS	wave4_wave5	AccumSpeed	0	1	5228	0.251	0.251	0.251
CHARLS	wave4_wave5	AccumSpeed	1	8	5570	0.2888	0.2794	0.2718
CHARLS	wave4_wave5	AccumSpeed	2	13	3467	0.3036	0.2766	0.3
CHARLS	wave4_wave5	AccumSpeed	3	10	1645	0.267	0.2571	0.2391
CHARLS	wave4_wave5	AccumSpeed	4	4	456	0.2351	0.2281	0.2326

cohort	window	metric	disease count	n states	from n sum	score mean	score weighted mean	score median
CHARLS	wave4_wave5	AccumSpeed	5	1	59	0.1017	0.1017	0.1017
CHARLS	wave4_wave5	BranchScore	0	1	5228	0.5368	0.5368	0.5368
CHARLS	wave4_wave5	BranchScore	1	8	5570	0.6125	0.5997	0.5973
CHARLS	wave4_wave5	BranchScore	2	13	3467	0.6039	0.554	0.6088
CHARLS	wave4_wave5	BranchScore	3	10	1645	0.4879	0.4742	0.4314
CHARLS	wave4_wave5	BranchScore	4	4	456	0.3907	0.3888	0.3843
CHARLS	wave4_wave5	BranchScore	5	1	59	0.1305	0.1305	0.1305
CHARLS	wave4_wave5	LockInScore	0	1	5228	0.7347	0.7347	0.7347
CHARLS	wave4_wave5	LockInScore	1	8	5570	1.269	1.3683	1.2111
CHARLS	wave4_wave5	LockInScore	2	13	3467	1.7582	1.902	1.7664
CHARLS	wave4_wave5	LockInScore	3	10	1645	2.3809	2.477	2.421
CHARLS	wave4_wave5	LockInScore	4	4	456	3.0592	3.1332	3.0957
CHARLS	wave4_wave5	LockInScore	5	1	59	4.1318	4.1318	4.1318
ELSA	wave1_wave2	AccumSpeed	0	1	2525	0.1568	0.1568	0.1568
ELSA	wave1_wave2	AccumSpeed	1	7	2921	0.1745	0.1565	0.1748
ELSA	wave1_wave2	AccumSpeed	2	10	1765	0.1515	0.1382	0.149
ELSA	wave1_wave2	AccumSpeed	3	5	586	0.1344	0.1195	0.1685
ELSA	wave1_wave2	AccumSpeed	4	1	50	0.1	0.1	0.1
ELSA	wave1_wave2	BranchScore	0	1	2525	0.2924	0.2924	0.2924
ELSA	wave1_wave2	BranchScore	1	7	2921	0.3173	0.2791	0.306
ELSA	wave1_wave2	BranchScore	2	10	1765	0.2437	0.2227	0.2348
ELSA	wave1_wave2	BranchScore	3	5	586	0.2046	0.1831	0.2628
ELSA	wave1_wave2	BranchScore	4	1	50	0.1292	0.1292	0.1292
ELSA	wave1_wave2	LockInScore	0	1	2525	0.7814	0.7814	0.7814
ELSA	wave1_wave2	LockInScore	1	7	2921	1.3908	1.4769	1.4171
ELSA	wave1_wave2	LockInScore	2	10	1765	2.0258	2.1375	2.0334
ELSA	wave1_wave2	LockInScore	3	5	586	2.8055	2.9368	2.6888
ELSA	wave1_wave2	LockInScore	4	1	50	3.447	3.447	3.447
ELSA	wave2_wave3	AccumSpeed	0	1	2489	0.1563	0.1563	0.1563
ELSA	wave2_wave3	AccumSpeed	1	7	2817	0.1681	0.1665	0.1795
ELSA	wave2_wave3	AccumSpeed	2	9	1672	0.1634	0.1615	0.1589
ELSA	wave2_wave3	AccumSpeed	3	4	513	0.1349	0.1287	0.1278
ELSA	wave2_wave3	AccumSpeed	4	1	51	0.0588	0.0588	0.0588
ELSA	wave2_wave3	BranchScore	0	1	2489	0.2994	0.2994	0.2994
ELSA	wave2_wave3	BranchScore	1	7	2817	0.3027	0.3075	0.3198
ELSA	wave2_wave3	BranchScore	2	9	1672	0.2615	0.2654	0.2598
ELSA	wave2_wave3	BranchScore	3	4	513	0.2076	0.1961	0.1891
ELSA	wave2_wave3	BranchScore	4	1	51	0.0592	0.0592	0.0592
ELSA	wave2_wave3	LockInScore	0	1	2489	0.796	0.796	0.796
ELSA	wave2_wave3	LockInScore	1	7	2817	1.4167	1.465	1.4633
ELSA	wave2_wave3	LockInScore	2	9	1672	1.9802	2.0635	1.9737
ELSA	wave2_wave3	LockInScore	3	4	513	2.838	2.8791	2.856
ELSA	wave2_wave3	LockInScore	4	1	51	4.1233	4.1233	4.1233
ELSA	wave3_wave4	AccumSpeed	0	1	2142	0.1746	0.1746	0.1746
ELSA	wave3_wave4	AccumSpeed	1	7	2486	0.1799	0.1758	0.166
ELSA	wave3_wave4	AccumSpeed	2	10	1599	0.1551	0.1526	0.1484
ELSA	wave3_wave4	AccumSpeed	3	5	570	0.1471	0.1474	0.1345
ELSA	wave3_wave4	AccumSpeed	4	1	53	0.0943	0.0943	0.0943
ELSA	wave3_wave4	BranchScore	0	1	2142	0.3393	0.3393	0.3393
ELSA	wave3_wave4	BranchScore	1	7	2486	0.3196	0.3219	0.2779
ELSA	wave3_wave4	BranchScore	2	10	1599	0.2449	0.2485	0.2502
ELSA	wave3_wave4	BranchScore	3	5	570	0.2214	0.2259	0.2084
ELSA	wave3_wave4	BranchScore	4	1	53	0.1214	0.1214	0.1214
ELSA	wave3_wave4	LockInScore	0	1	2142	0.7652	0.7652	0.7652
ELSA	wave3_wave4	LockInScore	1	7	2486	1.3664	1.4469	1.3854
ELSA	wave3_wave4	LockInScore	2	10	1599	2.0003	2.0883	2.0075
ELSA	wave3_wave4	LockInScore	3	5	570	2.699	2.7267	2.7573
ELSA	wave3_wave4	LockInScore	4	1	53	3.7519	3.7519	3.7519
ELSA	wave4_wave5	AccumSpeed	0	1	1664	0.1767	0.1767	0.1767
ELSA	wave4_wave5	AccumSpeed	1	7	2161	0.2127	0.1902	0.2208
ELSA	wave4_wave5	AccumSpeed	2	8	1387	0.1702	0.1702	0.173
ELSA	wave4_wave5	AccumSpeed	3	5	545	0.1699	0.1578	0.1416
ELSA	wave4_wave5	AccumSpeed	4	1	55	0.0727	0.0727	0.0727
ELSA	wave4_wave5	BranchScore	0	1	1664	0.335	0.335	0.335
ELSA	wave4_wave5	BranchScore	1	7	2161	0.4047	0.3574	0.4079
ELSA	wave4_wave5	BranchScore	2	8	1387	0.2779	0.2795	0.287
ELSA	wave4_wave5	BranchScore	3	5	545	0.2635	0.2475	0.2021
ELSA	wave4_wave5	BranchScore	4	1	55	0.0677	0.0677	0.0677
ELSA	wave4_wave5	LockInScore	0	1	1664	0.7661	0.7661	0.7661
ELSA	wave4_wave5	LockInScore	1	7	2161	1.3372	1.4123	1.3484
ELSA	wave4_wave5	LockInScore	2	8	1387	1.9775	2.0272	1.988
ELSA	wave4_wave5	LockInScore	3	5	545	2.517	2.645	2.6331
ELSA	wave4_wave5	LockInScore	4	1	55	3.1199	3.1199	3.1199
HRS	wave1_wave2	AccumSpeed	0	1	3544	0.2125	0.2125	0.2125
HRS	wave1_wave2	AccumSpeed	1	8	4907	0.2495	0.2256	0.2378
HRS	wave1_wave2	AccumSpeed	2	13	4626	0.244	0.2015	0.2421
HRS	wave1_wave2	AccumSpeed	3	16	2997	0.204	0.1882	0.1966

cohort	window	metric	disease count	n states	from n sum	score mean	score weighted mean	score median
HRS	wave1_wave2	AccumSpeed	4	11	1392	0.1587	0.1466	0.137
HRS	wave1_wave2	AccumSpeed	5	4	310	0.1502	0.1516	0.1463
HRS	wave1_wave2	AccumSpeed	6	1	53	0.0755	0.0755	0.0755
HRS	wave1_wave2	BranchScore	0	1	3544	0.4501	0.4501	0.4501
HRS	wave1_wave2	BranchScore	1	8	4907	0.4877	0.4393	0.4647
HRS	wave1_wave2	BranchScore	2	13	4626	0.4591	0.377	0.456
HRS	wave1_wave2	BranchScore	3	16	2997	0.3374	0.3266	0.3319
HRS	wave1_wave2	BranchScore	4	11	1392	0.2541	0.2295	0.201
HRS	wave1_wave2	BranchScore	5	4	310	0.2153	0.2181	0.2052
HRS	wave1_wave2	BranchScore	6	1	53	0.0819	0.0819	0.0819
HRS	wave1_wave2	LockInScore	0	1	3544	0.7725	0.7725	0.7725
HRS	wave1_wave2	LockInScore	1	8	4907	1.2933	1.4198	1.3065
HRS	wave1_wave2	LockInScore	2	13	4626	1.9055	2.1207	1.919
HRS	wave1_wave2	LockInScore	3	16	2997	2.4806	2.6981	2.4415
HRS	wave1_wave2	LockInScore	4	11	1392	3.3645	3.4298	3.3649
HRS	wave1_wave2	LockInScore	5	4	310	3.8045	3.8126	3.8678
HRS	wave1_wave2	LockInScore	6	1	53	5.0247	5.0247	5.0247
HRS	wave2_wave3	AccumSpeed	0	1	2819	0.1951	0.1951	0.1951
HRS	wave2_wave3	AccumSpeed	1	7	4190	0.2679	0.2248	0.2632
HRS	wave2_wave3	AccumSpeed	2	13	4337	0.2168	0.1914	0.1967
HRS	wave2_wave3	AccumSpeed	3	15	2890	0.1966	0.1799	0.1862
HRS	wave2_wave3	AccumSpeed	4	12	1484	0.157	0.1462	0.1657
HRS	wave2_wave3	AccumSpeed	5	6	428	0.1254	0.1262	0.1196
HRS	wave2_wave3	AccumSpeed	6	2	104	0.086	0.0865	0.086
HRS	wave2_wave3	BranchScore	0	1	2819	0.377	0.377	0.377
HRS	wave2_wave3	BranchScore	1	7	4190	0.545	0.4467	0.4954
HRS	wave2_wave3	BranchScore	2	13	4337	0.3923	0.3452	0.3638
HRS	wave2_wave3	BranchScore	3	15	2890	0.3264	0.3102	0.3335
HRS	wave2_wave3	BranchScore	4	12	1484	0.245	0.2249	0.2421
HRS	wave2_wave3	BranchScore	5	6	428	0.1715	0.173	0.1577
HRS	wave2_wave3	BranchScore	6	2	104	0.0991	0.0998	0.0991
HRS	wave2_wave3	LockInScore	0	1	2819	0.7553	0.7553	0.7553
HRS	wave2_wave3	LockInScore	1	7	4190	1.2929	1.4179	1.2617
HRS	wave2_wave3	LockInScore	2	13	4337	1.9296	2.0967	2.0191
HRS	wave2_wave3	LockInScore	3	15	2890	2.5051	2.7113	2.5029
HRS	wave2_wave3	LockInScore	4	12	1484	3.3456	3.4627	3.2471
HRS	wave2_wave3	LockInScore	5	6	428	3.9459	3.9247	3.8054
HRS	wave2_wave3	LockInScore	6	2	104	4.7929	4.7824	4.7929
HRS	wave3_wave4	AccumSpeed	0	1	2195	0.2547	0.2547	0.2547
HRS	wave3_wave4	AccumSpeed	1	7	3496	0.2938	0.2531	0.2722
HRS	wave3_wave4	AccumSpeed	2	11	3791	0.2563	0.2292	0.2457
HRS	wave3_wave4	AccumSpeed	3	15	2732	0.2136	0.1929	0.1993
HRS	wave3_wave4	AccumSpeed	4	11	1362	0.1998	0.188	0.1879
HRS	wave3_wave4	AccumSpeed	5	5	386	0.1693	0.1632	0.1395
HRS	wave3_wave4	BranchScore	0	1	2195	0.5578	0.5578	0.5578
HRS	wave3_wave4	BranchScore	1	7	3496	0.6005	0.5152	0.5495
HRS	wave3_wave4	BranchScore	2	11	3791	0.4828	0.4321	0.4384
HRS	wave3_wave4	BranchScore	3	15	2732	0.3523	0.3323	0.3229
HRS	wave3_wave4	BranchScore	4	11	1362	0.327	0.3116	0.3215
HRS	wave3_wave4	BranchScore	5	5	386	0.2558	0.247	0.2128
HRS	wave3_wave4	LockInScore	0	1	2195	0.7289	0.7289	0.7289
HRS	wave3_wave4	LockInScore	1	7	3496	1.2359	1.3652	1.2304
HRS	wave3_wave4	LockInScore	2	11	3791	1.8517	1.9955	1.9471
HRS	wave3_wave4	LockInScore	3	15	2732	2.408	2.6443	2.4347
HRS	wave3_wave4	LockInScore	4	11	1362	3.0233	3.1312	2.9938
HRS	wave3_wave4	LockInScore	5	5	386	3.7404	3.8212	4.1666
HRS	wave4_wave5	AccumSpeed	0	1	2569	0.2234	0.2234	0.2234
HRS	wave4_wave5	AccumSpeed	1	7	3661	0.2591	0.2262	0.2351
HRS	wave4_wave5	AccumSpeed	2	12	3797	0.2394	0.2023	0.2186
HRS	wave4_wave5	AccumSpeed	3	12	2627	0.216	0.1755	0.2249
HRS	wave4_wave5	AccumSpeed	4	11	1364	0.1394	0.1364	0.1391
HRS	wave4_wave5	AccumSpeed	5	5	416	0.1137	0.101	0.1127
HRS	wave4_wave5	AccumSpeed	6	1	62	0.0968	0.0968	0.0968
HRS	wave4_wave5	BranchScore	0	1	2569	0.4634	0.4634	0.4634
HRS	wave4_wave5	BranchScore	1	7	3661	0.4846	0.4321	0.451
HRS	wave4_wave5	BranchScore	2	12	3797	0.441	0.3709	0.3749
HRS	wave4_wave5	BranchScore	3	12	2627	0.3727	0.3045	0.3819
HRS	wave4_wave5	BranchScore	4	11	1364	0.2052	0.2066	0.2056
HRS	wave4_wave5	BranchScore	5	5	416	0.1463	0.1273	0.1434
HRS	wave4_wave5	BranchScore	6	1	62	0.1122	0.1122	0.1122
HRS	wave4_wave5	LockInScore	0	1	2569	0.7483	0.7483	0.7483
HRS	wave4_wave5	LockInScore	1	7	3661	1.2598	1.3816	1.3131
HRS	wave4_wave5	LockInScore	2	12	3797	1.8651	2.0708	1.8929
HRS	wave4_wave5	LockInScore	3	12	2627	2.4985	2.7636	2.4559
HRS	wave4_wave5	LockInScore	4	11	1364	3.3488	3.4401	3.4499
HRS	wave4_wave5	LockInScore	5	5	416	4.1146	4.2494	4.2158
HRS	wave4_wave5	LockInScore	6	1	62	4.5817	4.5817	4.5817

cohort	window	metric	disease count	n states	from n sum	score mean	score weighted mean	score median
HRS	wave5_wave6	AccumSpeed	0	1	1863	0.1938	0.1938	0.1938
HRS	wave5_wave6	AccumSpeed	1	6	2967	0.2433	0.2208	0.24
HRS	wave5_wave6	AccumSpeed	2	11	3284	0.2181	0.1973	0.2061
HRS	wave5_wave6	AccumSpeed	3	12	2414	0.1639	0.1611	0.1728
HRS	wave5_wave6	AccumSpeed	4	11	1274	0.1301	0.1303	0.1395
HRS	wave5_wave6	AccumSpeed	5	6	429	0.1094	0.1096	0.1168
HRS	wave5_wave6	AccumSpeed	6	1	58	0.069	0.069	0.069
HRS	wave5_wave6	BranchScore	0	1	1863	0.372	0.372	0.372
HRS	wave5_wave6	BranchScore	1	6	2967	0.456	0.4274	0.4509
HRS	wave5_wave6	BranchScore	2	11	3284	0.3876	0.3519	0.3782
HRS	wave5_wave6	BranchScore	3	12	2414	0.262	0.2684	0.2853
HRS	wave5_wave6	BranchScore	4	11	1274	0.1854	0.1904	0.2105
HRS	wave5_wave6	BranchScore	5	6	429	0.1424	0.1425	0.1548
HRS	wave5_wave6	BranchScore	6	1	58	0.0636	0.0636	0.0636
HRS	wave5_wave6	LockInScore	0	1	1863	0.7502	0.7502	0.7502
HRS	wave5_wave6	LockInScore	1	6	2967	1.2806	1.3855	1.2724
HRS	wave5_wave6	LockInScore	2	11	3284	1.9235	2.0545	1.9126
HRS	wave5_wave6	LockInScore	3	12	2414	2.6578	2.7753	2.6235
HRS	wave5_wave6	LockInScore	4	11	1274	3.4488	3.5001	3.3685
HRS	wave5_wave6	LockInScore	5	6	429	4.1372	4.1257	4.1862
HRS	wave5_wave6	LockInScore	6	1	58	4.4656	4.4656	4.4656

Table S8B. Count/support-adjusted top states and residual dynamic rankings.

cohort	window	metric	state id	state label	disease count	from n	score	count/support adjusted residual
CHARLS	wave1_wave2	AccumSpeed	00011000	lunge+hearte	2	55	0.2182	0.0742
CHARLS	wave1_wave2	AccumSpeed	00001000	hearte	1	331	0.1782	0.0375
CHARLS	wave1_wave2	AccumSpeed	00011001	lunge+hearte+arthre	3	72	0.125	0.0327
CHARLS	wave1_wave2	AccumSpeed	00001001	hearte+arthre	2	263	0.1331	0.0186
CHARLS	wave1_wave2	AccumSpeed	00010001	lunge+arthre	2	300	0.1267	0.0147
CHARLS	wave1_wave2	AccumSpeed	10000100	hibpe+stroke	2	78	0.141	0.0036
CHARLS	wave1_wave2	AccumSpeed	10000000	hibpe	1	1351	0.1155	0.0014
CHARLS	wave1_wave2	AccumSpeed	10001001	hibpe+hearte+arthre	3	240	0.0708	0.0013
CHARLS	wave1_wave2	AccumSpeed	11001001	hibpe+diabe+hearte+arthre	4	56	0.0714	0.0009
CHARLS	wave1_wave2	AccumSpeed	10001000	hibpe+hearte	2	303	0.1122	0.0004
CHARLS	wave1_wave2	BranchScore	00011000	lunge+hearte	2	55	0.352	0.128
CHARLS	wave1_wave2	BranchScore	00001000	hearte	1	331	0.3198	0.084
CHARLS	wave1_wave2	BranchScore	00001001	hearte+arthre	2	263	0.2155	0.0454
CHARLS	wave1_wave2	BranchScore	00011001	lunge+hearte+arthre	3	72	0.1657	0.0438
CHARLS	wave1_wave2	BranchScore	00010001	lunge+arthre	2	300	0.1857	0.0202
CHARLS	wave1_wave2	BranchScore	10000100	hibpe+stroke	2	78	0.2215	0.0095
CHARLS	wave1_wave2	BranchScore	10000000	hibpe	1	1351	0.1941	0.0071
CHARLS	wave1_wave2	BranchScore	10001001	hibpe+hearte+arthre	3	240	0.0859	0.0054
CHARLS	wave1_wave2	BranchScore	10010001	hibpe+lunge+arthre	3	96	0.115	0.0028
CHARLS	wave1_wave2	BranchScore	11001001	hibpe+diabe+hearte+arthre	4	56	0.0855	0.0013
CHARLS	wave1_wave2	LockInScore	11000000	hibpe+diabe	2	175	2.3789	0.176
CHARLS	wave1_wave2	LockInScore	10010001	hibpe+lunge+arthre	3	96	3.177	0.1736
CHARLS	wave1_wave2	LockInScore	00000100	stroke	1	51	1.3907	0.1247
CHARLS	wave1_wave2	LockInScore	00000010	psyche	1	75	1.416	0.1092
CHARLS	wave1_wave2	LockInScore	11001001	hibpe+diabe+hearte+arthre	4	56	4.0343	0.1026
CHARLS	wave1_wave2	LockInScore	11000001	hibpe+diabe+arthre	3	66	3.0435	0.08
CHARLS	wave1_wave2	LockInScore	10000001	hibpe+arthre	2	755	2.4296	0.0696
CHARLS	wave1_wave2	LockInScore	10001001	hibpe+hearte+arthre	3	240	3.168	0.0666
CHARLS	wave1_wave2	LockInScore	01000000	diabe	1	206	1.4772	0.0623
CHARLS	wave1_wave2	LockInScore	00010000	lunge	1	481	1.5648	0.0589
CHARLS	wave2_wave3	AccumSpeed	00010000	lunge	1	462	0.4394	0.0758
CHARLS	wave2_wave3	AccumSpeed	11001000	hibpe+diabe+hearte	3	83	0.3373	0.0462
CHARLS	wave2_wave3	AccumSpeed	10000000	hibpe	1	1399	0.3402	0.0384
CHARLS	wave2_wave3	AccumSpeed	10001000	hibpe+hearte	2	314	0.3121	0.0354
CHARLS	wave2_wave3	AccumSpeed	11001001	hibpe+diabe+hearte+arthre	4	59	0.1695	0.032
CHARLS	wave2_wave3	AccumSpeed	10000001	hibpe+arthre	2	789	0.2484	0.0231
CHARLS	wave2_wave3	AccumSpeed	10001001	hibpe+hearte+arthre	3	261	0.249	0.0214
CHARLS	wave2_wave3	AccumSpeed	10010001	hibpe+lunge+arthre	3	107	0.2897	0.0126
CHARLS	wave2_wave3	AccumSpeed	00001001	hearte+arthre	2	290	0.2931	0.012
CHARLS	wave2_wave3	AccumSpeed	11000001	hibpe+diabe+arthre	3	85	0.2941	0.0043
CHARLS	wave2_wave3	BranchScore	00010000	lunge	1	462	0.8548	0.1431
CHARLS	wave2_wave3	BranchScore	11001000	hibpe+diabe+hearte	3	83	0.6252	0.1342
CHARLS	wave2_wave3	BranchScore	10000000	hibpe	1	1399	0.7003	0.0917
CHARLS	wave2_wave3	BranchScore	11000000	hibpe+diabe	2	182	0.634	0.0843
CHARLS	wave2_wave3	BranchScore	10001000	hibpe+hearte	2	314	0.5688	0.0697
CHARLS	wave2_wave3	BranchScore	10010001	hibpe+lunge+arthre	3	107	0.5177	0.0501
CHARLS	wave2_wave3	BranchScore	11001001	hibpe+diabe+hearte+arthre	4	59	0.2278	0.0485
CHARLS	wave2_wave3	BranchScore	11000001	hibpe+diabe+arthre	3	85	0.5327	0.0439
CHARLS	wave2_wave3	BranchScore	10000001	hibpe+arthre	2	789	0.4508	0.0373
CHARLS	wave2_wave3	BranchScore	10001001	hibpe+hearte+arthre	3	261	0.4207	0.0356
CHARLS	wave2_wave3	LockInScore	00011001	lunge+hearte+arthre	3	87	2.4902	0.3681

cohort	window	metric	state id	state label	disease count	from n	score	count/support adjusted residual
CHARLS	wave2_wave3	LockInScore	10011001	hibpe+lunge+hearte+arthre	4	69	3.5247	0.3169
CHARLS	wave2_wave3	LockInScore	00010001	lunge+arthre	2	294	2.0009	0.19
CHARLS	wave2_wave3	LockInScore	11000001	hibpe+diabe+arthre	3	85	2.265	0.147
CHARLS	wave2_wave3	LockInScore	01000000	diabe	1	214	1.0724	0.0769
CHARLS	wave2_wave3	LockInScore	11000000	hibpe+diabe	2	182	1.7918	0.066
CHARLS	wave2_wave3	LockInScore	00001000	hearte	1	324	1.1096	0.0406
CHARLS	wave2_wave3	LockInScore	00000010	psyche	1	65	0.8249	0.0396
CHARLS	wave2_wave3	LockInScore	10010000	hibpe+lunge	2	131	1.6959	0.0282
CHARLS	wave2_wave3	LockInScore	10000001	hibpe+arthre	2	789	2.0042	0.018
CHARLS	wave3_wave4	AccumSpeed	10011000	hibpe+lunge+hearte	3	76	0.6184	0.1592
CHARLS	wave3_wave4	AccumSpeed	11001001	hibpe+diabe+hearte+arthre	4	94	0.4787	0.0727
CHARLS	wave3_wave4	AccumSpeed	00001000	hearte	1	310	0.5839	0.0679
CHARLS	wave3_wave4	AccumSpeed	01000001	diabe+arthre	2	146	0.589	0.064
CHARLS	wave3_wave4	AccumSpeed	01000000	diabe	1	207	0.5942	0.0636
CHARLS	wave3_wave4	AccumSpeed	11000001	hibpe+diabe+arthre	3	145	0.4897	0.0538
CHARLS	wave3_wave4	AccumSpeed	00001001	hearte+arthre	2	323	0.5449	0.0487
CHARLS	wave3_wave4	AccumSpeed	10001000	hibpe+hearte	2	322	0.5373	0.041
CHARLS	wave3_wave4	AccumSpeed	10010000	hibpe+lunge	2	137	0.5474	0.0201
CHARLS	wave3_wave4	AccumSpeed	00000101	stroke+arthre	2	57	0.5789	0.02
CHARLS	wave3_wave4	BranchScore	10011000	hibpe+lunge+hearte	3	76	1.264	0.4012
CHARLS	wave3_wave4	BranchScore	01000001	diabe+arthre	2	146	1.3717	0.2334
CHARLS	wave3_wave4	BranchScore	00001000	hearte	1	310	1.3662	0.205
CHARLS	wave3_wave4	BranchScore	00001001	hearte+arthre	2	323	1.223	0.1256
CHARLS	wave3_wave4	BranchScore	11001001	hibpe+diabe+hearte+arthre	4	94	0.8625	0.1138
CHARLS	wave3_wave4	BranchScore	10000101	hibpe+stroke+arthre	3	57	0.9799	0.1025
CHARLS	wave3_wave4	BranchScore	01000000	diabe	1	207	1.284	0.102
CHARLS	wave3_wave4	BranchScore	10000000	hibpe	1	1261	1.187	0.0982
CHARLS	wave3_wave4	BranchScore	11000001	hibpe+diabe+arthre	3	145	0.922	0.0922
CHARLS	wave3_wave4	BranchScore	10001000	hibpe+hearte	2	322	1.1824	0.0848
CHARLS	wave3_wave4	LockInScore	00000010	psyche	1	60	0.968	0.3251
CHARLS	wave3_wave4	LockInScore	11001000	hibpe+diabe+hearte	3	99	1.9968	0.2587
CHARLS	wave3_wave4	LockInScore	00100000	cancre	1	56	0.8268	0.1952
CHARLS	wave3_wave4	LockInScore	10011001	hibpe+lunge+hearte+arthre	4	126	2.5109	0.19
CHARLS	wave3_wave4	LockInScore	00011001	lunge+hearte+arthre	3	126	1.8822	0.1041
CHARLS	wave3_wave4	LockInScore	10000001	hibpe+arthre	2	947	1.7467	0.101
CHARLS	wave3_wave4	LockInScore	00010000	lunge	1	382	1.0438	0.0937
CHARLS	wave3_wave4	LockInScore	10010001	hibpe+lunge+arthre	3	168	1.9138	0.0879
CHARLS	wave3_wave4	LockInScore	01000000	diabe	1	207	0.883	0.035
CHARLS	wave3_wave4	LockInScore	00000101	stroke+arthre	2	57	1.1973	0.0187
CHARLS	wave4_wave5	AccumSpeed	10001100	hibpe+hearte+stroke	3	78	0.3846	0.0981
CHARLS	wave4_wave5	AccumSpeed	00001000	hearte	1	390	0.4103	0.094
CHARLS	wave4_wave5	AccumSpeed	10011000	hibpe+lunge+hearte	3	114	0.3684	0.0938
CHARLS	wave4_wave5	AccumSpeed	10000100	hibpe+stroke	2	186	0.371	0.0708
CHARLS	wave4_wave5	AccumSpeed	10001101	hibpe+hearte+stroke+arthre	4	80	0.3	0.0588
CHARLS	wave4_wave5	AccumSpeed	10010000	hibpe+lunge	2	213	0.3427	0.0468
CHARLS	wave4_wave5	AccumSpeed	10010001	hibpe+lunge+arthre	3	258	0.2868	0.038
CHARLS	wave4_wave5	AccumSpeed	00010000	lunge	1	484	0.3409	0.0315
CHARLS	wave4_wave5	AccumSpeed	10011001	hibpe+lunge+hearte+arthre	4	169	0.2367	0.019
CHARLS	wave4_wave5	AccumSpeed	11000000	hibpe+diabe	2	340	0.3	0.0189
CHARLS	wave4_wave5	BranchScore	00001000	hearte	1	390	0.9486	0.2726
CHARLS	wave4_wave5	BranchScore	10011000	hibpe+lunge+hearte	3	114	0.7486	0.2383
CHARLS	wave4_wave5	BranchScore	10000100	hibpe+stroke	2	186	0.8389	0.2362
CHARLS	wave4_wave5	BranchScore	10001100	hibpe+hearte+stroke	3	78	0.6926	0.1577
CHARLS	wave4_wave5	BranchScore	10010000	hibpe+lunge	2	213	0.7073	0.1134
CHARLS	wave4_wave5	BranchScore	10010001	hibpe+lunge+arthre	3	258	0.56	0.1029
CHARLS	wave4_wave5	BranchScore	00010000	lunge	1	484	0.7424	0.0805
CHARLS	wave4_wave5	BranchScore	10001101	hibpe+hearte+stroke+arthre	4	80	0.4909	0.075
CHARLS	wave4_wave5	BranchScore	10011001	hibpe+lunge+hearte+arthre	4	169	0.4306	0.0633
CHARLS	wave4_wave5	BranchScore	11000000	hibpe+diabe	2	340	0.6159	0.0526
CHARLS	wave4_wave5	LockInScore	00100000	cancre	1	94	1.3919	0.3985
CHARLS	wave4_wave5	LockInScore	11001001	hibpe+diabe+hearte+arthre	4	137	3.5191	0.3687
CHARLS	wave4_wave5	LockInScore	11001000	hibpe+diabe+hearte	3	168	2.6942	0.2413
CHARLS	wave4_wave5	LockInScore	00000010	psyche	1	82	1.2032	0.2295
CHARLS	wave4_wave5	LockInScore	00011001	lunge+hearte+arthre	3	162	2.6428	0.1952
CHARLS	wave4_wave5	LockInScore	00000100	stroke	1	98	1.1535	0.1541
CHARLS	wave4_wave5	LockInScore	01000001	diabe+arthre	2	169	1.8887	0.1087
CHARLS	wave4_wave5	LockInScore	10001000	hibpe+hearte	2	416	2.0093	0.0988
CHARLS	wave4_wave5	LockInScore	11000001	hibpe+diabe+arthre	3	219	2.5747	0.0835
CHARLS	wave4_wave5	LockInScore	11000100	hibpe+diabe+stroke	3	54	2.3529	0.0632
ELSA	wave1_wave2	AccumSpeed	10000010	hibpe+psyche	2	119	0.2185	0.0626
ELSA	wave1_wave2	AccumSpeed	01000000	diabe	1	129	0.2558	0.062
ELSA	wave1_wave2	AccumSpeed	11000001	hibpe+diabe+arthre	3	148	0.1757	0.0588
ELSA	wave1_wave2	AccumSpeed	10100001	hibpe+cancre+arthre	3	73	0.1918	0.058
ELSA	wave1_wave2	AccumSpeed	10000011	hibpe+psyche+arthre	3	89	0.1685	0.0395
ELSA	wave1_wave2	AccumSpeed	11000000	hibpe+diabe	2	175	0.1829	0.0362
ELSA	wave1_wave2	AccumSpeed	00001000	hearte	1	264	0.2008	0.024
ELSA	wave1_wave2	AccumSpeed	10000000	hibpe	1	1108	0.1543	0.012

cohort	window	metric	state id	state label	disease count	from n	score	count/support adjusted residual
ELSA	wave1_wave2	AccumSpeed	00100001	cancre+arthre	2	92	0.1739	0.0119
ELSA	wave1_wave2	AccumSpeed	01000001	diabe+arthre	2	50	0.18	0.0035
ELSA	wave1_wave2	BranchScore	10000010	hibpe+psyche	2	119	0.4119	0.1544
ELSA	wave1_wave2	BranchScore	01000000	diabe	1	129	0.5068	0.154
ELSA	wave1_wave2	BranchScore	11000001	hibpe+diabe+arthre	3	148	0.304	0.1259
ELSA	wave1_wave2	BranchScore	00001000	hearte	1	264	0.4051	0.0861
ELSA	wave1_wave2	BranchScore	10100001	hibpe+cancre+arthre	3	73	0.2881	0.0768
ELSA	wave1_wave2	BranchScore	10000011	hibpe+psyche+arthre	3	89	0.2628	0.0607
ELSA	wave1_wave2	BranchScore	11000000	hibpe+diabe	2	175	0.2916	0.0523
ELSA	wave1_wave2	BranchScore	00100001	cancre+arthre	2	92	0.3131	0.0436
ELSA	wave1_wave2	BranchScore	10000000	hibpe	1	1108	0.2681	0.0169
ELSA	wave1_wave2	BranchScore	10000001	hibpe+arthre	2	672	0.183	0.0072
ELSA	wave1_wave2	LockInScore	10001001	hibpe+hearte+arthre	3	221	3.44	0.43
ELSA	wave1_wave2	LockInScore	10010001	hibpe+lunge+arthre	3	55	3.0298	0.2198
ELSA	wave1_wave2	LockInScore	00000011	psyche+arthre	2	94	2.1524	0.1556
ELSA	wave1_wave2	LockInScore	00100000	cancre	1	187	1.4514	0.1469
ELSA	wave1_wave2	LockInScore	10001000	hibpe+hearte	2	224	2.2247	0.1027
ELSA	wave1_wave2	LockInScore	00000010	psyche	1	206	1.4101	0.0916
ELSA	wave1_wave2	LockInScore	00001001	hearte+arthre	2	182	2.1691	0.0771
ELSA	wave1_wave2	LockInScore	00001000	hearte	1	264	1.4171	0.0627
ELSA	wave1_wave2	LockInScore	00100001	cancre+arthre	2	92	2.0361	0.0424
ELSA	wave1_wave2	LockInScore	10000100	hibpe+stroke	2	56	1.9497	0.0271
ELSA	wave2_wave3	AccumSpeed	10100000	hibpe+cancre	2	98	0.2245	0.0595
ELSA	wave2_wave3	AccumSpeed	10000011	hibpe+psyche+arthre	3	88	0.1818	0.0514
ELSA	wave2_wave3	AccumSpeed	00000010	psyche	1	207	0.2126	0.0422
ELSA	wave2_wave3	AccumSpeed	00010000	lunge	1	82	0.1951	0.0213
ELSA	wave2_wave3	AccumSpeed	00000001	arthre	1	910	0.1835	0.0187
ELSA	wave2_wave3	AccumSpeed	11000001	hibpe+diabe+arthre	3	137	0.146	0.0172
ELSA	wave2_wave3	AccumSpeed	10001000	hibpe+hearte	2	229	0.179	0.0172
ELSA	wave2_wave3	AccumSpeed	11000000	hibpe+diabe	2	166	0.1747	0.0117
ELSA	wave2_wave3	AccumSpeed	00001001	hearte+arthre	2	179	0.1732	0.0104
ELSA	wave2_wave3	AccumSpeed	01000000	diabe	1	117	0.1795	0.007
ELSA	wave2_wave3	BranchScore	00000010	psyche	1	207	0.4317	0.1269
ELSA	wave2_wave3	BranchScore	10000011	hibpe+psyche+arthre	3	88	0.3066	0.1117
ELSA	wave2_wave3	BranchScore	10100000	hibpe+cancre	2	98	0.3418	0.0789
ELSA	wave2_wave3	BranchScore	10001000	hibpe+hearte	2	229	0.3178	0.0526
ELSA	wave2_wave3	BranchScore	00000001	arthre	1	910	0.3537	0.0451
ELSA	wave2_wave3	BranchScore	11000001	hibpe+diabe+arthre	3	137	0.2287	0.0327
ELSA	wave2_wave3	BranchScore	00001001	hearte+arthre	2	179	0.2966	0.0321
ELSA	wave2_wave3	BranchScore	00010000	lunge	1	82	0.3229	0.0205
ELSA	wave2_wave3	BranchScore	01000000	diabe	1	117	0.3198	0.0166
ELSA	wave2_wave3	BranchScore	11000000	hibpe+diabe	2	166	0.2713	0.007
ELSA	wave2_wave3	LockInScore	10000100	hibpe+stroke	2	55	2.1379	0.2314
ELSA	wave2_wave3	LockInScore	00100001	cancre+arthre	2	93	2.1207	0.1602
ELSA	wave2_wave3	LockInScore	00001000	hearte	1	271	1.5269	0.1412
ELSA	wave2_wave3	LockInScore	00100000	cancre	1	213	1.4826	0.1219
ELSA	wave2_wave3	LockInScore	10000001	hibpe+arthre	2	641	2.2666	0.1057
ELSA	wave2_wave3	LockInScore	10001001	hibpe+hearte+arthre	3	215	2.9761	0.0528
ELSA	wave2_wave3	LockInScore	01000000	diabe	1	117	1.3466	0.048
ELSA	wave2_wave3	LockInScore	11000001	hibpe+diabe+arthre	3	137	2.9014	0.0248
ELSA	wave2_wave3	LockInScore	00000010	psyche	1	207	1.3795	0.0218
ELSA	wave2_wave3	LockInScore	00000000	Healthy	0	2489	0.796	0
ELSA	wave3_wave4	AccumSpeed	10000010	hibpe+psyche	2	101	0.2376	0.0816
ELSA	wave3_wave4	AccumSpeed	00100000	cancre	1	196	0.2296	0.0495
ELSA	wave3_wave4	AccumSpeed	11000001	hibpe+diabe+arthre	3	124	0.1935	0.046
ELSA	wave3_wave4	AccumSpeed	10000000	hibpe	1	878	0.2016	0.0284
ELSA	wave3_wave4	AccumSpeed	00010000	lunge	1	72	0.2083	0.0237
ELSA	wave3_wave4	AccumSpeed	00000011	psyche+arthre	2	112	0.1786	0.023
ELSA	wave3_wave4	AccumSpeed	10100000	hibpe+cancre	2	90	0.1778	0.0212
ELSA	wave3_wave4	AccumSpeed	00100001	cancre+arthre	2	91	0.1648	0.0083
ELSA	wave3_wave4	AccumSpeed	10000001	hibpe+arthre	2	567	0.1499	0.0018
ELSA	wave3_wave4	AccumSpeed	11001001	hibpe+diabe+hearte+arthre	4	53	0.0943	0
ELSA	wave3_wave4	BranchScore	10000010	hibpe+psyche	2	101	0.3833	0.1388
ELSA	wave3_wave4	BranchScore	00100000	cancre	1	196	0.4438	0.1269
ELSA	wave3_wave4	BranchScore	11000001	hibpe+diabe+arthre	3	124	0.3011	0.0754
ELSA	wave3_wave4	BranchScore	10000000	hibpe	1	878	0.3853	0.0605
ELSA	wave3_wave4	BranchScore	00000011	psyche+arthre	2	112	0.2949	0.0498
ELSA	wave3_wave4	BranchScore	00010000	lunge	1	72	0.3591	0.0474
ELSA	wave3_wave4	BranchScore	00100001	cancre+arthre	2	91	0.2816	0.0376
ELSA	wave3_wave4	BranchScore	10100000	hibpe+cancre	2	90	0.2702	0.0262
ELSA	wave3_wave4	BranchScore	10000001	hibpe+arthre	2	567	0.2541	0.0005
ELSA	wave3_wave4	BranchScore	11001001	hibpe+diabe+hearte+arthre	4	53	0.1214	-0
ELSA	wave3_wave4	LockInScore	00001001	hearte+arthre	2	158	2.2342	0.1855
ELSA	wave3_wave4	LockInScore	10100001	hibpe+cancre+arthre	3	84	2.8239	0.1513
ELSA	wave3_wave4	LockInScore	00100001	cancre+arthre	2	91	2.0858	0.1072
ELSA	wave3_wave4	LockInScore	00000010	psyche	1	194	1.4087	0.0827
ELSA	wave3_wave4	LockInScore	01000001	diabe+arthre	2	53	1.9884	0.0781

cohort	window	metric	state id	state label	disease count	from n	score	count/support adjusted residual
ELSA	wave3_wave4	LockInScore	10000100	hibpe+stroke	2	52	1.9735	0.0656
ELSA	wave3_wave4	LockInScore	01000000	diabe	1	111	1.314	0.0591
ELSA	wave3_wave4	LockInScore	11000001	hibpe+diabe+arthre	3	124	2.7693	0.0473
ELSA	wave3_wave4	LockInScore	00000001	arthre	1	782	1.5487	0.0445
ELSA	wave3_wave4	LockInScore	10000001	hibpe+arthre	2	567	2.2395	0.0275
ELSA	wave4_wave5	AccumSpeed	10000011	hibpe+psyche+arthre	3	89	0.2584	0.094
ELSA	wave4_wave5	AccumSpeed	01000000	diabe	1	98	0.2959	0.0755
ELSA	wave4_wave5	AccumSpeed	00100001	cancre+arthre	2	92	0.2283	0.0403
ELSA	wave4_wave5	AccumSpeed	10000010	hibpe+psyche	2	82	0.2195	0.0292
ELSA	wave4_wave5	AccumSpeed	10000000	hibpe	1	725	0.2083	0.0288
ELSA	wave4_wave5	AccumSpeed	00000010	psyche	1	159	0.239	0.0284
ELSA	wave4_wave5	AccumSpeed	10000001	hibpe+arthre	2	517	0.1721	0.0195
ELSA	wave4_wave5	AccumSpeed	00001000	hearte	1	231	0.2208	0.0178
ELSA	wave4_wave5	AccumSpeed	11001000	hibpe+diabe+hearte	3	52	0.1923	0.017
ELSA	wave4_wave5	AccumSpeed	00010000	lunge	1	67	0.2388	0.0107
ELSA	wave4_wave5	BranchScore	10000011	hibpe+psyche+arthre	3	89	0.4822	0.2227
ELSA	wave4_wave5	BranchScore	01000000	diabe	1	98	0.5956	0.1838
ELSA	wave4_wave5	BranchScore	00100001	cancre+arthre	2	92	0.3966	0.085
ELSA	wave4_wave5	BranchScore	00010000	lunge	1	67	0.4961	0.0704
ELSA	wave4_wave5	BranchScore	10000000	hibpe	1	725	0.4079	0.0697
ELSA	wave4_wave5	BranchScore	10000010	hibpe+psyche	2	82	0.3725	0.0567
ELSA	wave4_wave5	BranchScore	00000010	psyche	1	159	0.4423	0.0482
ELSA	wave4_wave5	BranchScore	10000001	hibpe+arthre	2	517	0.2804	0.0323
ELSA	wave4_wave5	BranchScore	10001000	hibpe+hearte	2	211	0.3099	0.0288
ELSA	wave4_wave5	BranchScore	00001000	hearte	1	231	0.4041	0.0238
ELSA	wave4_wave5	LockInScore	00000011	psyche+arthre	2	98	2.216	0.2972
ELSA	wave4_wave5	LockInScore	10001001	hibpe+hearte+arthre	3	215	2.9152	0.1956
ELSA	wave4_wave5	LockInScore	00010000	lunge	1	67	1.3484	0.1853
ELSA	wave4_wave5	LockInScore	00100000	cancre	1	175	1.418	0.1267
ELSA	wave4_wave5	LockInScore	10001000	hibpe+hearte	2	211	2.1361	0.1146
ELSA	wave4_wave5	LockInScore	11000001	hibpe+diabe+arthre	3	113	2.697	0.0636
ELSA	wave4_wave5	LockInScore	00000001	arthre	1	706	1.5325	0.0536
ELSA	wave4_wave5	LockInScore	10100001	hibpe+cancre+arthre	3	76	2.6331	0.0527
ELSA	wave4_wave5	LockInScore	10100000	hibpe+cancre	2	84	1.9295	0.0313
ELSA	wave4_wave5	LockInScore	00001001	hearte+arthre	2	161	2.0041	0.0188
HRS	wave1_wave2	AccumSpeed	11010001	hibpe+diabe+lunge+arthre	4	62	0.3226	0.1551
HRS	wave1_wave2	AccumSpeed	00000101	stroke+arthre	2	65	0.3538	0.0974
HRS	wave1_wave2	AccumSpeed	11000101	hibpe+diabe+stroke+arthre	4	62	0.2581	0.0906
HRS	wave1_wave2	AccumSpeed	00010001	lunge+arthre	2	139	0.3237	0.086
HRS	wave1_wave2	AccumSpeed	10000010	hibpe+psyche	2	213	0.3099	0.0827
HRS	wave1_wave2	AccumSpeed	10010000	hibpe+lunge	2	65	0.3231	0.0666
HRS	wave1_wave2	AccumSpeed	10010001	hibpe+lunge+arthre	3	158	0.2658	0.0624
HRS	wave1_wave2	AccumSpeed	11000010	hibpe+diabe+psyche	3	78	0.2821	0.0612
HRS	wave1_wave2	AccumSpeed	10010011	hibpe+lunge+psyche+arthre	4	82	0.2195	0.0589
HRS	wave1_wave2	AccumSpeed	00000100	stroke	1	52	0.3462	0.0451
HRS	wave1_wave2	BranchScore	11010001	hibpe+diabe+lunge+arthre	4	62	0.6061	0.3401
HRS	wave1_wave2	BranchScore	00010001	lunge+arthre	2	139	0.7072	0.2671
HRS	wave1_wave2	BranchScore	00000101	stroke+arthre	2	65	0.7147	0.242
HRS	wave1_wave2	BranchScore	11000101	hibpe+diabe+stroke+arthre	4	62	0.4342	0.1682
HRS	wave1_wave2	BranchScore	10000010	hibpe+psyche	2	213	0.5682	0.1465
HRS	wave1_wave2	BranchScore	00000100	stroke	1	52	0.6987	0.1282
HRS	wave1_wave2	BranchScore	10010001	hibpe+lunge+arthre	3	158	0.4772	0.1241
HRS	wave1_wave2	BranchScore	10010011	hibpe+lunge+psyche+arthre	4	82	0.3683	0.1143
HRS	wave1_wave2	BranchScore	10000100	hibpe+stroke	2	99	0.5337	0.0791
HRS	wave1_wave2	BranchScore	10001000	hibpe+hearte	2	318	0.482	0.0776
HRS	wave1_wave2	LockInScore	10001101	hibpe+hearte+stroke+arthre	4	102	4.1264	0.7534
HRS	wave1_wave2	LockInScore	10011001	hibpe+lunge+hearte+arthre	4	94	3.7379	0.3781
HRS	wave1_wave2	LockInScore	10100001	hibpe+cancre+arthre	3	333	3.0938	0.3746
HRS	wave1_wave2	LockInScore	10101001	hibpe+cancre+hearte+arthre	4	120	3.6954	0.2962
HRS	wave1_wave2	LockInScore	11100001	hibpe+diabe+cancre+arthre	4	142	3.7139	0.2875
HRS	wave1_wave2	LockInScore	00010000	lunge	1	81	1.2379	0.2395
HRS	wave1_wave2	LockInScore	00100000	cancre	1	236	1.3811	0.2101
HRS	wave1_wave2	LockInScore	00000011	psyche+arthre	2	368	2.233	0.1914
HRS	wave1_wave2	LockInScore	00100001	cancre+arthre	2	261	2.1199	0.1341
HRS	wave1_wave2	LockInScore	00001001	hearte+arthre	2	270	2.1072	0.1158
HRS	wave2_wave3	AccumSpeed	01000000	diabe	1	273	0.359	0.0985
HRS	wave2_wave3	AccumSpeed	10001011	hibpe+hearte+psyche+arthre	4	163	0.227	0.0842
HRS	wave2_wave3	AccumSpeed	00010011	lunge+psyche+arthre	3	54	0.2963	0.0686
HRS	wave2_wave3	AccumSpeed	10010001	hibpe+lunge+arthre	3	158	0.2658	0.0679
HRS	wave2_wave3	AccumSpeed	10010000	hibpe+lunge	2	56	0.3214	0.0651
HRS	wave2_wave3	AccumSpeed	10000100	hibpe+stroke	2	91	0.3077	0.0648
HRS	wave2_wave3	AccumSpeed	11010001	hibpe+diabe+lunge+arthre	4	56	0.2321	0.0596
HRS	wave2_wave3	AccumSpeed	00010000	lunge	1	70	0.3571	0.0587
HRS	wave2_wave3	AccumSpeed	10001000	hibpe+hearte	2	268	0.2649	0.0521
HRS	wave2_wave3	AccumSpeed	00001011	hearte+psyche+arthre	3	54	0.2778	0.0501
HRS	wave2_wave3	BranchScore	01000000	diabe	1	273	0.7519	0.2371
HRS	wave2_wave3	BranchScore	00010000	lunge	1	70	0.7883	0.201

cohort	window	metric	state id	state label	disease count	from n	score	count/support adjusted residual
HRS	wave2_wave3	BranchScore	10000100	hibpe+stroke	2	91	0.6043	0.1607
HRS	wave2_wave3	BranchScore	10001011	hibpe+hearte+psyche+arthre	4	163	0.3779	0.1595
HRS	wave2_wave3	BranchScore	00010011	lunge+psyche+arthre	3	54	0.5541	0.1526
HRS	wave2_wave3	BranchScore	10001000	hibpe+hearte	2	268	0.5161	0.1301
HRS	wave2_wave3	BranchScore	11010001	hibpe+diabe+lunge+arthre	4	56	0.3947	0.1196
HRS	wave2_wave3	BranchScore	00001011	hearte+psyche+arthre	3	54	0.5169	0.1155
HRS	wave2_wave3	BranchScore	10010001	hibpe+lunge+arthre	3	158	0.4398	0.0953
HRS	wave2_wave3	BranchScore	10010000	hibpe+lunge	2	56	0.5599	0.0907
HRS	wave2_wave3	LockInScore	10100011	hibpe+cancre+psyche+arthre	4	81	4.1054	0.7371
HRS	wave2_wave3	LockInScore	11001101	hibpe+diabe+hearte+stroke+arthre	5	64	4.4921	0.5921
HRS	wave2_wave3	LockInScore	11001111	hibpe+diabe+hearte+stroke+psyche+arthre	6	51	5.336	0.5567
HRS	wave2_wave3	LockInScore	11100001	hibpe+diabe+cancre+arthre	4	134	3.9229	0.4723
HRS	wave2_wave3	LockInScore	11001001	hibpe+diabe+hearte+arthre	4	322	4.0522	0.4578
HRS	wave2_wave3	LockInScore	11011001	hibpe+diabe+lunge+hearte+arthre	5	56	4.3305	0.4522
HRS	wave2_wave3	LockInScore	10101000	hibpe+cancre+hearte	3	56	2.7493	0.3127
HRS	wave2_wave3	LockInScore	10100000	hibpe+cancre	2	173	2.1024	0.2033
HRS	wave2_wave3	LockInScore	10000101	hibpe+stroke+arthre	3	135	2.7786	0.1986
HRS	wave2_wave3	LockInScore	00100001	cancre+arthre	2	242	2.1248	0.1707
HRS	wave3_wave4	AccumSpeed	00010001	lunge+arthre	2	95	0.3895	0.1126
HRS	wave3_wave4	AccumSpeed	10011001	hibpe+lunge+hearte+arthre	4	88	0.3068	0.1059
HRS	wave3_wave4	AccumSpeed	10011011	hibpe+lunge+hearte+psyche+arthre	5	70	0.2714	0.1041
HRS	wave3_wave4	AccumSpeed	00010000	lunge	1	51	0.4314	0.1021
HRS	wave3_wave4	AccumSpeed	10000101	hibpe+stroke+arthre	3	125	0.304	0.0885
HRS	wave3_wave4	AccumSpeed	01001001	diabe+hearte+arthre	3	56	0.3036	0.0662
HRS	wave3_wave4	AccumSpeed	00000010	psyche	1	221	0.3484	0.0592
HRS	wave3_wave4	AccumSpeed	10000100	hibpe+stroke	2	76	0.3421	0.0591
HRS	wave3_wave4	AccumSpeed	11100000	hibpe+diabe+cancre	3	62	0.2903	0.0557
HRS	wave3_wave4	AccumSpeed	11010011	hibpe+diabe+lunge+psyche+arthre	5	53	0.2264	0.0515
HRS	wave3_wave4	BranchScore	00010001	lunge+arthre	2	95	0.8487	0.3409
HRS	wave3_wave4	BranchScore	00010000	lunge	1	51	0.9518	0.3158
HRS	wave3_wave4	BranchScore	10011001	hibpe+lunge+hearte+arthre	4	88	0.5921	0.26
HRS	wave3_wave4	BranchScore	10000101	hibpe+stroke+arthre	3	125	0.5945	0.2264
HRS	wave3_wave4	BranchScore	10011011	hibpe+lunge+hearte+psyche+arthre	5	70	0.4372	0.1837
HRS	wave3_wave4	BranchScore	00000010	psyche	1	221	0.7136	0.1412
HRS	wave3_wave4	BranchScore	10000100	hibpe+stroke	2	76	0.6381	0.1207
HRS	wave3_wave4	BranchScore	01001001	diabe+hearte+arthre	3	56	0.507	0.1041
HRS	wave3_wave4	BranchScore	10001000	hibpe+hearte	2	241	0.5588	0.0915
HRS	wave3_wave4	BranchScore	11100000	hibpe+diabe+cancre	3	62	0.4872	0.0887
HRS	wave3_wave4	LockInScore	11001101	hibpe+diabe+hearte+stroke+arthre	5	67	4.3024	0.5159
HRS	wave3_wave4	LockInScore	11101001	hibpe+diabe+cancre+hearte+arthre	5	67	4.2693	0.4827
HRS	wave3_wave4	LockInScore	11000011	hibpe+diabe+psyche+arthre	4	201	3.6309	0.4366
HRS	wave3_wave4	LockInScore	10101001	hibpe+cancre+hearte+arthre	4	116	3.5073	0.4111
HRS	wave3_wave4	LockInScore	11001011	hibpe+diabe+hearte+psyche+arthre	5	129	4.1666	0.2636
HRS	wave3_wave4	LockInScore	00100001	cancre+arthre	2	219	2.0376	0.2033
HRS	wave3_wave4	LockInScore	00100000	cancre	1	198	1.311	0.2003
HRS	wave3_wave4	LockInScore	00001000	hearte	1	169	1.2304	0.148
HRS	wave3_wave4	LockInScore	00001011	hearte+psyche+arthre	3	53	2.4903	0.1451
HRS	wave3_wave4	LockInScore	00001001	hearte+arthre	2	226	1.9773	0.1374
HRS	wave4_wave5	AccumSpeed	10000100	hibpe+stroke	2	77	0.4026	0.1352
HRS	wave4_wave5	AccumSpeed	00010000	lunge	1	59	0.4068	0.089
HRS	wave4_wave5	AccumSpeed	11100000	hibpe+diabe+cancre	3	60	0.3167	0.0841
HRS	wave4_wave5	AccumSpeed	11010011	hibpe+diabe+lunge+psyche+arthre	5	57	0.193	0.0773
HRS	wave4_wave5	AccumSpeed	10010001	hibpe+lunge+arthre	3	136	0.2647	0.0604
HRS	wave4_wave5	AccumSpeed	10010000	hibpe+lunge	2	57	0.3333	0.0555
HRS	wave4_wave5	AccumSpeed	11001000	hibpe+diabe+hearte	3	140	0.25	0.0467
HRS	wave4_wave5	AccumSpeed	11000010	hibpe+diabe+psyche	3	95	0.2632	0.0464
HRS	wave4_wave5	AccumSpeed	10001000	hibpe+hearte	2	238	0.2731	0.0448
HRS	wave4_wave5	AccumSpeed	10010011	hibpe+lunge+psyche+arthre	4	72	0.1944	0.0335
HRS	wave4_wave5	BranchScore	10000100	hibpe+stroke	2	77	0.822	0.3376
HRS	wave4_wave5	BranchScore	10010001	hibpe+lunge+arthre	3	136	0.4892	0.1345
HRS	wave4_wave5	BranchScore	00010000	lunge	1	59	0.7172	0.1255
HRS	wave4_wave5	BranchScore	11001000	hibpe+diabe+hearte	3	140	0.4686	0.1157
HRS	wave4_wave5	BranchScore	11100000	hibpe+diabe+cancre	3	60	0.5155	0.1116
HRS	wave4_wave5	BranchScore	10001000	hibpe+hearte	2	238	0.5256	0.1092
HRS	wave4_wave5	BranchScore	11000010	hibpe+diabe+psyche	3	95	0.4767	0.1004
HRS	wave4_wave5	BranchScore	11010011	hibpe+diabe+lunge+psyche+arthre	5	57	0.2514	0.0986
HRS	wave4_wave5	BranchScore	00000010	psyche	1	280	0.587	0.0892
HRS	wave4_wave5	BranchScore	10010000	hibpe+lunge	2	57	0.5757	0.0732
HRS	wave4_wave5	LockInScore	11001011	hibpe+diabe+hearte+psyche+arthre	5	135	4.7701	0.4285
HRS	wave4_wave5	LockInScore	10101001	hibpe+cancre+hearte+arthre	4	107	3.7606	0.3871
HRS	wave4_wave5	LockInScore	11000101	hibpe+diabe+stroke+arthre	4	56	3.6088	0.3718
HRS	wave4_wave5	LockInScore	00001000	hearte	1	157	1.3415	0.3136
HRS	wave4_wave5	LockInScore	01000011	diabe+psyche+arthre	3	72	2.7521	0.2994
HRS	wave4_wave5	LockInScore	00100001	cancre+arthre	2	192	2.0834	0.2177
HRS	wave4_wave5	LockInScore	01000000	diabe	1	277	1.3131	0.1646
HRS	wave4_wave5	LockInScore	10001101	hibpe+hearte+stroke+arthre	4	75	3.4317	0.1333
HRS	wave4_wave5	LockInScore	11001001	hibpe+diabe+hearte+arthre	4	311	3.7257	0.1257

cohort	window	metric	state id	state label	disease count	from n	score	count/support adjusted residual
HRS	wave4_wave5	LockInScore	00001001	hearte+arthre	2	193	1.9843	0.1175
HRS	wave5_wave6	AccumSpeed	10001000	hibpe+hearte	2	194	0.2835	0.0678
HRS	wave5_wave6	AccumSpeed	11010001	hibpe+diabe+lunge+arthre	4	67	0.209	0.064
HRS	wave5_wave6	AccumSpeed	00010001	lunge+arthre	2	86	0.2907	0.058
HRS	wave5_wave6	AccumSpeed	11011001	hibpe+diabe+lunge+hearte+arthre	5	55	0.1636	0.0476
HRS	wave5_wave6	AccumSpeed	01000000	diabe	1	241	0.2863	0.0429
HRS	wave5_wave6	AccumSpeed	11000011	hibpe+diabe+psyche+arthre	4	227	0.1586	0.0391
HRS	wave5_wave6	AccumSpeed	00010011	lunge+psyche+arthre	3	61	0.2295	0.0357
HRS	wave5_wave6	AccumSpeed	11100001	hibpe+diabe+cancre+arthre	4	119	0.1681	0.0351
HRS	wave5_wave6	AccumSpeed	10001001	hibpe+hearte+arthre	3	460	0.1848	0.0332
HRS	wave5_wave6	AccumSpeed	10010001	hibpe+lunge+arthre	3	124	0.2097	0.0306
HRS	wave5_wave6	BranchScore	00010001	lunge+arthre	2	86	0.5484	0.1486
HRS	wave5_wave6	BranchScore	10001000	hibpe+hearte	2	194	0.5128	0.1361
HRS	wave5_wave6	BranchScore	11010001	hibpe+diabe+lunge+arthre	4	67	0.3065	0.0963
HRS	wave5_wave6	BranchScore	10000010	hibpe+psyche	2	161	0.4712	0.0892
HRS	wave5_wave6	BranchScore	01000000	diabe	1	241	0.5443	0.0863
HRS	wave5_wave6	BranchScore	11011001	hibpe+diabe+lunge+hearte+arthre	5	55	0.2291	0.0778
HRS	wave5_wave6	BranchScore	10010001	hibpe+lunge+arthre	3	124	0.3662	0.0736
HRS	wave5_wave6	BranchScore	11000011	hibpe+diabe+psyche+arthre	4	227	0.2442	0.0685
HRS	wave5_wave6	BranchScore	10001001	hibpe+hearte+arthre	3	460	0.3234	0.068
HRS	wave5_wave6	BranchScore	11100001	hibpe+diabe+cancre+arthre	4	119	0.2567	0.0627
HRS	wave5_wave6	LockInScore	10001101	hibpe+hearte+stroke+arthre	4	67	4.295	0.9061
HRS	wave5_wave6	LockInScore	11101001	hibpe+diabe+cancre+hearte+arthre	5	71	4.8083	0.6915
HRS	wave5_wave6	LockInScore	00001011	hearte+psyche+arthre	3	57	3.1267	0.6102
HRS	wave5_wave6	LockInScore	10101001	hibpe+cancre+hearte+arthre	4	113	3.9913	0.5197
HRS	wave5_wave6	LockInScore	11010011	hibpe+diabe+lunge+psyche+arthre	5	59	4.4379	0.3503
HRS	wave5_wave6	LockInScore	10101000	hibpe+cancre+hearte	3	52	2.7299	0.2277
HRS	wave5_wave6	LockInScore	11000001	hibpe+diabe+arthre	3	674	3.1045	0.1952
HRS	wave5_wave6	LockInScore	11001001	hibpe+diabe+hearte+arthre	4	278	3.8024	0.1876
HRS	wave5_wave6	LockInScore	00000010	psyche	1	221	1.3267	0.1269
HRS	wave5_wave6	LockInScore	00100001	cancre+arthre	2	165	2.0079	0.1188

Table S9. Top-10 Jaccard overlap between accumulation speed and branching.

cohort	window	top-10 accumulation/branching Jaccard	shared top-10 state IDs	n shared top-10
CHARLS	wave1_wave2	0.6667	00011000; 00001000; 00000100; 10000100; 01000000; 00000010; 00001001; 00000000	8
CHARLS	wave2_wave3	0.8182	00010000; 00000010; 01000000; 00001000; 10000000; 11001000; 01000001; 10001000; 00000000	9
CHARLS	wave3_wave4	0.8182	10011000; 01000000; 01000001; 00001000; 00000101; 10010000; 00001001; 10001000; 00010001	9
CHARLS	wave4_wave5	0.8182	00001000; 10001100; 10000100; 10011000; 00100001; 10010000; 00010000; 00000101; 01000000	9
ELSA	wave1_wave2	0.8182	01000000; 10000010; 00001000; 10100001; 00010000; 11000000; 11000001; 00000010; 00100001	9
ELSA	wave2_wave3	0.6667	10100000; 00000010; 00010000; 00000001; 10000011; 01000000; 10001000; 00001001	8
ELSA	wave3_wave4	0.8182	10000010; 00100000; 00010000; 10000000; 11000001; 00000011; 00000000; 00001000; 00100001	9
ELSA	wave4_wave5	0.8182	01000000; 10000011; 00000010; 00010000; 00100001; 00001000; 10000010; 10000000; 00000000	9
HRS	wave1_wave2	0.8182	00000101; 00000100; 00010001; 10010000; 11010001; 10000010; 00000010; 10000100; 10010001	9
HRS	wave2_wave3	0.8182	01000000; 00010000; 10010000; 10000100; 00010011; 00001000; 00001011; 10001000; 00000010	9
HRS	wave3_wave4	0.5385	00010000; 00010001; 00000010; 10000100; 10011001; 10000101; 01000000	7
HRS	wave4_wave5	0.8182	00010000; 10000100; 10010000; 11100000; 00000010; 10001000; 10010001; 10000010; 00100000	9
HRS	wave5_wave6	0.8182	00010001; 01000000; 10001000; 00001000; 00100000; 10000010; 01000001; 10000000; 00000010	9

Table S10. Stabilisation alpha sensitivity and random eligible-state benchmark.

cohort	window	alpha	scenario	n	L1 mean	L1 median	L1 q25	L1 q75
CHARLS	wave1_wave2	0.1	Random eligible states	30	0.0312	0.025	0.0099	0.0383
CHARLS	wave1_wave2	0.1	Top accumulation states	1	0.1237	0.1237	0.1237	0.1237
CHARLS	wave1_wave2	0.1	Top branching states	1	0.1237	0.1237	0.1237	0.1237
CHARLS	wave1_wave2	0.2	Random eligible states	30	0.0624	0.05	0.0198	0.0765
CHARLS	wave1_wave2	0.2	Top accumulation states	1	0.2474	0.2474	0.2474	0.2474
CHARLS	wave1_wave2	0.2	Top branching states	1	0.2474	0.2474	0.2474	0.2474
CHARLS	wave1_wave2	0.3	Random eligible states	30	0.0936	0.0751	0.0298	0.1148
CHARLS	wave1_wave2	0.3	Top accumulation states	1	0.3711	0.3711	0.3711	0.3711
CHARLS	wave1_wave2	0.3	Top branching states	1	0.3711	0.3711	0.3711	0.3711
CHARLS	wave2_wave3	0.1	Random eligible states	30	0.0387	0.0223	0.0119	0.0648

cohort	window	alpha	scenario	n	L1 mean	L1 median	L1 q25	L1 q75
CHARLS	wave2_wave3	0.1	Top accumulation states	1	0.1247	0.1247	0.1247	0.1247
CHARLS	wave2_wave3	0.1	Top branching states	1	0.1223	0.1223	0.1223	0.1223
CHARLS	wave2_wave3	0.2	Random eligible states	30	0.0774	0.0445	0.0237	0.1297
CHARLS	wave2_wave3	0.2	Top accumulation states	1	0.2493	0.2493	0.2493	0.2493
CHARLS	wave2_wave3	0.2	Top branching states	1	0.2446	0.2446	0.2446	0.2446
CHARLS	wave2_wave3	0.3	Random eligible states	30	0.1161	0.0668	0.0356	0.1945
CHARLS	wave2_wave3	0.3	Top accumulation states	1	0.374	0.374	0.374	0.374
CHARLS	wave2_wave3	0.3	Top branching states	1	0.3668	0.3668	0.3668	0.3668
CHARLS	wave3_wave4	0.1	Random eligible states	30	0.0416	0.0284	0.0183	0.0656
CHARLS	wave3_wave4	0.1	Top accumulation states	1	0.1091	0.1091	0.1091	0.1091
CHARLS	wave3_wave4	0.1	Top branching states	1	0.1044	0.1044	0.1044	0.1044
CHARLS	wave3_wave4	0.2	Random eligible states	30	0.0832	0.0567	0.0367	0.1312
CHARLS	wave3_wave4	0.2	Top accumulation states	1	0.2182	0.2182	0.2182	0.2182
CHARLS	wave3_wave4	0.2	Top branching states	1	0.2089	0.2089	0.2089	0.2089
CHARLS	wave3_wave4	0.3	Random eligible states	30	0.1248	0.0851	0.055	0.1967
CHARLS	wave3_wave4	0.3	Top accumulation states	1	0.3273	0.3273	0.3273	0.3273
CHARLS	wave3_wave4	0.3	Top branching states	1	0.3133	0.3133	0.3133	0.3133
CHARLS	wave4_wave5	0.1	Random eligible states	30	0.0222	0.0159	0.0088	0.0273
CHARLS	wave4_wave5	0.1	Top accumulation states	1	0.0927	0.0927	0.0927	0.0927
CHARLS	wave4_wave5	0.1	Top branching states	1	0.0889	0.0889	0.0889	0.0889
CHARLS	wave4_wave5	0.2	Random eligible states	30	0.0444	0.0318	0.0176	0.0547
CHARLS	wave4_wave5	0.2	Top accumulation states	1	0.1854	0.1854	0.1854	0.1854
CHARLS	wave4_wave5	0.2	Top branching states	1	0.1778	0.1778	0.1778	0.1778
CHARLS	wave4_wave5	0.3	Random eligible states	30	0.0666	0.0476	0.0264	0.082
CHARLS	wave4_wave5	0.3	Top accumulation states	1	0.2781	0.2781	0.2781	0.2781
CHARLS	wave4_wave5	0.3	Top branching states	1	0.2668	0.2668	0.2668	0.2668
ELSA	wave1_wave2	0.1	Random eligible states	30	0.0329	0.0307	0.0158	0.045
ELSA	wave1_wave2	0.1	Top accumulation states	1	0.0894	0.0894	0.0894	0.0894
ELSA	wave1_wave2	0.1	Top branching states	1	0.0894	0.0894	0.0894	0.0894
ELSA	wave1_wave2	0.2	Random eligible states	30	0.0658	0.0615	0.0316	0.0899
ELSA	wave1_wave2	0.2	Top accumulation states	1	0.1788	0.1788	0.1788	0.1788
ELSA	wave1_wave2	0.2	Top branching states	1	0.1789	0.1789	0.1789	0.1789
ELSA	wave1_wave2	0.3	Random eligible states	30	0.0986	0.0922	0.0474	0.1349
ELSA	wave1_wave2	0.3	Top accumulation states	1	0.2682	0.2682	0.2682	0.2682
ELSA	wave1_wave2	0.3	Top branching states	1	0.2683	0.2683	0.2683	0.2683
ELSA	wave2_wave3	0.1	Random eligible states	30	0.0377	0.0318	0.0151	0.0535
ELSA	wave2_wave3	0.1	Top accumulation states	1	0.0893	0.0893	0.0893	0.0893
ELSA	wave2_wave3	0.1	Top branching states	1	0.0899	0.0899	0.0899	0.0899
ELSA	wave2_wave3	0.2	Random eligible states	30	0.0754	0.0636	0.0302	0.1071
ELSA	wave2_wave3	0.2	Top accumulation states	1	0.1786	0.1786	0.1786	0.1786
ELSA	wave2_wave3	0.2	Top branching states	1	0.1798	0.1798	0.1798	0.1798
ELSA	wave2_wave3	0.3	Random eligible states	30	0.1131	0.0954	0.0452	0.1606
ELSA	wave2_wave3	0.3	Top accumulation states	1	0.268	0.268	0.268	0.268
ELSA	wave2_wave3	0.3	Top branching states	1	0.2696	0.2696	0.2696	0.2696
ELSA	wave3_wave4	0.1	Random eligible states	30	0.0392	0.0303	0.0198	0.0587
ELSA	wave3_wave4	0.1	Top accumulation states	1	0.0851	0.0851	0.0851	0.0851
ELSA	wave3_wave4	0.1	Top branching states	1	0.0861	0.0861	0.0861	0.0861
ELSA	wave3_wave4	0.2	Random eligible states	30	0.0785	0.0606	0.0397	0.1174
ELSA	wave3_wave4	0.2	Top accumulation states	1	0.1703	0.1703	0.1703	0.1703
ELSA	wave3_wave4	0.2	Top branching states	1	0.1723	0.1723	0.1723	0.1723
ELSA	wave3_wave4	0.3	Random eligible states	30	0.1177	0.0909	0.0595	0.1761
ELSA	wave3_wave4	0.3	Top accumulation states	1	0.2554	0.2554	0.2554	0.2554
ELSA	wave3_wave4	0.3	Top branching states	1	0.2584	0.2584	0.2584	0.2584
ELSA	wave4_wave5	0.1	Random eligible states	30	0.0362	0.034	0.0167	0.0475
ELSA	wave4_wave5	0.1	Top accumulation states	1	0.0791	0.0791	0.0791	0.0791
ELSA	wave4_wave5	0.1	Top branching states	1	0.08	0.08	0.08	0.08
ELSA	wave4_wave5	0.2	Random eligible states	30	0.0723	0.0681	0.0334	0.095
ELSA	wave4_wave5	0.2	Top accumulation states	1	0.1582	0.1582	0.1582	0.1582
ELSA	wave4_wave5	0.2	Top branching states	1	0.16	0.16	0.16	0.16
ELSA	wave4_wave5	0.3	Random eligible states	30	0.1085	0.1021	0.0501	0.1425
ELSA	wave4_wave5	0.3	Top accumulation states	1	0.2374	0.2374	0.2374	0.2374
ELSA	wave4_wave5	0.3	Top branching states	1	0.24	0.24	0.24	0.24
HRS	wave1_wave2	0.1	Random eligible states	30	0.0143	0.0097	0.0069	0.0215
HRS	wave1_wave2	0.1	Top accumulation states	1	0.0233	0.0233	0.0233	0.0233
HRS	wave1_wave2	0.1	Top branching states	1	0.0245	0.0245	0.0245	0.0245
HRS	wave1_wave2	0.2	Random eligible states	30	0.0285	0.0194	0.0137	0.0431
HRS	wave1_wave2	0.2	Top accumulation states	1	0.0466	0.0466	0.0466	0.0466
HRS	wave1_wave2	0.2	Top branching states	1	0.049	0.049	0.049	0.049
HRS	wave1_wave2	0.3	Random eligible states	30	0.0428	0.0291	0.0206	0.0646
HRS	wave1_wave2	0.3	Top accumulation states	1	0.0699	0.0699	0.0699	0.0699
HRS	wave1_wave2	0.3	Top branching states	1	0.0735	0.0735	0.0735	0.0735
HRS	wave2_wave3	0.1	Random eligible states	30	0.011	0.0092	0.0065	0.0137
HRS	wave2_wave3	0.1	Top accumulation states	1	0.0222	0.0222	0.0222	0.0222
HRS	wave2_wave3	0.1	Top branching states	1	0.0237	0.0237	0.0237	0.0237
HRS	wave2_wave3	0.2	Random eligible states	30	0.0219	0.0184	0.013	0.0273
HRS	wave2_wave3	0.2	Top accumulation states	1	0.0445	0.0445	0.0445	0.0445
HRS	wave2_wave3	0.2	Top branching states	1	0.0474	0.0474	0.0474	0.0474

cohort	window	alpha	scenario	n	L1 mean	L1 median	L1 q25	L1 q75
HRS	wave2_wave3	0.3	Random eligible states	30	0.0329	0.0276	0.0195	0.041
HRS	wave2_wave3	0.3	Top accumulation states	1	0.0667	0.0667	0.0667	0.0667
HRS	wave2_wave3	0.3	Top branching states	1	0.0711	0.0711	0.0711	0.0711
HRS	wave3_wave4	0.1	Random eligible states	30	0.0155	0.0133	0.0086	0.0227
HRS	wave3_wave4	0.1	Top accumulation states	1	0.0214	0.0214	0.0214	0.0214
HRS	wave3_wave4	0.1	Top branching states	1	0.0249	0.0249	0.0249	0.0249
HRS	wave3_wave4	0.2	Random eligible states	30	0.0309	0.0266	0.0171	0.0454
HRS	wave3_wave4	0.2	Top accumulation states	1	0.0428	0.0428	0.0428	0.0428
HRS	wave3_wave4	0.2	Top branching states	1	0.0497	0.0497	0.0497	0.0497
HRS	wave3_wave4	0.3	Random eligible states	30	0.0464	0.04	0.0257	0.0681
HRS	wave3_wave4	0.3	Top accumulation states	1	0.0642	0.0642	0.0642	0.0642
HRS	wave3_wave4	0.3	Top branching states	1	0.0746	0.0746	0.0746	0.0746
HRS	wave4_wave5	0.1	Random eligible states	30	0.0187	0.0156	0.0095	0.0231
HRS	wave4_wave5	0.1	Top accumulation states	1	0.0517	0.0517	0.0517	0.0517
HRS	wave4_wave5	0.1	Top branching states	1	0.055	0.055	0.055	0.055
HRS	wave4_wave5	0.2	Random eligible states	30	0.0374	0.0312	0.0191	0.0462
HRS	wave4_wave5	0.2	Top accumulation states	1	0.1033	0.1033	0.1033	0.1033
HRS	wave4_wave5	0.2	Top branching states	1	0.1101	0.1101	0.1101	0.1101
HRS	wave4_wave5	0.3	Random eligible states	30	0.0561	0.0468	0.0286	0.0693
HRS	wave4_wave5	0.3	Top accumulation states	1	0.155	0.155	0.155	0.155
HRS	wave4_wave5	0.3	Top branching states	1	0.1651	0.1651	0.1651	0.1651
HRS	wave5_wave6	0.1	Random eligible states	30	0.0204	0.0188	0.011	0.0267
HRS	wave5_wave6	0.1	Top accumulation states	1	0.048	0.048	0.048	0.048
HRS	wave5_wave6	0.1	Top branching states	1	0.0498	0.0498	0.0498	0.0498
HRS	wave5_wave6	0.2	Random eligible states	30	0.0408	0.0377	0.0221	0.0534
HRS	wave5_wave6	0.2	Top accumulation states	1	0.096	0.096	0.096	0.096
HRS	wave5_wave6	0.2	Top branching states	1	0.0996	0.0996	0.0996	0.0996
HRS	wave5_wave6	0.3	Random eligible states	30	0.0612	0.0565	0.0331	0.08
HRS	wave5_wave6	0.3	Top accumulation states	1	0.144	0.144	0.144	0.144
HRS	wave5_wave6	0.3	Top branching states	1	0.1494	0.1494	0.1494	0.1494

Table S11. BranchScore component rankings among top-ranked branching states by cohort and transition window.

The table decomposes BranchScore into outgoing transition entropy and downstream disease-count variance. Only top-10 BranchScore states within each cohort-window are shown. Abbreviated state labels follow the analytic disease codes used in the primary tables.

Cohort	Window	Branch rank	State label	Disease count	From n	Entropy	Var. downstream count	BranchScore	Entropy rank	Variance rank
CHARLS	wave1_wave2	1	lunge+hearte	2	55	0.774	0.207	0.352	1	2
CHARLS	wave1_wave2	2	hearte	1	331	0.725	0.195	0.320	2	3
CHARLS	wave1_wave2	3	stroke	1	51	0.546	0.250	0.273	7	1
CHARLS	wave1_wave2	4	diabe	1	206	0.602	0.150	0.233	3	5
CHARLS	wave1_wave2	5	hibpe+stroke	2	78	0.533	0.172	0.221	9	4
CHARLS	wave1_wave2	6	Healthy	0	6037	0.575	0.148	0.221	5	6
CHARLS	wave1_wave2	7	hearte+arthre	2	263	0.580	0.138	0.216	4	8
CHARLS	wave1_wave2	8	psyche	1	75	0.568	0.142	0.214	6	7
CHARLS	wave1_wave2	9	hibpe	1	1351	0.544	0.127	0.194	8	11
CHARLS	wave1_wave2	10	lunge	1	481	0.522	0.134	0.191	10	9
CHARLS	wave2_wave3	1	lunge	1	462	1.334	0.411	0.855	1	1
CHARLS	wave2_wave3	2	psyche	1	65	1.291	0.335	0.747	2	8
CHARLS	wave2_wave3	3	diabe	1	214	1.190	0.365	0.719	4	3
CHARLS	wave2_wave3	4	hearte	1	324	1.175	0.362	0.707	5	4
CHARLS	wave2_wave3	5	hibpe	1	1399	1.198	0.342	0.700	3	7
CHARLS	wave2_wave3	6	Healthy	0	5739	1.122	0.335	0.649	6	9
CHARLS	wave2_wave3	7	hibpe+diabe	2	182	1.063	0.356	0.634	8	5
CHARLS	wave2_wave3	8	hibpe+diabe+hearte	3	83	1.030	0.368	0.625	9	2
CHARLS	wave2_wave3	9	hibpe+hearte	2	314	1.078	0.278	0.569	7	12
CHARLS	wave2_wave3	10	diabe+arthre	2	112	0.999	0.304	0.551	13	10
CHARLS	wave3_wave4	1	diabe+arthre	2	146	1.615	0.722	1.372	6	1
CHARLS	wave3_wave4	2	hearte	1	310	1.758	0.604	1.366	1	4
CHARLS	wave3_wave4	3	diabe	1	207	1.673	0.589	1.284	2	5
CHARLS	wave3_wave4	4	hibpe+lunge+hearte	3	76	1.626	0.604	1.264	5	3
CHARLS	wave3_wave4	5	stroke+arthre	2	57	1.541	0.665	1.257	10	2
CHARLS	wave3_wave4	6	hearte+arthre	2	323	1.611	0.576	1.223	7	6
CHARLS	wave3_wave4	7	hibpe	1	1261	1.599	0.551	1.187	8	8
CHARLS	wave3_wave4	8	hibpe+lunge	2	137	1.633	0.525	1.183	3	12
CHARLS	wave3_wave4	9	hibpe+hearte	2	322	1.627	0.528	1.182	4	11
CHARLS	wave3_wave4	10	lunge+arthre	2	414	1.524	0.535	1.114	11	10
CHARLS	wave4_wave5	1	hearte	1	390	1.330	0.509	0.949	1	1
CHARLS	wave4_wave5	2	hibpe+stroke	2	186	1.197	0.491	0.839	2	2
CHARLS	wave4_wave5	3	hibpe+lunge+hearte	3	114	1.103	0.461	0.749	8	3
CHARLS	wave4_wave5	4	lunge	1	484	1.189	0.390	0.742	3	6
CHARLS	wave4_wave5	5	hibpe+lunge	2	213	1.140	0.385	0.707	6	8

CHARLS	wave4_wave5	6	cancre+arthre	2	51	1.139	0.385	0.707	7	7
CHARLS	wave4_wave5	7	diabe	1	328	1.174	0.351	0.696	4	11
CHARLS	wave4_wave5	8	hibpe+hearte+stroke	3	78	1.147	0.365	0.693	5	9
CHARLS	wave4_wave5	9	stroke+arthre	2	86	1.070	0.410	0.685	9	5
CHARLS	wave4_wave5	10	psyche	1	82	0.990	0.421	0.643	17	4
ELSA	wave1_wave2	1	diabe	1	129	0.904	0.314	0.507	1	1
ELSA	wave1_wave2	2	hibpe+psyche	2	119	0.876	0.221	0.412	2	4
ELSA	wave1_wave2	3	hearte	1	264	0.808	0.251	0.405	3	3
ELSA	wave1_wave2	4	psyche	1	206	0.733	0.193	0.322	5	6
ELSA	wave1_wave2	5	cancre+arthre	2	92	0.576	0.296	0.313	15	2
ELSA	wave1_wave2	6	lunge	1	92	0.788	0.151	0.306	4	13
ELSA	wave1_wave2	7	hibpe+diabe+arthre	3	148	0.682	0.199	0.304	10	5
ELSA	wave1_wave2	8	Healthy	0	2525	0.720	0.165	0.292	7	9
ELSA	wave1_wave2	9	hibpe+diabe	2	175	0.727	0.161	0.292	6	11
ELSA	wave1_wave2	10	hibpe+cancre+arthre	3	73	0.675	0.182	0.288	11	7
ELSA	wave2_wave3	1	psyche	1	207	0.840	0.264	0.432	1	1
ELSA	wave2_wave3	2	arthre	1	910	0.790	0.200	0.354	3	3
ELSA	wave2_wave3	3	hibpe+cancre	2	98	0.819	0.174	0.342	2	9
ELSA	wave2_wave3	4	lunge	1	82	0.758	0.181	0.323	5	7
ELSA	wave2_wave3	5	diabe	1	117	0.718	0.199	0.320	7	4
ELSA	wave2_wave3	6	hibpe+hearte	2	229	0.764	0.173	0.318	4	10
ELSA	wave2_wave3	7	hibpe+psyche+arthre	3	88	0.598	0.262	0.307	13	2
ELSA	wave2_wave3	8	Healthy	0	2489	0.713	0.176	0.299	8	8
ELSA	wave2_wave3	9	hearte+arthre	2	179	0.729	0.166	0.297	6	12
ELSA	wave2_wave3	10	hibpe	1	1017	0.697	0.171	0.288	9	11
ELSA	wave3_wave4	1	cancre	1	196	0.910	0.238	0.444	1	2
ELSA	wave3_wave4	2	hibpe	1	878	0.825	0.218	0.385	3	4
ELSA	wave3_wave4	3	hibpe+psyche	2	101	0.855	0.201	0.383	2	5
ELSA	wave3_wave4	4	lunge	1	72	0.818	0.193	0.359	4	6
ELSA	wave3_wave4	5	Healthy	0	2142	0.782	0.188	0.339	5	7
ELSA	wave3_wave4	6	hibpe+diabe+arthre	3	124	0.599	0.253	0.301	16	1
ELSA	wave3_wave4	7	psyche+arthre	2	112	0.690	0.182	0.295	8	8
ELSA	wave3_wave4	8	cancre+arthre	2	91	0.593	0.226	0.282	17	3
ELSA	wave3_wave4	9	hearte	1	253	0.708	0.154	0.278	6	12
ELSA	wave3_wave4	10	psyche	1	194	0.681	0.165	0.277	9	9
ELSA	wave4_wave5	1	diabe	1	98	1.106	0.290	0.596	1	2
ELSA	wave4_wave5	2	lunge	1	67	0.716	0.480	0.496	9	1

ELSA	wave4_wave5	3	hibpe+psyche+arthre	3	89	0.947	0.259	0.482	2	3
ELSA	wave4_wave5	4	psyche	1	159	0.872	0.257	0.442	4	4
ELSA	wave4_wave5	5	hibpe	1	725	0.854	0.228	0.408	5	5
ELSA	wave4_wave5	6	hearte	1	231	0.889	0.207	0.404	3	7
ELSA	wave4_wave5	7	cancre+arthre	2	92	0.846	0.220	0.397	6	6
ELSA	wave4_wave5	8	hibpe+psyche	2	82	0.842	0.196	0.372	7	9
ELSA	wave4_wave5	9	Healthy	0	1664	0.771	0.189	0.335	8	10
ELSA	wave4_wave5	10	hibpe+hearte	2	211	0.690	0.201	0.310	11	8
HRS	wave1_wave2	1	stroke+arthre	2	65	1.205	0.352	0.715	1	2
HRS	wave1_wave2	2	lunge+arthre	2	139	1.023	0.478	0.707	6	1
HRS	wave1_wave2	3	stroke	1	52	1.195	0.342	0.699	2	5
HRS	wave1_wave2	4	hibpe+diabe+lunge+arthre	4	62	1.028	0.348	0.606	5	3
HRS	wave1_wave2	5	psyche	1	317	0.966	0.347	0.569	7	4
HRS	wave1_wave2	6	hibpe+psyche	2	213	1.133	0.251	0.568	3	14
HRS	wave1_wave2	7	hibpe+stroke	2	99	0.916	0.340	0.534	10	6
HRS	wave1_wave2	8	hibpe+lunge	2	65	1.049	0.249	0.524	4	16
HRS	wave1_wave2	9	hibpe+hearte	2	318	0.925	0.272	0.482	9	10
HRS	wave1_wave2	10	hibpe+lunge+arthre	3	158	0.963	0.246	0.477	8	17
HRS	wave2_wave3	1	lunge	1	70	1.130	0.487	0.788	1	1
HRS	wave2_wave3	2	diabe	1	273	1.103	0.465	0.752	2	2
HRS	wave2_wave3	3	hearte	1	209	1.024	0.360	0.614	5	3
HRS	wave2_wave3	4	hibpe+stroke	2	91	1.102	0.301	0.604	3	6
HRS	wave2_wave3	5	hibpe+lunge	2	56	1.041	0.290	0.560	4	7
HRS	wave2_wave3	6	lunge+psyche+arthre	3	54	0.980	0.320	0.554	8	5
HRS	wave2_wave3	7	hearte+psyche+arthre	3	54	0.986	0.275	0.517	7	9
HRS	wave2_wave3	8	hibpe+hearte	2	268	1.009	0.262	0.516	6	11
HRS	wave2_wave3	9	lunge+arthre	2	113	0.870	0.332	0.502	12	4
HRS	wave2_wave3	10	psyche	1	266	0.955	0.269	0.495	9	10
HRS	wave3_wave4	1	lunge	1	51	1.273	0.559	0.952	1	1
HRS	wave3_wave4	2	lunge+arthre	2	95	1.163	0.533	0.849	3	2
HRS	wave3_wave4	3	psyche	1	221	1.232	0.336	0.714	2	5
HRS	wave3_wave4	4	hibpe+stroke	2	76	1.157	0.304	0.638	4	10
HRS	wave3_wave4	5	hibpe+stroke+arthre	3	125	1.072	0.308	0.595	5	9
HRS	wave3_wave4	6	hibpe+lunge+hearte+arthre	4	88	1.002	0.349	0.592	8	3
HRS	wave3_wave4	7	diabe	1	215	0.969	0.349	0.572	12	4
HRS	wave3_wave4	8	hibpe+hearte	2	241	0.973	0.330	0.559	11	6
HRS	wave3_wave4	9	Healthy	0	2195	1.000	0.311	0.558	9	8
HRS	wave3_wave4	10	hearte	1	169	1.016	0.293	0.549	7	11

HRS	wave4_wave5	1	hibpe+stroke	2	77	1.265	0.422	0.822	1	1
HRS	wave4_wave5	2	lunge	1	59	1.168	0.377	0.717	2	3
HRS	wave4_wave5	3	psyche	1	280	0.949	0.383	0.587	7	2
HRS	wave4_wave5	4	hibpe+lunge	2	57	1.135	0.257	0.576	3	13
HRS	wave4_wave5	5	hibpe+hearte	2	238	1.004	0.274	0.526	4	10
HRS	wave4_wave5	6	hibpe+diabe+cancre	3	60	0.969	0.283	0.515	6	8
HRS	wave4_wave5	7	hibpe+psyche	2	198	0.926	0.285	0.494	8	7
HRS	wave4_wave5	8	cancre	1	206	0.926	0.281	0.490	9	9
HRS	wave4_wave5	9	diabe+arthre	2	190	0.908	0.291	0.490	13	6
HRS	wave4_wave5	10	hibpe+lunge+arthre	3	136	0.972	0.253	0.489	5	14
HRS	wave5_wave6	1	lunge+arthre	2	86	0.966	0.322	0.548	4	1
HRS	wave5_wave6	2	diabe	1	241	1.001	0.296	0.544	3	2
HRS	wave5_wave6	3	hibpe+hearte	2	194	1.016	0.255	0.513	2	4
HRS	wave5_wave6	4	hearte	1	131	1.041	0.233	0.503	1	10
HRS	wave5_wave6	5	hibpe+psyche	2	161	0.901	0.274	0.471	6	3
HRS	wave5_wave6	6	cancre	1	171	0.938	0.238	0.457	5	7
HRS	wave5_wave6	7	hibpe	1	1168	0.889	0.250	0.445	7	5
HRS	wave5_wave6	8	psyche	1	221	0.863	0.236	0.419	8	9
HRS	wave5_wave6	9	diabe+arthre	2	174	0.814	0.229	0.390	9	12
HRS	wave5_wave6	10	cancre+arthre	2	165	0.793	0.236	0.386	13	8

Table S12. Prevalence-adjusted enrichment of multimorbidity profiles by cohort and transition window.

Observed profile counts were compared with expected counts under an independent-prevalence assumption using marginal disease prevalence at the starting wave. The table shows the 10 most enriched eligible profiles in each cohort-window. O/E, observed-to-expected ratio; FDR, false discovery rate.

Cohort	Window	State label	Disease count	Observed n	Expected n	O/E ratio	FDR
CHARLS	wave1_wave2	hibpe+diabe+hearte+arthre	4	70	9.14	7.66	3.09e-36
CHARLS	wave1_wave2	hibpe+lunge+hearte+arthre	4	73	15.05	4.85	5.15e-26
CHARLS	wave1_wave2	hibpe+hearte+arthre	3	284	140.52	2.02	5.15e-26
CHARLS	wave1_wave2	hibpe+diabe+hearte	3	80	19.10	4.19	1.13e-24
CHARLS	wave1_wave2	lunge+hearte+arthre	3	85	43.05	1.97	3.72e-08
CHARLS	wave1_wave2	hibpe+diabe	2	206	138.14	1.49	1.13e-07
CHARLS	wave1_wave2	hibpe+stroke	2	106	60.78	1.74	2.32e-07
CHARLS	wave1_wave2	hibpe+lunge+hearte	3	59	31.44	1.88	1.63e-05
CHARLS	wave1_wave2	hibpe+hearte	2	368	293.58	1.25	2.70e-05
CHARLS	wave1_wave2	hibpe+stroke+arthre	3	51	29.09	1.75	2.64e-04
CHARLS	wave2_wave3	hibpe+diabe+hearte+arthre	4	75	13.62	5.51	1.16e-29

CHARLS	wave2_wave3	hibpe+lunge+hearte+arthre	4	84	20.15	4.17	1.45e-25
CHARLS	wave2_wave3	hibpe+diabe+hearte	3	98	26.75	3.66	1.45e-25
CHARLS	wave2_wave3	hibpe+hearte+arthre	3	304	170.14	1.79	4.61e-20
CHARLS	wave2_wave3	lunge+hearte+arthre	3	103	51.93	1.98	9.58e-10
CHARLS	wave2_wave3	hibpe+diabe	2	233	171.01	1.36	1.12e-05
CHARLS	wave2_wave3	hibpe+lunge+hearte	3	66	39.58	1.67	2.04e-04
CHARLS	wave2_wave3	hibpe+stroke+arthre	3	57	34.96	1.63	8.87e-04
CHARLS	wave2_wave3	hibpe+hearte	2	395	334.26	1.18	0.001
CHARLS	wave2_wave3	hibpe+stroke	2	96	68.68	1.40	0.002
CHARLS	wave3_wave4	hibpe+lunge+hearte+arthre	4	160	46.59	3.43	2.18e-37
CHARLS	wave3_wave4	hibpe+diabe+hearte+arthre	4	108	30.39	3.55	8.15e-27
CHARLS	wave3_wave4	hibpe+diabe+hearte	3	116	40.70	2.85	3.02e-21
CHARLS	wave3_wave4	hibpe+hearte+stroke+arthre	4	55	11.35	4.85	6.35e-20
CHARLS	wave3_wave4	hibpe+hearte+arthre	3	411	287.69	1.43	1.33e-11
CHARLS	wave3_wave4	lunge+hearte+arthre	3	160	94.44	1.69	1.66e-09
CHARLS	wave3_wave4	hibpe+lunge+hearte	3	94	62.41	1.51	3.52e-04
CHARLS	wave3_wave4	hibpe+diabe	2	227	190.92	1.19	0.016
CHARLS	wave3_wave4	hibpe+stroke+arthre	3	70	53.24	1.31	0.036
CHARLS	wave3_wave4	hibpe+diabe+arthre	3	169	142.54	1.19	0.036
CHARLS	wave4_wave5	hibpe+diabe+lunge+hearte+arthre	5	75	10.10	7.42	1.04e-37
CHARLS	wave4_wave5	hibpe+lunge+hearte+arthre	4	198	68.58	2.89	4.23e-36
CHARLS	wave4_wave5	hibpe+diabe+hearte+arthre	4	151	55.26	2.73	1.71e-25
CHARLS	wave4_wave5	hibpe+diabe+hearte	3	199	86.82	2.29	2.73e-24
CHARLS	wave4_wave5	hibpe+hearte+stroke+arthre	4	99	29.14	3.40	1.61e-23
CHARLS	wave4_wave5	lunge+hearte+arthre	3	190	110.63	1.72	1.88e-11
CHARLS	wave4_wave5	hibpe+hearte+stroke	3	96	45.79	2.10	2.50e-10
CHARLS	wave4_wave5	hibpe+diabe+stroke	3	67	27.45	2.44	4.44e-10
CHARLS	wave4_wave5	hibpe+hearte+arthre	3	481	375.06	1.28	2.03e-07
CHARLS	wave4_wave5	hibpe+diabe+lunge+arthre	4	77	41.11	1.87	1.02e-06
ELSA	wave1_wave2	hibpe+diabe+hearte+arthre	4	62	23.81	2.60	9.44e-10
ELSA	wave1_wave2	hibpe+diabe+arthre	3	173	103.26	1.68	1.80e-09
ELSA	wave1_wave2	hibpe+hearte+arthre	3	275	198.16	1.39	6.71e-07
ELSA	wave1_wave2	hibpe+diabe+hearte	3	54	40.74	1.33	0.126
ELSA	wave1_wave2	hibpe+diabe	2	200	176.67	1.13	0.166
ELSA	wave1_wave2	hibpe+stroke+arthre	3	51	41.11	1.24	0.236
ELSA	wave1_wave2	hibpe+lunge+arthre	3	63	55.67	1.13	0.484
ELSA	wave1_wave2	hibpe+psyche+arthre	3	98	91.85	1.07	0.649

ELSA	wave1_wave2	hibpe+cancre+arthre	3	92	92.16	1.00	1.000
ELSA	wave1_wave2	hibpe+stroke	2	63	70.34	0.90	1.000
ELSA	wave2_wave3	hibpe+diabe+hearte+arthre	4	68	24.88	2.73	1.43e-11
ELSA	wave2_wave3	hibpe+diabe+arthre	3	167	105.05	1.59	1.24e-07
ELSA	wave2_wave3	hibpe+hearte+arthre	3	280	200.37	1.40	2.95e-07
ELSA	wave2_wave3	hibpe+diabe+hearte	3	62	42.49	1.46	0.014
ELSA	wave2_wave3	hibpe+diabe	2	206	179.38	1.15	0.101
ELSA	wave2_wave3	hibpe+stroke+arthre	3	52	41.00	1.27	0.172
ELSA	wave2_wave3	hibpe+lunge+arthre	3	66	55.80	1.18	0.268
ELSA	wave2_wave3	hibpe+psyche+arthre	3	101	98.74	1.02	1.000
ELSA	wave2_wave3	hibpe+cancre+arthre	3	91	97.95	0.93	1.000
ELSA	wave2_wave3	hibpe+stroke	2	65	70.01	0.93	1.000
ELSA	wave3_wave4	hibpe+diabe+hearte+arthre	4	74	27.40	2.70	2.37e-12
ELSA	wave3_wave4	hibpe+diabe+arthre	3	154	101.59	1.52	6.18e-06
ELSA	wave3_wave4	hibpe+hearte+arthre	3	273	203.28	1.34	8.71e-06
ELSA	wave3_wave4	hibpe+diabe+hearte	3	68	45.10	1.51	0.004
ELSA	wave3_wave4	hibpe+diabe	2	182	167.23	1.09	0.480
ELSA	wave3_wave4	hibpe+cancre+arthre	3	103	100.59	1.02	1.000
ELSA	wave3_wave4	hibpe+psyche+arthre	3	95	95.02	1.00	1.000
ELSA	wave3_wave4	hibpe+stroke	2	61	61.86	0.99	1.000
ELSA	wave3_wave4	hibpe+lunge+arthre	3	51	52.11	0.98	1.000
ELSA	wave3_wave4	psyche+arthre	2	119	128.07	0.93	1.000
ELSA	wave4_wave5	hibpe+diabe+hearte+arthre	4	75	31.40	2.39	4.95e-10
ELSA	wave4_wave5	hibpe+hearte+psyche+arthre	4	50	30.30	1.65	0.003
ELSA	wave4_wave5	hibpe+diabe+arthre	3	134	99.33	1.35	0.003
ELSA	wave4_wave5	hibpe+hearte+arthre	3	264	215.44	1.23	0.003
ELSA	wave4_wave5	hibpe+diabe+hearte	3	64	45.14	1.42	0.017
ELSA	wave4_wave5	hibpe+diabe	2	168	142.80	1.18	0.065
ELSA	wave4_wave5	cancre+hearte+arthre	3	52	40.95	1.27	0.145
ELSA	wave4_wave5	hibpe+psyche+arthre	3	102	95.85	1.06	0.658
ELSA	wave4_wave5	hibpe+stroke	2	57	58.02	0.98	1.000
ELSA	wave4_wave5	psyche+arthre	2	113	119.63	0.94	1.000
HRS	wave1_wave2	hibpe+diabe+lunge+hearte+psyche+arthre	6	64	3.79	16.89	9.43e-53
HRS	wave1_wave2	hibpe+diabe+hearte+stroke+psyche+arthre	6	56	3.50	15.98	3.41e-45
HRS	wave1_wave2	hibpe+diabe+hearte+stroke+arthre	5	97	17.00	5.70	1.77e-39
HRS	wave1_wave2	hibpe+diabe+hearte+arthre	4	365	186.52	1.96	3.21e-30

HRS	wave1_wave2	hibpe+diabe+hearte+psyche+arthre	5	126	38.44	3.28	5.03e-28
HRS	wave1_wave2	hibpe+lunge+hearte+psyche+arthre	5	67	13.85	4.84	7.84e-24
HRS	wave1_wave2	hibpe+diabe+cancre+hearte+arthre	5	86	28.88	2.98	4.25e-17
HRS	wave1_wave2	hibpe+hearte+stroke+arthre	4	141	62.14	2.27	4.25e-17
HRS	wave1_wave2	hibpe+hearte+stroke+psyche+arthre	5	51	12.81	3.98	3.87e-15
HRS	wave1_wave2	hibpe+diabe+lunge+hearte+arthre	5	59	18.38	3.21	2.12e-13
HRS	wave2_wave3	hibpe+diabe+lunge+hearte+psyche+arthre	6	71	5.36	13.23	1.71e-51
HRS	wave2_wave3	hibpe+diabe+hearte+stroke+psyche+arthre	6	66	4.93	13.40	1.72e-48
HRS	wave2_wave3	hibpe+diabe+hearte+stroke+arthre	5	95	21.47	4.42	2.98e-30
HRS	wave2_wave3	hibpe+lunge+hearte+psyche+arthre	5	81	17.59	4.60	4.33e-27
HRS	wave2_wave3	hibpe+diabe+hearte+psyche+arthre	5	139	49.52	2.81	1.58e-24
HRS	wave2_wave3	hibpe+diabe+hearte+arthre	4	371	215.82	1.72	2.93e-21
HRS	wave2_wave3	hibpe+hearte+stroke+arthre	4	147	70.42	2.09	7.41e-15
HRS	wave2_wave3	hibpe+diabe+cancre+hearte+arthre	5	93	36.61	2.54	2.73e-14
HRS	wave2_wave3	hibpe+diabe+lunge+psyche+arthre	5	58	17.38	3.34	7.85e-14
HRS	wave2_wave3	hibpe+diabe+lunge+hearte+arthre	5	69	23.38	2.95	8.40e-14
HRS	wave3_wave4	hibpe+diabe+lunge+hearte+psyche+arthre	6	73	6.66	10.96	1.80e-47
HRS	wave3_wave4	hibpe+diabe+hearte+stroke+psyche+arthre	6	65	6.01	10.81	3.17e-42
HRS	wave3_wave4	hibpe+lunge+hearte+psyche+arthre	5	91	19.89	4.58	4.57e-30
HRS	wave3_wave4	hibpe+diabe+hearte+psyche+arthre	5	164	57.74	2.84	2.71e-29
HRS	wave3_wave4	hibpe+diabe+hearte+stroke+arthre	5	94	24.26	3.87	4.71e-26
HRS	wave3_wave4	hibpe+diabe+hearte+arthre	4	376	233.01	1.61	2.29e-17
HRS	wave3_wave4	hibpe+diabe+lunge+psyche+arthre	5	61	20.13	3.03	1.21e-12
HRS	wave3_wave4	hibpe+diabe+lunge+hearte+arthre	5	68	26.88	2.53	1.30e-10
HRS	wave3_wave4	hibpe+hearte+stroke+arthre	4	132	72.43	1.82	1.09e-09
HRS	wave3_wave4	hibpe+diabe+cancre+hearte+arthre	5	89	42.29	2.10	1.23e-09
HRS	wave4_wave5	hibpe+diabe+lunge+hearte+psyche+arthre	6	78	6.99	11.17	2.92e-51
HRS	wave4_wave5	hibpe+diabe+hearte+stroke+psyche+arthre	6	64	5.80	11.03	4.76e-42
HRS	wave4_wave5	hibpe+diabe+hearte+st	5	107	21.94	4.88	1.44e-37

		roke+arthre					
HRS	wave4_wave5	hibpe+diabe+hearte+psyche+arthre	5	176	60.27	2.92	9.65e-33
HRS	wave4_wave5	hibpe+lunge+hearte+psyche+arthre	5	92	19.71	4.67	3.29e-31
HRS	wave4_wave5	hibpe+diabe+hearte+arthre	4	382	227.83	1.68	3.98e-20
HRS	wave4_wave5	hibpe+diabe+cancre+hearte+arthre	5	104	37.55	2.77	2.85e-18
HRS	wave4_wave5	hibpe+hearte+stroke+psyche+arthre	5	60	16.37	3.66	5.58e-16
HRS	wave4_wave5	hibpe+diabe+lunge+hearte+arthre	5	78	26.41	2.95	1.91e-15
HRS	wave4_wave5	hibpe+diabe+lunge+psyche+arthre	5	68	23.48	2.90	3.10e-13
HRS	wave5_wave6	hibpe+diabe+lunge+hearte+psyche+arthre	6	79	8.66	9.12	1.05e-45
HRS	wave5_wave6	hibpe+diabe+hearte+stroke+psyche+arthre	6	69	6.83	10.10	5.65e-43
HRS	wave5_wave6	hibpe+lunge+hearte+psyche+arthre	5	86	21.55	3.99	1.62e-24
HRS	wave5_wave6	hibpe+diabe+hearte+stroke+arthre	5	88	23.60	3.73	2.84e-23
HRS	wave5_wave6	hibpe+diabe+hearte+psyche+arthre	5	163	68.00	2.40	9.18e-22
HRS	wave5_wave6	hibpe+diabe+cancre+hearte+arthre	5	103	42.06	2.45	1.20e-14
HRS	wave5_wave6	hibpe+diabe+hearte+arthre	4	354	234.91	1.51	1.31e-12
HRS	wave5_wave6	hibpe+diabe+lunge+psyche+arthre	5	72	26.54	2.71	1.43e-12
HRS	wave5_wave6	hibpe+hearte+stroke+psyche+arthre	5	54	17.00	3.18	3.66e-12
HRS	wave5_wave6	hibpe+diabe+lunge+hearte+arthre	5	75	29.92	2.51	1.51e-11

Table S13. Disease-count-preserving permutation null model for top-ranked BranchScore and LockInScore states.

For each transition, the observed from-state and target disease count were preserved, while the target disease-combination identity was randomized among compatible irreversible states. Observed top-10 BranchScore and LockInScore states were compared with 500 null permutations. Null percentile is the proportion of null scores less than or equal to the observed score.

Cohort	Window	Metric	Observed rank	State label	Disease count	From n	Observed score	Null median	Null 95th	Null percentile	Empirical upper p	> null 95th
CHARLS	wave1_wave2	BranchScore	1	lunge+hearte	2	55	0.352	0.380	0.396	0.042	0.958	No
CHARLS	wave1_wave2	BranchScore	2	hearte	1	331	0.320	0.347	0.351	0.000	1.000	No
CHARLS	wave1_wave2	BranchScore	3	stroke	1	51	0.273	0.273	0.286	0.838	0.164	No
CHARLS	wave1_wave2	BranchScore	4	diabe	1	206	0.233	0.249	0.253	0.000	1.000	No
CHARLS	wave1_wave2	BranchScore	5	hibpe+stroke	2	78	0.221	0.232	0.239	0.108	0.892	No
CHARLS	wave1_wave2	BranchScore	6	Healthy	0	6037	0.221	0.242	0.243	0.000	1.000	No
CHARLS	wave1_wav	BranchScor	7	hearte+arthr	2	263	0.216	0.226	0.229	0.004	0.996	No

	e2	e		e								
CHARLS	wave1_wave2	BranchScore	8	psyche	1	75	0.214	0.214	0.224	0.294	0.707	No
CHARLS	wave1_wave2	BranchScore	9	hibpe	1	1351	0.194	0.204	0.205	0.000	1.000	No
CHARLS	wave1_wave2	BranchScore	10	lunge	1	481	0.191	0.208	0.210	0.000	1.000	No
CHARLS	wave1_wave2	LockInScore	1	hibpe+diabe+hearte+arthre	4	56	4.034	4.034	4.034	0.244	0.756	No
CHARLS	wave1_wave2	LockInScore	2	hibpe+lunge+hearte+arthre	4	58	3.836	3.836	3.923	0.322	0.679	No
CHARLS	wave1_wave2	LockInScore	3	hibpe+lunge+arthre	3	96	3.177	3.165	3.201	0.858	0.144	No
CHARLS	wave1_wave2	LockInScore	4	hibpe+hearte+arthre	3	240	3.168	3.189	3.210	0.128	0.872	No
CHARLS	wave1_wave2	LockInScore	5	hibpe+diabe+arthre	3	66	3.043	3.158	3.223	0.208	0.792	No
CHARLS	wave1_wave2	LockInScore	6	hibpe+diabe+hearte	3	65	2.842	2.930	2.991	0.358	0.643	No
CHARLS	wave1_wave2	LockInScore	7	lunge+hearte+arthre	3	72	2.554	2.707	2.761	0.046	0.954	No
CHARLS	wave1_wave2	LockInScore	8	hibpe+arthre	2	755	2.430	2.444	2.461	0.096	0.904	No
CHARLS	wave1_wave2	LockInScore	9	hibpe+diabe	2	175	2.379	2.367	2.383	0.930	0.072	No
CHARLS	wave1_wave2	LockInScore	10	hearte+arthre	2	263	2.245	2.203	2.219	1.000	0.002	Yes
CHARLS	wave2_wave3	BranchScore	1	lunge	1	462	0.855	1.016	1.023	0.000	1.000	No
CHARLS	wave2_wave3	BranchScore	2	psyche	1	65	0.747	0.826	0.847	0.000	1.000	No
CHARLS	wave2_wave3	BranchScore	3	diabe	1	214	0.719	0.829	0.837	0.000	1.000	No
CHARLS	wave2_wave3	BranchScore	4	hearte	1	324	0.707	0.829	0.836	0.000	1.000	No
CHARLS	wave2_wave3	BranchScore	5	hibpe	1	1399	0.700	0.784	0.786	0.000	1.000	No
CHARLS	wave2_wave3	BranchScore	6	Healthy	0	5739	0.649	0.753	0.754	0.000	1.000	No
CHARLS	wave2_wave3	BranchScore	7	hibpe+diabe	2	182	0.634	0.666	0.673	0.002	0.998	No
CHARLS	wave2_wave3	BranchScore	8	hibpe+diabe+hearte	3	83	0.625	0.684	0.704	0.000	1.000	No
CHARLS	wave2_wave3	BranchScore	9	hibpe+hearte	2	314	0.569	0.628	0.634	0.000	1.000	No
CHARLS	wave2_wave3	BranchScore	10	diabe+arthre	2	112	0.551	0.631	0.643	0.000	1.000	No
CHARLS	wave2_wave3	LockInScore	1	hibpe+lunge+hearte+arthre	4	69	3.525	3.554	3.617	0.462	0.539	No
CHARLS	wave2_wave3	LockInScore	2	hibpe+diabe+hearte+arthre	4	59	2.810	2.912	2.971	0.172	0.844	No
CHARLS	wave2_wave3	LockInScore	3	lunge+hearte+arthre	3	87	2.490	2.468	2.513	0.812	0.190	No
CHARLS	wave2_wav	LockInScor	4	hibpe+diabe	3	85	2.265	2.306	2.358	0.162	0.838	No

	e3	e		+arthre								
CHARLS	wave2_wave3	LockInScore	5	hibpe+hearte+arthre	3	261	2.234	2.241	2.265	0.302	0.699	No
CHARLS	wave2_wave3	LockInScore	6	hibpe+lunge+arthre	3	107	2.158	2.068	2.119	0.990	0.012	Yes
CHARLS	wave2_wave3	LockInScore	7	hibpe+diabe+hearte	3	83	2.124	2.092	2.157	0.796	0.206	No
CHARLS	wave2_wave3	LockInScore	8	hibpe+arthre	2	789	2.004	1.961	1.991	0.994	0.008	Yes
CHARLS	wave2_wave3	LockInScore	9	lunge+arthre	2	294	2.001	1.986	2.011	0.794	0.208	No
CHARLS	wave2_wave3	LockInScore	10	hibpe+diabe	2	182	1.792	1.720	1.744	1.000	0.002	Yes
CHARLS	wave3_wave4	BranchScore	1	diabe+arthre	2	146	1.372	1.482	1.500	0.000	1.000	No
CHARLS	wave3_wave4	BranchScore	2	hearte	1	310	1.366	1.487	1.502	0.000	1.000	No
CHARLS	wave3_wave4	BranchScore	3	diabe	1	207	1.284	1.456	1.471	0.000	1.000	No
CHARLS	wave3_wave4	BranchScore	4	hibpe+lunge+hearte	3	76	1.264	1.343	1.373	0.008	0.992	No
CHARLS	wave3_wave4	BranchScore	5	stroke+arthre	2	57	1.257	1.328	1.368	0.036	0.964	No
CHARLS	wave3_wave4	BranchScore	6	hearte+arthre	2	323	1.223	1.332	1.343	0.000	1.000	No
CHARLS	wave3_wave4	BranchScore	7	hibpe	1	1261	1.187	1.256	1.261	0.000	1.000	No
CHARLS	wave3_wave4	BranchScore	8	hibpe+lunge	2	137	1.183	1.243	1.263	0.000	1.000	No
CHARLS	wave3_wave4	BranchScore	9	hibpe+hearte	2	322	1.182	1.271	1.282	0.000	1.000	No
CHARLS	wave3_wave4	BranchScore	10	lunge+arthre	2	414	1.114	1.219	1.227	0.000	1.000	No
CHARLS	wave3_wave4	LockInScore	1	hibpe+lunge+hearte+arthre	4	126	2.511	2.545	2.631	0.388	0.613	No
CHARLS	wave3_wave4	LockInScore	2	hibpe+diabe+hearte+arthre	4	94	2.018	2.040	2.156	0.440	0.561	No
CHARLS	wave3_wave4	LockInScore	3	hibpe+diabe+hearte	3	99	1.997	1.802	1.866	1.000	0.002	Yes
CHARLS	wave3_wave4	LockInScore	4	hibpe+hearte+arthre	3	362	1.972	1.973	2.010	0.462	0.539	No
CHARLS	wave3_wave4	LockInScore	5	hibpe+lunge+arthre	3	168	1.914	1.908	1.978	0.592	0.409	No
CHARLS	wave3_wave4	LockInScore	6	lunge+hearte+arthre	3	126	1.882	1.867	1.950	0.596	0.405	No
CHARLS	wave3_wave4	LockInScore	7	hibpe+arthre	2	947	1.747	1.718	1.736	1.000	0.002	Yes
CHARLS	wave3_wave4	LockInScore	8	hibpe+stroke+arthre	3	57	1.627	1.579	1.661	0.864	0.138	No
CHARLS	wave3_wave4	LockInScore	9	hibpe+lunge+hearte	3	76	1.536	1.356	1.436	1.000	0.002	Yes
CHARLS	wave3_wave4	LockInScore	10	lunge+arthre	2	414	1.521	1.462	1.480	1.000	0.002	Yes
CHARLS	wave4_wave5	BranchScore	1	hearte	1	390	0.949	1.042	1.052	0.000	1.000	No

CHARLS	wave4_wave5	BranchScore	2	hibpe+stroke	2	186	0.839	0.883	0.898	0.000	1.000	No
CHARLS	wave4_wave5	BranchScore	3	hibpe+lunge+hearte	3	114	0.749	0.812	0.829	0.000	1.000	No
CHARLS	wave4_wave5	BranchScore	4	lunge	1	484	0.742	0.813	0.820	0.000	1.000	No
CHARLS	wave4_wave5	BranchScore	5	hibpe+lunge	2	213	0.707	0.762	0.770	0.000	1.000	No
CHARLS	wave4_wave5	BranchScore	6	cancre+arthre	2	51	0.707	0.717	0.741	0.376	0.625	No
CHARLS	wave4_wave5	BranchScore	7	diabe	1	328	0.696	0.761	0.770	0.000	1.000	No
CHARLS	wave4_wave5	BranchScore	8	hibpe+hearte+stroke	3	78	0.693	0.762	0.781	0.000	1.000	No
CHARLS	wave4_wave5	BranchScore	9	stroke+arthre	2	86	0.685	0.728	0.749	0.010	0.990	No
CHARLS	wave4_wave5	BranchScore	10	psyche	1	82	0.643	0.621	0.643	0.936	0.066	No
CHARLS	wave4_wave5	LockInScore	1	hibpe+diabe+lunge+hearte+arthre	5	59	4.132	4.132	4.195	0.388	0.613	No
CHARLS	wave4_wave5	LockInScore	2	hibpe+diabe+hearte+arthre	4	137	3.519	3.476	3.538	0.888	0.114	No
CHARLS	wave4_wave5	LockInScore	3	hibpe+lunge+hearte+arthre	4	169	3.143	3.165	3.233	0.444	0.557	No
CHARLS	wave4_wave5	LockInScore	4	hibpe+diabe+lunge+arthre	4	70	3.049	2.876	2.949	1.000	0.002	Yes
CHARLS	wave4_wave5	LockInScore	5	hibpe+diabe+hearte	3	168	2.694	2.676	2.707	0.840	0.162	No
CHARLS	wave4_wave5	LockInScore	6	lunge+hearte+arthre	3	162	2.643	2.548	2.581	0.998	0.004	Yes
CHARLS	wave4_wave5	LockInScore	7	hibpe+hearte+arthre	3	418	2.614	2.444	2.529	1.000	0.002	Yes
CHARLS	wave4_wave5	LockInScore	8	hibpe+diabe+arthre	3	219	2.575	2.616	2.663	0.130	0.870	No
CHARLS	wave4_wave5	LockInScore	9	hibpe+hearte+stroke+arthre	4	80	2.526	2.368	2.454	0.996	0.006	Yes
CHARLS	wave4_wave5	LockInScore	10	hibpe+lunge+arthre	3	258	2.476	2.430	2.478	0.940	0.062	No
ELSA	wave1_wave2	BranchScore	1	diabe	1	129	0.507	0.542	0.552	0.000	1.000	No
ELSA	wave1_wave2	BranchScore	2	hibpe+psyche	2	119	0.412	0.410	0.419	0.596	0.423	No
ELSA	wave1_wave2	BranchScore	3	hearte	1	264	0.405	0.416	0.422	0.032	0.968	No
ELSA	wave1_wave2	BranchScore	4	psyche	1	206	0.322	0.333	0.338	0.016	0.984	No
ELSA	wave1_wave2	BranchScore	5	cancre+arthre	2	92	0.313	0.340	0.354	0.004	0.996	No
ELSA	wave1_wave2	BranchScore	6	lunge	1	92	0.306	0.312	0.322	0.206	0.794	No
ELSA	wave1_wave2	BranchScore	7	hibpe+diabe+arthre	3	148	0.304	0.314	0.318	0.068	0.932	No
ELSA	wave1_wave2	BranchScore	8	Healthy	0	2525	0.292	0.309	0.309	0.000	1.000	No

ELSA	wave1_wave2	BranchScore	9	hibpe+diabe	2	175	0.292	0.315	0.319	0.000	1.000	No
ELSA	wave1_wave2	BranchScore	10	hibpe+cancre+arthre	3	73	0.288	0.322	0.332	0.002	0.998	No
ELSA	wave1_wave2	LockInScore	1	hibpe+diabe+hearte+arthre	4	50	3.447	3.447	3.576	0.518	0.483	No
ELSA	wave1_wave2	LockInScore	2	hibpe+hearte+arthre	3	221	3.440	3.431	3.445	0.812	0.190	No
ELSA	wave1_wave2	LockInScore	3	hibpe+lunge+arthre	3	55	3.030	3.030	3.102	0.528	0.473	No
ELSA	wave1_wave2	LockInScore	4	hibpe+diabe+arthre	3	148	2.689	2.644	2.676	0.978	0.024	Yes
ELSA	wave1_wave2	LockInScore	5	hibpe+psyche+arthre	3	89	2.545	2.564	2.609	0.246	0.754	No
ELSA	wave1_wave2	LockInScore	6	hibpe+cancre+arthre	3	73	2.324	2.425	2.457	0.010	0.990	No
ELSA	wave1_wave2	LockInScore	7	hibpe+arthre	2	672	2.295	2.296	2.303	0.426	0.575	No
ELSA	wave1_wave2	LockInScore	8	hibpe+hearte	2	224	2.225	2.275	2.290	0.006	0.994	No
ELSA	wave1_wave2	LockInScore	9	hearte+arthre	2	182	2.169	2.199	2.217	0.054	0.946	No
ELSA	wave1_wave2	LockInScore	10	psyche+arthre	2	94	2.152	2.165	2.200	0.234	0.766	No
ELSA	wave2_wave3	BranchScore	1	psyche	1	207	0.432	0.441	0.448	0.074	0.926	No
ELSA	wave2_wave3	BranchScore	2	arthre	1	910	0.354	0.369	0.372	0.000	1.000	No
ELSA	wave2_wave3	BranchScore	3	hibpe+cancre	2	98	0.342	0.380	0.388	0.000	1.000	No
ELSA	wave2_wave3	BranchScore	4	lunge	1	82	0.323	0.348	0.358	0.022	0.978	No
ELSA	wave2_wave3	BranchScore	5	diabe	1	117	0.320	0.333	0.342	0.078	0.922	No
ELSA	wave2_wave3	BranchScore	6	hibpe+hearte	2	229	0.318	0.322	0.325	0.134	0.866	No
ELSA	wave2_wave3	BranchScore	7	hibpe+psyche+arthre	3	88	0.307	0.331	0.345	0.006	0.994	No
ELSA	wave2_wave3	BranchScore	8	Healthy	0	2489	0.299	0.316	0.317	0.000	1.000	No
ELSA	wave2_wave3	BranchScore	9	hearte+arthre	2	179	0.297	0.304	0.308	0.072	0.928	No
ELSA	wave2_wave3	BranchScore	10	hibpe	1	1017	0.288	0.302	0.304	0.000	1.000	No
ELSA	wave2_wave3	LockInScore	1	hibpe+diabe+hearte+arthre	4	51	4.123	4.123	4.123	0.000	1.000	No
ELSA	wave2_wave3	LockInScore	2	hibpe+hearte+arthre	3	215	2.976	3.009	3.033	0.052	0.948	No
ELSA	wave2_wave3	LockInScore	3	hibpe+diabe+arthre	3	137	2.901	2.852	2.877	0.996	0.006	Yes
ELSA	wave2_wave3	LockInScore	4	hibpe+cancre+arthre	3	73	2.811	2.799	2.863	0.688	0.313	No
ELSA	wave2_wave3	LockInScore	5	hibpe+psyche+arthre	3	88	2.664	2.764	2.794	0.002	0.998	No
ELSA	wave2_wave3	LockInScore	6	hibpe+arthre	2	641	2.267	2.219	2.241	1.000	0.002	Yes

ELSA	wave2_wave3	LockInScore	7	hibpe+stroke	2	55	2.138	2.087	2.138	0.772	0.230	No
ELSA	wave2_wave3	LockInScore	8	cancre+arthre	2	93	2.121	2.174	2.196	0.012	0.988	No
ELSA	wave2_wave3	LockInScore	9	hibpe+hearte	2	229	2.005	1.990	2.008	0.914	0.088	No
ELSA	wave2_wave3	LockInScore	10	psyche+arthre	2	107	1.974	1.994	2.020	0.210	0.790	No
ELSA	wave3_wave4	BranchScore	1	cancre	1	196	0.444	0.462	0.469	0.000	1.000	No
ELSA	wave3_wave4	BranchScore	2	hibpe	1	878	0.385	0.413	0.416	0.000	1.000	No
ELSA	wave3_wave4	BranchScore	3	hibpe+psyche	2	101	0.383	0.423	0.431	0.002	0.998	No
ELSA	wave3_wave4	BranchScore	4	lunge	1	72	0.359	0.373	0.388	0.200	0.800	No
ELSA	wave3_wave4	BranchScore	5	Healthy	0	2142	0.339	0.357	0.358	0.000	1.000	No
ELSA	wave3_wave4	BranchScore	6	hibpe+diabe+arthre	3	124	0.301	0.360	0.372	0.000	1.000	No
ELSA	wave3_wave4	BranchScore	7	psyche+arthre	2	112	0.295	0.317	0.325	0.008	0.992	No
ELSA	wave3_wave4	BranchScore	8	cancre+arthre	2	91	0.282	0.299	0.312	0.032	0.968	No
ELSA	wave3_wave4	BranchScore	9	hearte	1	253	0.278	0.297	0.300	0.000	1.000	No
ELSA	wave3_wave4	BranchScore	10	psyche	1	194	0.277	0.290	0.295	0.006	0.994	No
ELSA	wave3_wave4	LockInScore	1	hibpe+diabe+hearte+arthre	4	53	3.752	3.752	3.844	0.644	0.357	No
ELSA	wave3_wave4	LockInScore	2	hibpe+cancre+arthre	3	84	2.824	2.808	2.856	0.580	0.421	No
ELSA	wave3_wave4	LockInScore	3	hibpe+diabe+arthre	3	124	2.769	2.776	2.817	0.438	0.563	No
ELSA	wave3_wave4	LockInScore	4	hibpe+hearte+arthre	3	223	2.757	2.765	2.791	0.198	0.804	No
ELSA	wave3_wave4	LockInScore	5	hibpe+psyche+arthre	3	84	2.614	2.681	2.720	0.206	0.846	No
ELSA	wave3_wave4	LockInScore	6	hibpe+diabe+hearte	3	55	2.531	2.515	2.589	0.592	0.409	No
ELSA	wave3_wave4	LockInScore	7	hibpe+arthre	2	567	2.239	2.229	2.249	0.790	0.212	No
ELSA	wave3_wave4	LockInScore	8	hearte+arthre	2	158	2.234	2.258	2.276	0.114	0.886	No
ELSA	wave3_wave4	LockInScore	9	hibpe+hearte	2	218	2.112	2.096	2.116	0.914	0.088	No
ELSA	wave3_wave4	LockInScore	10	cancre+arthre	2	91	2.086	2.091	2.119	0.400	0.607	No
ELSA	wave4_wave5	BranchScore	1	diabe	1	98	0.596	0.603	0.616	0.272	0.729	No
ELSA	wave4_wave5	BranchScore	2	lunge	1	67	0.496	0.496	0.516	0.560	0.441	No
ELSA	wave4_wave5	BranchScore	3	hibpe+psyche+arthre	3	89	0.482	0.482	0.492	0.488	0.513	No
ELSA	wave4_wave5	BranchScore	4	psyche	1	159	0.442	0.487	0.496	0.000	1.000	No
ELSA	wave4_wav	BranchScor	5	hibpe	1	725	0.408	0.433	0.436	0.000	1.000	No

	e5	e										
ELSA	wave4_wave5	BranchScore	6	hearte	1	231	0.404	0.426	0.430	0.002	0.998	No
ELSA	wave4_wave5	BranchScore	7	cancre+arthre	2	92	0.397	0.421	0.431	0.016	0.984	No
ELSA	wave4_wave5	BranchScore	8	hibpe+psyche	2	82	0.372	0.387	0.398	0.088	0.912	No
ELSA	wave4_wave5	BranchScore	9	Healthy	0	1664	0.335	0.360	0.361	0.000	1.000	No
ELSA	wave4_wave5	BranchScore	10	hibpe+hearte	2	211	0.310	0.329	0.334	0.000	1.000	No
ELSA	wave4_wave5	LockInScore	1	hibpe+diabe+hearte+arthre	4	55	3.120	3.787	3.877	0.014	0.986	No
ELSA	wave4_wave5	LockInScore	2	hibpe+hearte+arthre	3	215	2.915	2.915	2.939	0.484	0.517	No
ELSA	wave4_wave5	LockInScore	3	hibpe+diabe+arthre	3	113	2.697	2.610	2.650	1.000	0.002	Yes
ELSA	wave4_wave5	LockInScore	4	hibpe+cancre+arthre	3	76	2.633	2.658	2.714	0.438	0.563	No
ELSA	wave4_wave5	LockInScore	5	hibpe+psyche+arthre	3	89	2.275	2.250	2.302	0.812	0.190	No
ELSA	wave4_wave5	LockInScore	6	psyche+arthre	2	98	2.216	2.255	2.275	0.030	0.970	No
ELSA	wave4_wave5	LockInScore	7	hibpe+hearte	2	211	2.136	2.076	2.095	1.000	0.002	Yes
ELSA	wave4_wave5	LockInScore	8	hibpe+arthre	2	517	2.081	2.041	2.050	1.000	0.002	Yes
ELSA	wave4_wave5	LockInScore	9	hibpe+diabe+hearte	3	52	2.064	2.239	2.298	0.052	0.948	No
ELSA	wave4_wave5	LockInScore	10	hearte+arthre	2	161	2.004	2.030	2.056	0.132	0.868	No
HRS	wave1_wave2	BranchScore	1	stroke+arthre	2	65	0.715	0.715	0.738	0.500	0.509	No
HRS	wave1_wave2	BranchScore	2	lunge+arthre	2	139	0.707	0.757	0.768	0.002	0.998	No
HRS	wave1_wave2	BranchScore	3	stroke	1	52	0.699	0.697	0.724	0.592	0.411	No
HRS	wave1_wave2	BranchScore	4	hibpe+diabe+lunge+arthre	4	62	0.606	0.606	0.627	0.466	0.535	No
HRS	wave1_wave2	BranchScore	5	psyche	1	317	0.569	0.643	0.651	0.000	1.000	No
HRS	wave1_wave2	BranchScore	6	hibpe+psyche	2	213	0.568	0.589	0.593	0.000	1.000	No
HRS	wave1_wave2	BranchScore	7	hibpe+stroke	2	99	0.534	0.568	0.582	0.008	0.992	No
HRS	wave1_wave2	BranchScore	8	hibpe+lunge	2	65	0.524	0.582	0.595	0.002	0.998	No
HRS	wave1_wave2	BranchScore	9	hibpe+hearte	2	318	0.482	0.503	0.508	0.000	1.000	No
HRS	wave1_wave2	BranchScore	10	hibpe+lunge+arthre	3	158	0.477	0.495	0.501	0.004	0.996	No
HRS	wave1_wave2	LockInScore	1	hibpe+diabe+lunge+hearte+psyche+arthre	6	53	5.025	5.025	5.025	1.000	0.002	Yes
HRS	wave1_wav	LockInScor	2	hibpe+heart	4	102	4.126	4.108	4.189	0.742	0.259	No

	e2	e		e+stroke+arthre								
HRS	wave1_wave2	LockInScore	3	hibpe+diabe+cancre+hearte+arthre	5	71	3.885	3.794	3.849	0.952	0.050	Yes
HRS	wave1_wave2	LockInScore	4	hibpe+diabe+hearte+psyche+arthre	5	112	3.883	3.825	3.906	0.894	0.108	No
HRS	wave1_wave2	LockInScore	5	hibpe+lunge+hearte+psyche+arthre	5	56	3.852	3.826	3.938	0.946	0.056	No
HRS	wave1_wave2	LockInScore	6	hibpe+lunge+hearte+arthre	4	94	3.738	3.712	3.738	0.982	0.100	No
HRS	wave1_wave2	LockInScore	7	hibpe+diabe+cancre+arthre	4	142	3.714	3.694	3.730	0.822	0.180	No
HRS	wave1_wave2	LockInScore	8	hibpe+cancre+hearte+arthre	4	120	3.695	3.682	3.715	0.730	0.271	No
HRS	wave1_wave2	LockInScore	9	hibpe+diabe+hearte+stroke+arthre	5	71	3.598	3.613	3.693	0.260	0.741	No
HRS	wave1_wave2	LockInScore	10	hibpe+diabe+psyche+arthre	4	182	3.503	3.512	3.550	0.242	0.758	No
HRS	wave2_wave3	BranchScore	1	lunge	1	70	0.788	0.817	0.841	0.076	0.926	No
HRS	wave2_wave3	BranchScore	2	diabe	1	273	0.752	0.879	0.888	0.000	1.000	No
HRS	wave2_wave3	BranchScore	3	hearte	1	209	0.614	0.662	0.670	0.000	1.000	No
HRS	wave2_wave3	BranchScore	4	hibpe+stroke	2	91	0.604	0.615	0.626	0.222	0.778	No
HRS	wave2_wave3	BranchScore	5	hibpe+lunge	2	56	0.560	0.606	0.627	0.008	0.992	No
HRS	wave2_wave3	BranchScore	6	lunge+psyche+arthre	3	54	0.554	0.560	0.583	0.284	0.717	No
HRS	wave2_wave3	BranchScore	7	hearte+psyche+arthre	3	54	0.517	0.508	0.522	0.736	0.287	No
HRS	wave2_wave3	BranchScore	8	hibpe+hearte	2	268	0.516	0.532	0.537	0.002	0.998	No
HRS	wave2_wave3	BranchScore	9	lunge+arthre	2	113	0.502	0.535	0.544	0.002	0.998	No
HRS	wave2_wave3	BranchScore	10	psyche	1	266	0.495	0.551	0.556	0.000	1.000	No
HRS	wave2_wave3	LockInScore	1	hibpe+diabe+hearte+stroke+psyche+arthre	6	51	5.336	5.336	5.336	0.272	0.729	No
HRS	wave2_wave3	LockInScore	2	hibpe+diabe+hearte+stroke+arthre	5	64	4.492	4.492	4.590	0.234	0.766	No
HRS	wave2_wave3	LockInScore	3	hibpe+diabe+lunge+hearte+arthre	5	56	4.331	4.331	4.435	0.584	0.417	No
HRS	wave2_wave3	LockInScore	4	hibpe+diabe+lunge+hearte+psyche+arthre	6	53	4.250	4.291	4.425	0.398	0.603	No
HRS	wave2_wave3	LockInScore	5	hibpe+cancre+psyche+arthre	4	81	4.105	4.105	4.176	0.640	0.361	No

HRS	wave2_wave3	LockInScore	6	hibpe+diabe+hearte+arthre	4	322	4.052	4.025	4.050	0.980	0.022	Yes
HRS	wave2_wave3	LockInScore	7	hibpe+diabe+cancre+arthre	4	134	3.923	3.946	3.976	0.264	0.737	No
HRS	wave2_wave3	LockInScore	8	hibpe+diabe+cancre+hearte+arthre	5	74	3.840	3.840	3.951	0.530	0.471	No
HRS	wave2_wave3	LockInScore	9	hibpe+diabe+hearte+psyche+arthre	5	118	3.771	3.777	3.866	0.410	0.591	No
HRS	wave2_wave3	LockInScore	10	hibpe+lunge+hearte+psyche+arthre	5	65	3.665	3.665	3.775	0.160	0.840	No
HRS	wave3_wave4	BranchScore	1	lunge	1	51	0.952	0.959	0.988	0.460	0.561	No
HRS	wave3_wave4	BranchScore	2	lunge+arthre	2	95	0.849	0.909	0.928	0.004	0.996	No
HRS	wave3_wave4	BranchScore	3	psyche	1	221	0.714	0.763	0.773	0.000	1.000	No
HRS	wave3_wave4	BranchScore	4	hibpe+stroke	2	76	0.638	0.668	0.683	0.040	0.962	No
HRS	wave3_wave4	BranchScore	5	hibpe+stroke+arthre	3	125	0.595	0.601	0.612	0.218	0.786	No
HRS	wave3_wave4	BranchScore	6	hibpe+lunge+hearte+arthre	4	88	0.592	0.591	0.609	0.516	0.485	No
HRS	wave3_wave4	BranchScore	7	diabe	1	215	0.572	0.670	0.676	0.000	1.000	No
HRS	wave3_wave4	BranchScore	8	hibpe+hearte	2	241	0.559	0.585	0.592	0.000	1.000	No
HRS	wave3_wave4	BranchScore	9	Healthy	0	2195	0.558	0.609	0.611	0.000	1.000	No
HRS	wave3_wave4	BranchScore	10	hearte	1	169	0.549	0.577	0.586	0.002	0.998	No
HRS	wave3_wave4	LockInScore	1	hibpe+diabe+hearte+stroke+arthre	5	67	4.302	4.254	4.302	0.882	0.120	No
HRS	wave3_wave4	LockInScore	2	hibpe+diabe+cancre+hearte+arthre	5	67	4.269	4.303	4.359	0.372	0.629	No
HRS	wave3_wave4	LockInScore	3	hibpe+diabe+hearte+psyche+arthre	5	129	4.167	4.160	4.226	0.674	0.327	No
HRS	wave3_wave4	LockInScore	4	hibpe+diabe+psyche+arthre	4	201	3.631	3.621	3.657	0.664	0.337	No
HRS	wave3_wave4	LockInScore	5	hibpe+cancre+hearte+arthre	4	116	3.507	3.455	3.507	0.954	0.092	No
HRS	wave3_wave4	LockInScore	6	hibpe+diabe+hearte+arthre	4	300	3.330	3.336	3.358	0.202	0.798	No
HRS	wave3_wave4	LockInScore	7	hibpe+diabe+lunge+psyche+arthre	5	53	3.192	3.136	3.229	0.722	0.279	No
HRS	wave3_wave4	LockInScore	8	hibpe+hearte+stroke+arthre	4	92	3.157	3.186	3.244	0.178	0.822	No
HRS	wave3_wave4	LockInScore	9	hibpe+lunge+psyche+arthre	4	66	3.051	3.042	3.096	0.582	0.419	No
HRS	wave3_wav	LockInScor	10	hibpe+diabe	4	65	2.994	3.038	3.086	0.206	0.794	No

	e4	e		+lunge+arthre								
HRS	wave4_wave5	BranchScore	1	hibpe+stroke	2	77	0.822	0.860	0.879	0.028	0.972	No
HRS	wave4_wave5	BranchScore	2	lunge	1	59	0.717	0.841	0.870	0.000	1.000	No
HRS	wave4_wave5	BranchScore	3	psyche	1	280	0.587	0.669	0.677	0.000	1.000	No
HRS	wave4_wave5	BranchScore	4	hibpe+lunge	2	57	0.576	0.600	0.617	0.122	0.878	No
HRS	wave4_wave5	BranchScore	5	hibpe+hearte	2	238	0.526	0.554	0.560	0.000	1.000	No
HRS	wave4_wave5	BranchScore	6	hibpe+diabe+cancre	3	60	0.515	0.582	0.595	0.002	0.998	No
HRS	wave4_wave5	BranchScore	7	hibpe+psyche	2	198	0.494	0.539	0.546	0.000	1.000	No
HRS	wave4_wave5	BranchScore	8	cancre	1	206	0.490	0.552	0.559	0.000	1.000	No
HRS	wave4_wave5	BranchScore	9	diabe+arthre	2	190	0.490	0.512	0.518	0.004	0.996	No
HRS	wave4_wave5	BranchScore	10	hibpe+lunge+arthre	3	136	0.489	0.497	0.505	0.168	0.832	No
HRS	wave4_wave5	LockInScore	1	hibpe+diabe+hearte+psyche+arthre	5	135	4.770	4.892	4.939	0.162	0.838	No
HRS	wave4_wave5	LockInScore	2	hibpe+diabe+lunge+hearte+psyche+arthre	6	62	4.582	4.582	4.696	0.368	0.633	No
HRS	wave4_wave5	LockInScore	3	hibpe+diabe+cancre+hearte+arthre	5	80	4.307	4.321	4.414	0.204	0.796	No
HRS	wave4_wave5	LockInScore	4	hibpe+diabe+hearte+stroke+arthre	5	73	4.216	4.198	4.216	0.672	0.329	No
HRS	wave4_wave5	LockInScore	5	hibpe+lunge+hearte+psyche+arthre	5	71	4.037	3.969	4.037	0.852	0.150	No
HRS	wave4_wave5	LockInScore	6	hibpe+cancre+hearte+arthre	4	107	3.761	3.761	3.803	0.388	0.613	No
HRS	wave4_wave5	LockInScore	7	hibpe+diabe+hearte+arthre	4	311	3.726	3.734	3.798	0.266	0.735	No
HRS	wave4_wave5	LockInScore	8	hibpe+diabe+stroke+arthre	4	56	3.609	3.696	3.759	0.372	0.629	No
HRS	wave4_wave5	LockInScore	9	hibpe+hearte+psyche+arthre	4	131	3.531	3.541	3.580	0.240	0.760	No
HRS	wave4_wave5	LockInScore	10	hibpe+diabe+cancre+arthre	4	115	3.451	3.388	3.438	0.956	0.046	Yes
HRS	wave5_wave6	BranchScore	1	lunge+arthre	2	86	0.548	0.590	0.605	0.010	0.990	No
HRS	wave5_wave6	BranchScore	2	diabe	1	241	0.544	0.614	0.619	0.000	1.000	No
HRS	wave5_wave6	BranchScore	3	hibpe+hearte	2	194	0.513	0.552	0.558	0.000	1.000	No
HRS	wave5_wave6	BranchScore	4	hearte	1	131	0.503	0.540	0.547	0.002	0.998	No
HRS	wave5_wav	BranchScor	5	hibpe+psyc	2	161	0.471	0.502	0.509	0.000	1.000	No

	e6	e		he								
HRS	wave5_wave6	BranchScore	6	cancre	1	171	0.457	0.509	0.515	0.000	1.000	No
HRS	wave5_wave6	BranchScore	7	hibpe	1	1168	0.445	0.478	0.480	0.000	1.000	No
HRS	wave5_wave6	BranchScore	8	psyche	1	221	0.419	0.449	0.454	0.000	1.000	No
HRS	wave5_wave6	BranchScore	9	diabe+arthre	2	174	0.390	0.462	0.467	0.000	1.000	No
HRS	wave5_wave6	BranchScore	10	cancre+arthre	2	165	0.386	0.401	0.409	0.024	0.976	No
HRS	wave5_wave6	LockInScore	1	hibpe+diabe+cancre+hearte+arthre	5	71	4.808	4.808	4.901	0.782	0.220	No
HRS	wave5_wave6	LockInScore	2	hibpe+diabe+lunge+hearte+psyche+arthre	6	58	4.466	5.096	5.154	0.000	1.000	No
HRS	wave5_wave6	LockInScore	3	hibpe+diabe+lunge+psyche+arthre	5	59	4.438	4.438	4.622	0.584	0.417	No
HRS	wave5_wave6	LockInScore	4	hibpe+hearte+stroke+arthre	4	67	4.295	4.295	4.295	0.000	1.000	No
HRS	wave5_wave6	LockInScore	5	hibpe+lunge+hearte+psyche+arthre	5	62	4.200	4.335	4.366	0.282	0.719	No
HRS	wave5_wave6	LockInScore	6	hibpe+diabe+hearte+stroke+arthre	5	58	4.172	4.247	4.281	0.248	0.752	No
HRS	wave5_wave6	LockInScore	7	hibpe+cancre+hearte+arthre	4	113	3.991	3.978	3.991	0.956	0.046	Yes
HRS	wave5_wave6	LockInScore	8	hibpe+diabe+hearte+psyche+arthre	5	124	3.885	3.851	3.937	0.756	0.246	No
HRS	wave5_wave6	LockInScore	9	hibpe+diabe+hearte+arthre	4	278	3.802	3.789	3.813	0.846	0.156	No
HRS	wave5_wave6	LockInScore	10	hibpe+cancre+psyche+arthre	4	72	3.498	3.460	3.498	0.970	0.032	Yes
